# Exploring the Nuclearity and Structural Motifs of Phenoxyimine Alkaline Earth Complexes

**DOI:** 10.1021/acs.organomet.4c00504

**Published:** 2025-03-03

**Authors:** Amy V. Rizzo, Rebecca L. Jones, Matthew D. Haynes, Clement G. Collins Rice, Jean-Charles Buffet, Zoë R. Turner, Dermot O’Hare

**Affiliations:** Chemistry Research Laboratory, Department of Chemistry, University of Oxford, Mansfield Road, Oxford OX1 3TA, U.K.

## Abstract

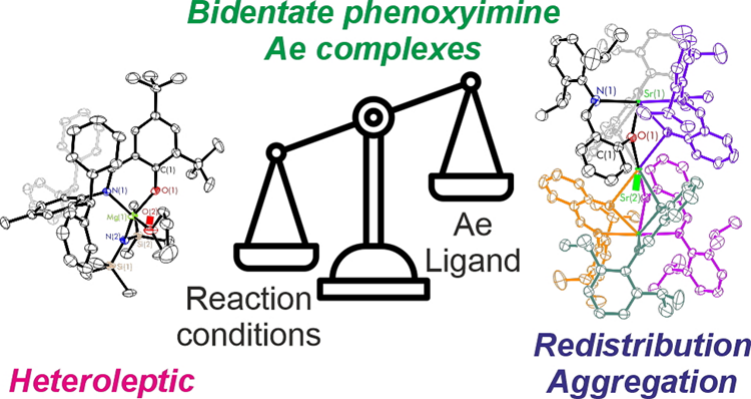

The nuclearity and structural motifs of alkaline earth complexes supported by bidentate phenoxyimine ligands has been explored by modulation of the stereoelectronic profile of the ligand, the atomic number of the metal, and the synthetic protocol. Changing the size of the *N*-imine substituents was found to have no effect on protonolysis reactions between [MgN″_2_]_2_ or CaN″_2_(thf)_2_ (N″ = N(SiMe_3_)_2_) and H^*^t^*Bu_2_,Ar^L (1-OH-2-CH = NAr-4,6-*^t^*Bu-C_6_H_2_; Ar = 2,6-^i^Pr–C_6_H_3_ = Dipp or 2,6-CHPh_2_-4-Me-C_6_H_2_ = Ar*) regardless of reaction stoichiometry, with homoleptic *bis*(ligand) complexes (^*^t^*Bu_2_,Dipp^L)_2_Mg (**1**), (^*^t^*Bu_2_,Ar*^L)_2_Mg (**2**), (^*^t^*Bu_2_,Dipp^L)_2_Ca(thf) (**3**) and (^*^t^*Bu_2_,Ar*^L)_2_Ca(thf) (**4**) isolated. The importance of reaction protocol was demonstrated by the facile isolation of heteroleptic complex (^*^t^*Bu_2_,Ar*^L)MgI(OEt_2_) (**5**) from the reaction of equimolar amounts of H^*^t^*Bu_2_,Ar*^L and MeMgI. Importantly, no subsequent ligand redistribution was observed when complex **5** readily reacted with KN” or KODipp to form (^*^t^*Bu_2_,Ar*^L)Mg{N(SiMe_3_)_2_}(OEt_2_) (**6**) and (^*^t^*Bu_2_,Ar*^L)Mg(ODipp)(thf) (**7**). When small 4,6-phenoxide substituents were considered (H^H_2_,Dipp^L), multimetallic clusters were afforded: (^H_2_,Dipp^L)_3_Ca_2_(N″)(thf) (**8**) and (^H_2_,Dipp^L)_6_Sr_3_ (**9**).

## Introduction

The use of alkaline earth (Ae = Mg, Ca, Sr, Ba) complexes in both catalytic and stoichiometric transformations offers many advantages as a result of being inexpensive, earth-abundant and biocompatible.^1^ However, the propensity of heteroleptic complexes LAeX (L = ancillary ligand, X = simple monoanionic donor; e.g., halide, amide, alkoxide, hydride) to undergo ligand redistribution to the homoleptic L_2_M and MX_2_ as part of a generalized Schlenk equilibrium still remains a challenging part of controlling Ae chemistry.^2^ Utilization of a sterically demanding monoanionic ligand L, particularly for heavier alkaline earth metal complexes (Ca, Sr and Ba) has proven to be a suitable technique for the kinetic stabilization and subsequent isolation of the desired heteroleptic species.^3^ Despite this, there is no prescriptive approach to generating isolable yet reactive LAeX complexes; the increasingly large coordination sphere as group 2 is descended means that a wide array of structural motifs can be generated, including multimetallic clusters and polymeric systems.^4^

Phenoxyimine (Schiff-base) ligands have been widely utilized across a wide range of metals including transition, alkali, and alkaline earth metals for catalytic applications including bond activation, polymerizations, and in organic reactions,^5^ due to their ease of preparation and modification, high yields, and high thermal stability. One prominent challenge in using Schiff-base alkaline earth complexes as catalysts is that they tend to aggregate in solution, which can reduce reactivity, when not sufficiently sterically shielded. A number of solid-state structures of these multimetallic species have been reported, although often their formation is accidental in the pursuit of single-site catalysts.^6^

**Chart 1:**
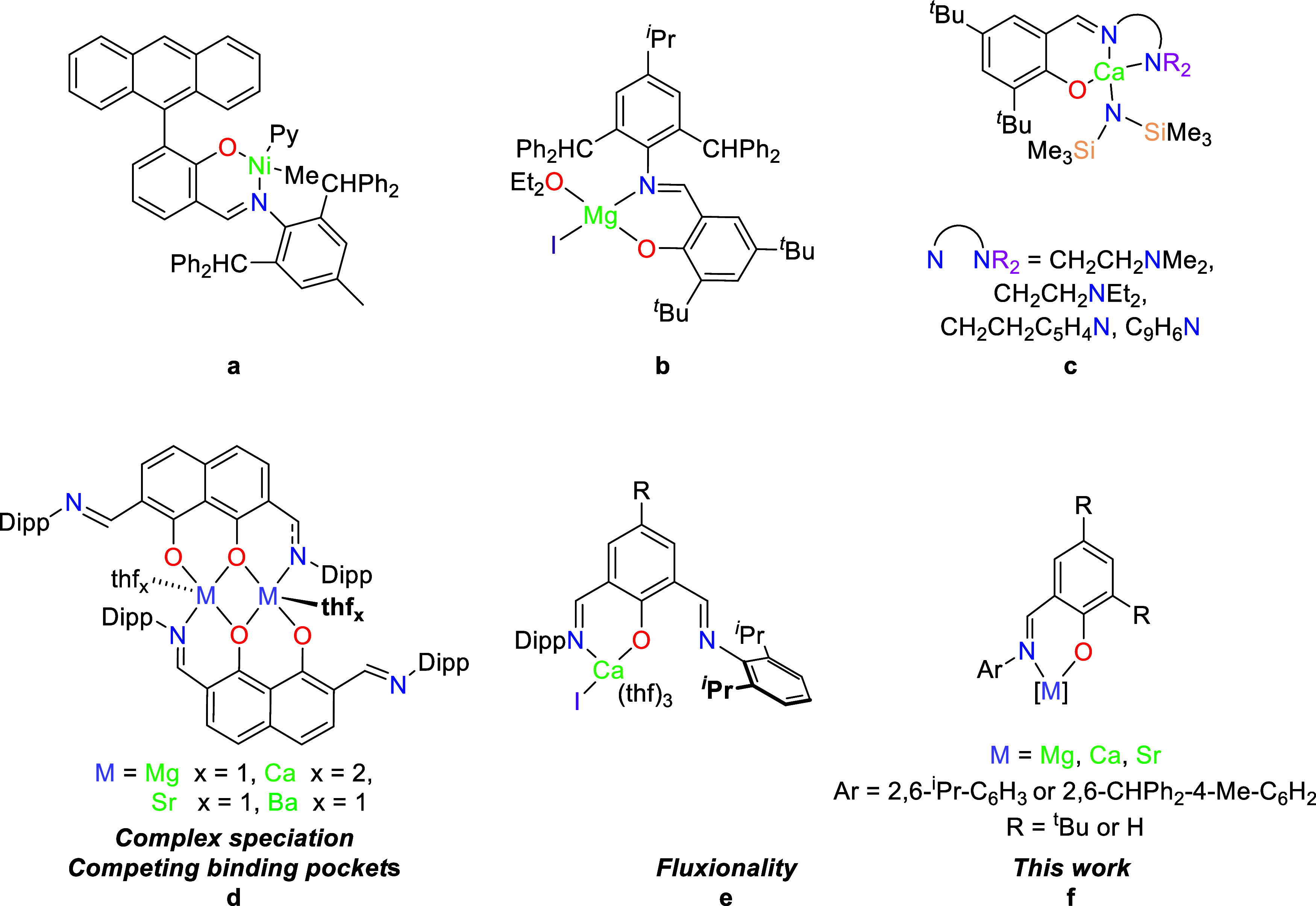
Strategies for Isolating M^2+^ Heteroleptic Schiff-Base Complexes Exemplified by (a) Mecking and Co-Workers,^10^ (b) Ma and Co-Workers,^8^ and (c) Darensbourg and Co-Workers;^9a^ (d) Phenoxyimine NOON and (e) NON Alkaline Earth Complexes Reported by Jones *et al*.,^13^ and (f) This Work.

To attempt to favor heteroleptic species using phenoxyimine ligands, the 6-position of phenoxide ring can be modulated,^7^ as can the *N*-imine substituent,^8^ though this typically involves incorporating pendent donors to saturate the metal coordination sphere.^6b,9^ In transition metal chemistry, Mecking and co-workers utilized an anthracenyl-substituted framework to prepare nickel complexes and probe the effect of complex stereochemistry in ethylene polymerization (Chart 1a).^10^ Ma *et al*. reported that the treatment of a bulky phenoxyimine ligand with diphenylmethyl substituents with MeMgI, proved to be a viable synthetic approach to a heteroleptic magnesium species (Chart 1b).^8^ The complex was subsequently reduced with Na to produce a Mg–Na heterobimetallic complex which was used to hydrosilylate ketones. Darensbourg *et al*. reported calcium complexes supported by a phenoxyimime ligand with a pendent amine donor (Chart 1c); the derivative with a pendent dimethylamine was found to be very effective at polymerizing both *rac*-LA and trimethyl carbonate.^9a^

Reports of systematic investigation of the effects of both ligand steric profile and conditions for synthesis have thus far been limited to magnesium. Rood and co-workers depicted how the degree of aggregation and the coordination number of *bis*(salicylaldiminato) magnesium complexes was reduced by increasing substitution of the ligand.^11^ The same team also found that reaction solvent was an important factor that affected the outcome of reactions between MgN″_2_ (N″ = N(SiMe_3_)_2_) and phenoxyimine proligands, with nondonor solvents leading to heteroleptic species and donor solvents leading to homoleptic species that could be isolated in the solid-state.^12^

We have recently reported the synthesis and characterization of alkaline earth complexes (Mg–Ba) supported by a *bis*(phenoxyimine) NOON ligand with a rigid naphthalene backbone.^13a^ With competing binding pockets, the Ae^2+^ ions were preferentially bound to the central [O^–^,O^–^] site (Chart 1d); complexes of the heavier metals were found to exist as mixtures of monomeric and dimeric species, which were still active initiators for the polymerization of lactide.^13b^ Subsequently, we have described a family of heteroleptic NON phenoxyimine calcium complexes which could be isolated as well-defined complexes, though there was spectroscopic evidence for the Ca^2+^ hopping between the two potential [=N, O^–^] binding pockets (Chart 1e).^13c^ The complexes were proposed to polymerize lactide via a ligand assisted, activated monomer mechanism.

Herein, we report our study into the structural motifs and corresponding nuclearity of bidentate phenoxyimine alkaline earth metal complexes with only a single binding pocket (Chart 1f). This has been approached by descending group 2 (Mg–Sr), modulating the steric profile of the phenoxyimine ligand through both the imine and the phenolic components, and also considering the effect of reaction conditions on the outcome of the isolable species.

## Results and Discussion

### Modulating the Imine Substituent; Homoleptic Schiff-Base Alkaline Earth *bis*(Ligand) Complexes

**Scheme 1:**
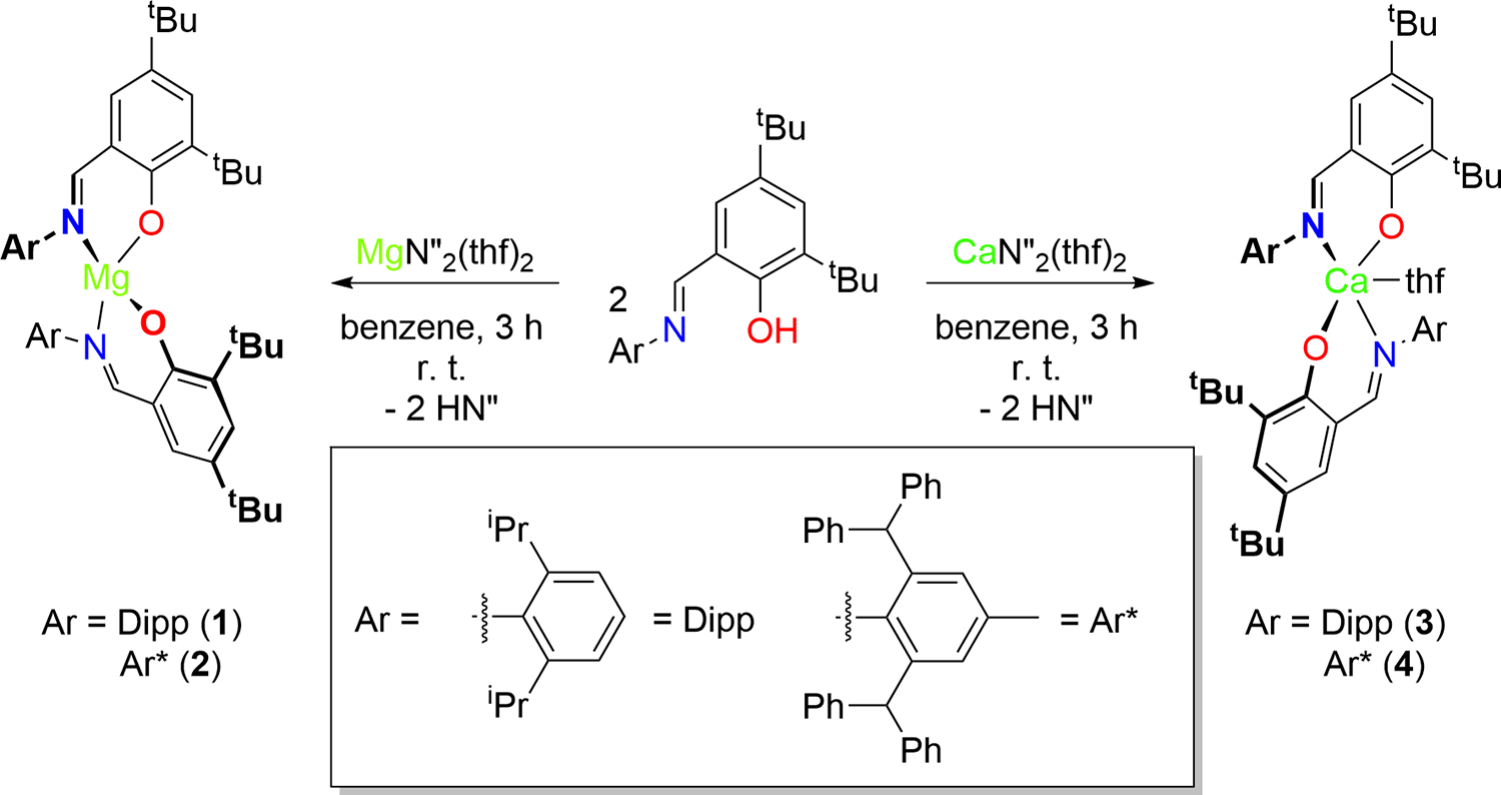
Protonolysis Reactions between H^*^t^*Bu_2_,Ar^L and MN″_2_(thf)_2_ (M = Mg and Ca) Affording (^*^t^*Bu_2_,Dipp^L)_2_Mg (**1**), (^*^t^*Bu_2_,Ar*^L)_2_Mg (**2**), (^*^t^*Bu_2_,Dipp^L)_2_Ca(thf) (**3**) and (^*^t^*Bu_2_,Ar*^L)_2_Ca(thf) (**4**) Relative ligand orientations depicted consistent with solid-state structural data.

Protonolysis reactions of H^*^t^*Bu_2_,Ar^L (1-OH-2-CH = NAr-4,6-*^t^*Bu-C_6_H_2_; Ar = 2,6-^*i*^Pr–C_6_H_3_ = Dipp or 2,6-CHPh_2_-4-Me-C_6_H_2_ = Ar*) with the metal silylamides [MgN″_2_]_2_ and CaN″_2_(thf)_2_ (N″ = N(SiMe_3_)_2_) in a 1:2 (M/L) molar ratio afforded the corresponding homoleptic *bis*(ligand) complexes (^*^t^*Bu_2_,Dipp^L)_2_Mg (**1**),^11,14^ (^*^t^*Bu_2_,Ar*^L)_2_Mg (**2**), (^*^t^*Bu_2_,Dipp^L)_2_Ca(thf) (**3**) and (^*^t^*Bu_2_,Ar*^L)_2_Ca(thf) (**4**) as yellow powders in 46–74% isolated yields (Scheme 1).

When the reactants were combined in a 1:1 ratio, the same metal complexes **1**–**4** were obtained alongside 1 equivalent of unreacted metal silylamide (Figure S11). Varying the reaction conditions by cooling the reaction mixture, using high dilution conditions or by using a slow rate of addition of proligand to metal precursor did not afford a heteroleptic complex as the major species, suggesting that rapid ligand redistribution was occurring in all cases to give a *bis*(ligand) system. Additionally, for Ca, carrying out the 1:1 reaction in the presence of chelating donor like dimethoxethane led to a mixture of products. Inclusion of strong donors like dimethylaminopyridine (DMAP) led to the formation of (^*^t^*Bu_2_,Dipp^L)_2_Ca(DMAP)_2_, which was characterized in the solid state (Figure S39 and Table S2).

The ^1^H NMR spectra of (^*^t^*Bu_2_,Dipp^L)_2_Mg (**1**) and (^*^t^*Bu_2_,Dipp^L)_2_Ca(thf) (**3**) in benzene-*d*_6_ contain the same diagnostic features. Both are consistent with the ligands being related by a *C*_2_ axis in solution (Figures S1 and S12). For example, for complex **1** the ^1^H NMR spectrum features a characteristic singlet at 7.91 ppm for the *H*C=N protons. Doublets at 7.71 and 6.85 ppm appear due to ^4^*J*_H–H_ coupling between the 3-C_6_*H*_2_ and 5-C_6_*H*_2_ protons. The remaining aromatic signals at 7.01 and 6.92 ppm, integrating to a total of 6H, represent the N–C_6_*H*_3_ protons. Restricted rotation of the Dipp groups is observed by the splitting out of the signals associated with the methine and methyl protons; methyl groups are observed as four doublets in the range 1.22–0.39 ppm which each integrate to 6H, while the methine protons are represented by two septets at 2.62 and 3.69 ppm, each with ^3^*J*_H–H_ = 6.8 Hz.

In contrast, for (^*^t^*Bu_2_,Dipp^L)_2_Ca(thf) (**3**) the Dipp methyl protons are represented by a single doublet, integrating to a total of 24H, at 1.16 ppm, and the methine protons are indicated as a single septet with ^3^*J*_H–H_ = 6.9 Hz at 3.12 ppm. In addition, the ^1^H NMR spectrum of complex **3** features two multiplets at 3.59 and 1.23 ppm corresponding to the protons of the bound thf molecule.

The ^1^H NMR spectra of (^*^t^*Bu_2_,Ar*^L)_2_Mg (**2**) and (^*^t^*Bu_2_,Ar*^L)_2_Ca(thf) (**4**) contain the same characteristic features. For example, for complex **4**, the imine *H*C=N signal appears at 6.26 ppm. Multiplets in the range 6.93–6.62 ppm, which integrate to a total of 44H, represent the aromatic N-2,6–CH(C_6_*H*_5_)_2_ and N–C_6_*H*_2_ protons. These aromatic resonances are broadened for the calcium species, in contrast to the sharp signals observed for the magnesium analogue (See Figures S6 and S17). The remaining aromatic protons are depicted by two mutually coupled doublets (^4^*J*_H–H_ = 2.8 Hz) at 7.56 and 5.60 ppm that correspond to the 3-C_6_*H*_2_ and 5-C_6_*H*_2_ protons, and a broad singlet at 5.70 ppm, which integrates to 4H and represents the C*H*(C_6_H_5_)_2_ protons. Exchange processes also result in broadened thf signals located at 3.95 and 1.06 ppm.

**Figure 1:**
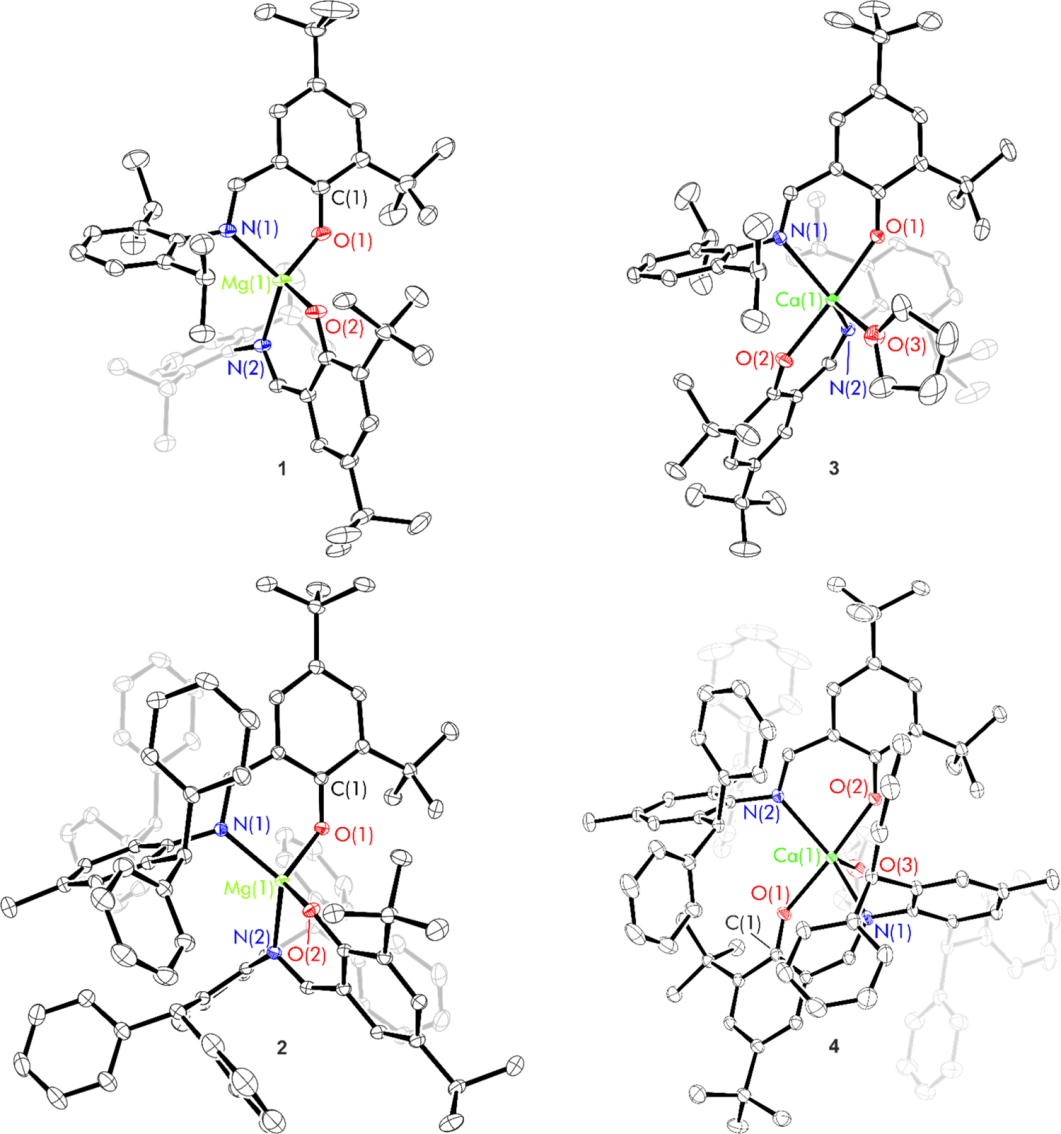
Thermal displacement ellipsoid drawings (30% probability) of (^*^t^*Bu_2_,Dipp^L)_2_Mg (**1**), (^*^t^*Bu_2_,Ar*^L)_2_Mg (**2**), (^*^t^*Bu_2_,Dipp^L)_2_Ca(thf) (**3**) and (^*^t^*Bu_2_,Ar*^L)_2_Ca(thf) (**4**). All hydrogen atoms and solvent of recrystallization are omitted for clarity.

Single crystals of complexes **1**–**4**, suitable for X-ray diffraction studies, were obtained by the slow evaporation of saturated benzene solutions at room temperature (Figure 1 and Table 1). Crystals of complex **1**, with no cocrystallized solvent have previously been analyzed using synchrotron radiation by Rood, Oliver and co-workers; small variations in the metrical parameters are observed between both structures.^11^ All complexes crystallized as monomeric, homoleptic species.

**Table 1:** Selected Metrical Data for (^*^t^*Bu_2_,Dipp^L)_2_Mg (1), (^*^t^*Bu_2_,Ar*^L)_2_Mg (2), (^*^t^*Bu_2_,Dipp^L)_2_Ca(thf) (3) and (^*^t^*Bu_2_,Ar*^L)_2_Ca(thf) (4)^a^^,^^b^

complex	(^*^t^*Bu_2_,Dipp^L)_2_ Mg (**1**)	(^*^t^*Bu_2_,Ar*^L)_2_ Mg (**2**)	(^*^t^*Bu_2_,Dipp^L)_2_ Ca(thf) (**3**)	(^*^t^*Bu_2_,Ar*^L)_2_ Ca(thf) (**4**)	(^*^t^*Bu_2_,Ar^Mes*^^L)_2_ Mg^21^	(^6-*^t^*Bu,Ar^F^^L)_2_ Ca(thf)_2_^20^
M(1)-N(1)	2.0860(17)	2.0617(12)	2.4542(15)	2.4665(12)	2.1814(13)	2.2229(18)
M(1)-N(2)	2.0880(16)	2.0887(12)	2.4564(15)	2.4532(13)	2.1814(13)	2.2229(18)
M(1)-O(1)	1.8950(14)	1.9085(11)	2.1953(13)	2.1764(11)	1.9596(10)	2.479(2)
M(1)-O(2)	1.8899(15)	1.8988(11)	2.1869(13)	2.1889(10)	1.9596(10)	2.479(2)
M(1)-O(3)	-	-	2.329(8)	2.3439(12)	-	2.3369(19)
O(1)-M(1)-N(1)	92.58(6)	91.21(5)	75.41(5)	75.96(4)	85.54(5)	74.08(7)
N(1)-M(1)-N(2)	117.91(7)	124.44(5)	135.75(5)	131.72(4)	180.0	180.0
O(1)-M(1)-O(2)	110.28(7)	115.78(5)	175.31(6)	174.70(4)	180.0	180.0

a Bond lengths in Å and bond angles in °.

b [^*^t^*Bu_2_,Ar^Mes*^^L]– = [O-2-{C(H)=N-2,4,6-*^t^*Bu-C_6_H_2_}-4,6-*^t^*Bu-C_6_H_2_]–, [^6-*^t^*Bu,Ar^F^^L]– = [O-2-{C(H)=N–C_6_F_5_}-6-*^t^*Bu-C_6_H_3_)].

The solid-state structures of the magnesium complexes feature distorted tetrahedral geometries around the metal center (τ_4_ = 0.81 and 0.82 for **1** and **2**, respectively; where τ_4_ = 0 for an idealized square planar geometry and τ_4_ = 1 for idealized tetrahedral geometry).^15^ The *N*-Dipp substituents in **1** and **2** are splayed with respect to one another in order to minimize unfavorable steric interactions. This molecular *C*_1_ symmetric arrangement has previously been observed in related complexes, such as (O-2-{C(H)=N-2,6-^i^Pr–C_6_H_3_,-6-*^t^*Bu-C_6_H_3_)_2_Ni},^16^ and contrasts with the behavior of the system in solution. While distorted tetrahedral (τ_4_ = 0.76–0.92) geometry is common among reported 4-coordinate Mg(II) complexes,^6a,11^ this is not always the case. An unusual example is (^*^t^*Bu_2_,Ar^Mes*^^L)_2_Mg (where L = [O-2-{C(H)=N-2,4,6-*^t^*Bu-C_6_H_2_}-4,6-^*t*^Bu-C_6_H_2_]^−^ and 2,4,6-^*t*^Bu-C_6_H_2_ = Mes*),^8^ which is observed to have perfect square planar geometry (τ_4_ = 0) with the N and O donors *trans* to the equivalent donors on the second ligand. It differs only from complex **1** and **2** by the stereoelectronic profile of the *N*-aromatic substituent.

The coordination geometry of related Mg(II) phenoxyimine complexes is also highly solvent dependent; for example, (^*^t^*Bu_2_,Ar^Mes^^L)_2_Mg, when crystallized in the presence of thf, forms a square pyramidal thf-adduct as reported by Chakraborty and co-workers.^14^ When the reaction of H^*^t^*Bu_2_,Dipp^L and [MgN″_2_]_2_ is carried out in pyridine, ligand redistribution results in a *pseudo*-octahedral complex, (^*^t^*Bu_2_,Ar^Dipp^^L)_2_Mg(py)_2_.^12^

The solid-state structures of **1** and **2** exhibit similar bond lengths and angles with respect to each other, demonstrating that the substituent effects are limited. The Mg–N and Mg–O bond lengths are shorter than those observed in reported *bis*(phenoxyimine) magnesium complexes (Mg–N = 2.0951(7)–2.3532(16) Å; Mg–O = 1.8959(6)–2.0028(13) Å),^7,12,17^ likely caused by the fact that these are 5- or 6-coordinate (where the coordination sphere is completed by a L ligand). Square planar (^*^t^*Bu_2_,Ar^Mes*^^L)_2_Mg has longer Mg–N bonds (2.1814(13) Å), a compensation to the increased electrostatic repulsion in this coordination geometry.^8^

Calcium complexes **3** and **4** demonstrate a distorted trigonal bipyramidal coordination geometry, with τ_5_ parameters of 0.66 and 0.72 for complexes **3** and **4** respectively (where τ_5_ = 0 corresponds to an idealized square based pyramid and τ_5_ = 1 corresponds to an idealized trigonal bipyramid)^18^ (Figure 1). The presence of a thf molecule in each system reflecting the increased ionic radius of Ca(II) compared to Mg(II).^19^ Unlike complexes **1** and **2**, the O and N donors of the two ligands in **3** and **4** are in a pseudo *trans* arrangement, in agreement with the solution NMR spectroscopic data which implies the two ligands are magnetically equivalent and related by a *C*_2_ symmetry element.

There is only a single example of a *bis*(phenoxyimine) calcium complex where the ligand does not contain pendent donors; six-coordinate [O-2-[{C(H)=N–C_6_F_5_}-6-^*t*^Bu-C_6_H_3_)]_2_Ca(thf)_2_ reported by Sarazin *et al*..^20^ Related complexes with pendent donor groups are six- or eight-coordinate dependent on the nature of the ligand.^6a,9e^ The Ca–O_L_ and Ca–N distances in **3** and **4** for are similar with respect to each other and at the lower end of the ranges for related complexes (Ca–O_L_ = 2.1822(15)–2.2640(15) Å and Ca–N = 2.4547(19)–2.5399(10) Å),^9e^ as expected when taking into account lower coordination number (Table 1).

### Consideration of Phenoxide Substitution Pattern and Synthetic Methodology; Accessing Heteroleptic Schiff-Base Alkaline Earth Complexes

The synthesis of **1**–**4** indicates that formation of homoleptic *bis*(ligand) alkaline earth complexes remains favorable across the ligand imine substitution patterns explored, when synthesis is carried out using metal silylamide starting materials.

In design of phenoxyimine ethylene polymerization catalysts of the form (L)Ni(py)(Me), Jian, Mecking and co-workers have explored the varying steric profiles of ligands;^10,21^ for example, incorporating a bulky anthracenyl substituent in the 6-position of the phenolic ring and varying the imine substituent.^10^ Comparison of percentage buried volumes (*V*_bur_)^22^ for these complexes reflected that bulky imine groups (such as Ar*) with free rotation preferentially orient away from the metal center (*V*_bur_ = 48%) but incorporation of dibenzosuberyl substituents resulted in slightly more effective shielding of both apical coordination positions of the metal cation (*V*_bur_ = 52%).

**Scheme 2:**
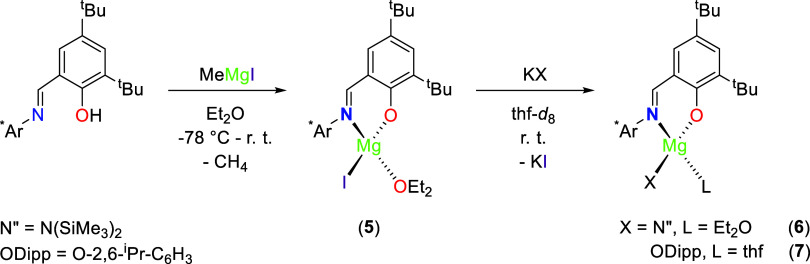
Protonolysis Reactions between H^*^t^*Bu_2_,Ar*^L and MeMgI Affording (^*^t^*Bu_2_,Ar*^L)MgI(OEt_2_) (**5**), and Subsequent Salt Metathesis Reactions with KX to Form (^*^t^*Bu_2_,Ar*^L)MgN″(OEt_2_) (**6**) and (^*^t^*Bu_2_,Ar*^L)Mg(ODipp)(thf) (**7**)

With this in mind, an investigation of kinetic stabilization provided through use of increasingly large substituents at both the phenolic ring and the imine would undoubtedly ultimately result in isolable heteroleptic Mg and Ca phenoxyimine complexes using the same protonolysis route; this approach has proven effective in numerous metal and ligand combinations across the periodic table to stabilize reactive complexes.^23^ However, an alternate synthetic approach guided by the work of Ma *et al*. was instead considered.^8^ Heteroleptic complex (^*^t^*Bu_2_,Ar*^L)MgI(OEt_2_) (**5**) was synthesized in a straightforward reaction of equimolar amounts of H^*^t^*Bu_2_,Ar*^L and MeMgI in diethyl ether, and was isolated as a yellow powder in 91% yield (Scheme 2). Solution NMR spectroscopic data was in agreement with Ma’s analogue and consistent with a *C*_1_ molecular symmetry. While it is tempting to only focus on ligand steric bulk, this demonstrates that the importance of the synthetic protocol when accessing heteroleptic species cannot be underestimated.

Furthermore, no ligand redistribution was observed in subsequent salt metathesis reactions. Complex **5** was shown to readily react with KN” and KODipp in thf affording (^*^t^*Bu_2_,Ar*^L)MgN″(OEt_2_) (**6**) and (^*^t^*Bu_2_,Ar*^L)Mg(ODipp)(thf) (**7**) in 39 and 47% yields, respectively. (Scheme 2).

The ^1^H NMR spectra of **6** and **7** demonstrate all the expected resonances for ^*^t^*Bu_2_,Ar*^L in a complex of molecular *C*_1_ symmetry. In complex **6**, a broad singlet at 0.07 ppm, which integrates to 18H, represents the N(Si*Me*_3_)_2_ protons (Figure S23). In complex **7**, a triplet at 6.49 ppm with a ^3^*J*_H–H_ coupling constant of 7.5 Hz, integrating to 1H, represents the O-4-C_6_*H*_3_ proton of the aryloxide initiating group; the adjacent O-3,5-C_6_*H*_3_ protons were assigned using the HMBC spectrum as part of the overlapping multiplet at 6.86 ppm (Figure S27). The O-2,6-CH*Me*_2_-C_6_H_3_ methyl protons appear as a doublet at 1.03 ppm with ^3^*J*_H–H_ coupling constant of 6.9 Hz; the septet at 3.31 ppm indicates the mutually coupled O-2,6–C*H*Me_2_-C_6_H_3_ methine protons of the *iso*-propyl groups.

Only a small set of solid state structures of any heteroleptic Schiff-base magnesium complexes have previously been reported, all with silylamide ligands,^6a,9b,9f,12,24^ with the exception of the iodide complex (L)Mg(I)(Et_2_O) reported by Ma *et al*. (L = (O-2-{C(H)=N-2,6-CHPh_2_-4-^i^Pr–C_6_H_2_}-4,6-*^t^*Bu-C_6_H_2_)).^8^ Crystals of related the tetrahydrofuran adduct of **6**, (^*^t^*Bu_2_,Ar*^L)MgN″(thf), were grown at room temperature; the connectivity of the structure as distorted tetrahedral is clear but the data does not permit detailed discussion of the metrical parameters. Comparison with solid state structures of related complexes indicates the importance of the substituent in the 6-phenolic position; -H or -halide substitution results in dimeric systems with an Mg_2_O_2_ diamond core,^6a,9b,12^ in the presence of additional donor groups, whether from solvent or from the ligand.^6a,24^

**Figure 2:**
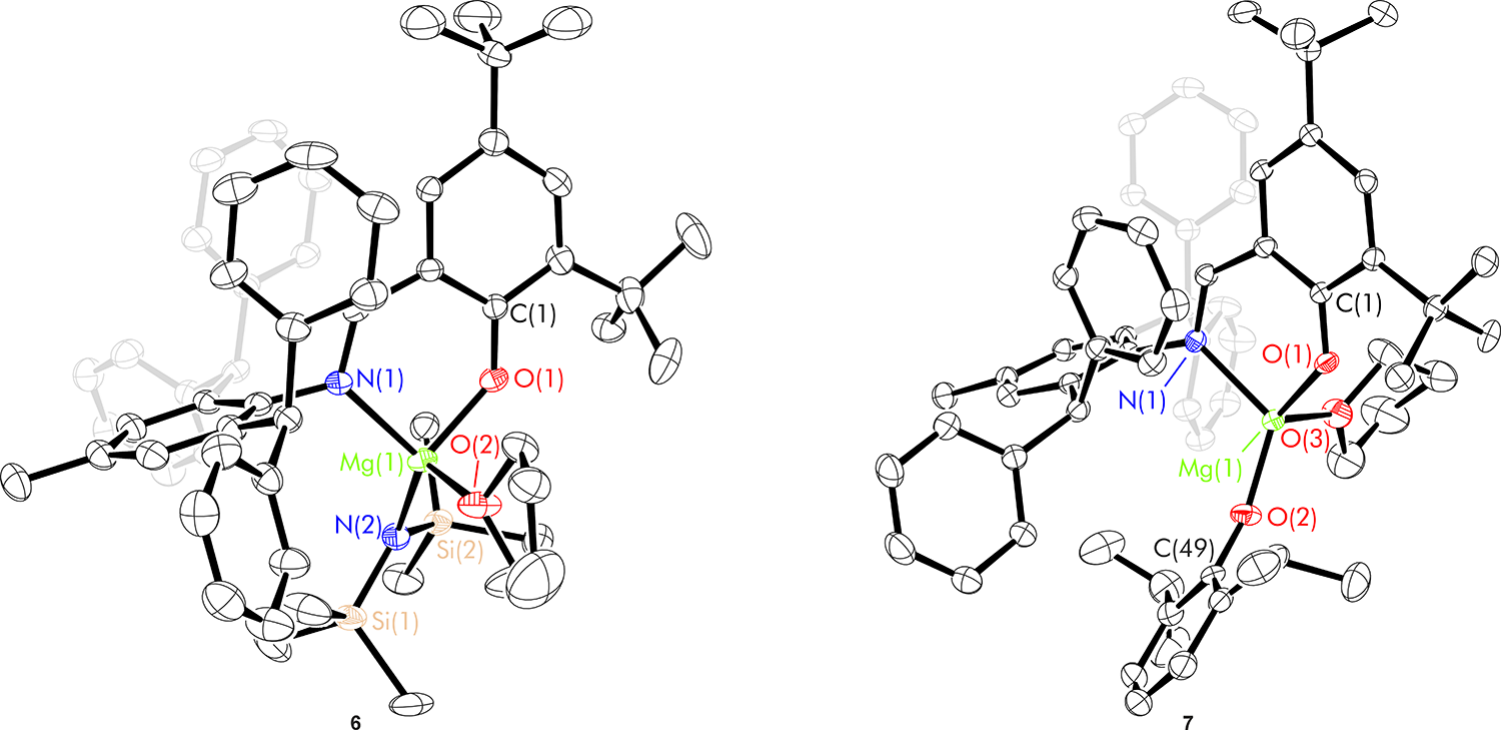
Thermal displacement ellipsoid drawings (30% probability) of (^*^t^*Bu_2_,Ar*^L)Mg(N″)(thf), (^*^t^*Bu_2_,Ar*^L)Mg(ODipp)(thf) (**7**). All hydrogen atoms have been omitted for clarity.

Single crystals of (^*^t^*Bu_2_,Ar*^L)Mg(ODipp)(thf) (**7**), suitable for an X-ray diffraction study, were similarly grown from a saturated diethyl ether solution at room temperature (Figure 2). In the solid state, the geometry around the magnesium is distorted tetrahedral (τ_4_ = 0.78).^15^ The Mg–O_ODipp_ bond length (1.822(3) Å) is shorter than the Mg–O_L_ bond lengths from the phenoxyimine ligands in both **7** and homoleptic complex **2** (1.891(3) Å and 1.8988(11)–1.9085(11) Å respectively), suggesting it may result in a relatively higher energy barrier to insertion reactions. The heteroleptic magnesium iodide complex with a similar ligand, reported by Ma *et al*. has a more regular tetrahedral geometry (τ_4_ = 0.92).^8^ The N–Mg–O_L_ bond angles are close to 90° in both structures, but the X–Mg-O_L_/N_L_ (where X = O_ODipp_ or I) angles are significantly larger in complex **7**; the larger steric profile of the aryloxide ligand results in larger bond angles and a less regular geometry to minimize electrostatic repulsions.

### Small 4,6-Phenoxide Substituents; Schiff-Base Alkaline Earth Multimetallic Clusters

In order to investigate the nature of accessible multimetallic structural motifs in this system, equimolar protonolysis reactions of H^Dipp^L and alkaline earth silylamides (CaN″_2_(thf)_2_ (N″ = N(SiMe_3_)_2_)) and SrN_2_^″^(thf)_2_ were initially carried out in toluene. They were found to form multimetallic clusters, with the major isolable products being (^H_2_,Dipp^L)_3_Ca_2_(N″)(thf) (**8**) and (^H_2_,Dipp^L)_6_Sr_3_ (**9**), both with μ_2_-O bridges (*vide infra*). Rational synthesis could be afforded by utilization of the appropriate M/L ratio (2:3 and 1:2 for Ca and Sr respectively). Reaction between H^Dipp^L and BaN″_2_(thf)_3_ resulted in a highly insoluble powder which precluded further characterization.

For calcium complex **8**, single crystals suitable for an X-ray diffraction study were grown from toluene and confirmed the connectivity in the solid-state as (^H_2_,Dipp^L)_3_Ca_2_(N″)(thf). When crystals were grown from diethyl ether, crystals with the same core structure but substitutional disorder of diethyl ether and thf were obtained. There are no comparable asymmetric group 2 bimetallic structures with a phenoxyimine supporting ligand; Mosquera, Cano and co-workers have reported a related heterobimetallic complex (^H_2_,Dipp^L)_3_KAlMe, which featured a tripodal aluminate metalloligand.^25^ Ca(1) is in a distorted trigonal prismatic coordination environment (CaN_3_O_3_), bound to the three phenoxyimine ligands. The three phenoxide O atoms bridge to Ca(2); the six Ca–O distances are all *ca*. 2.3 Å, with the longest distances being those bonds directly opposing the silylamide and thf groups (Ca(2)–O(1) = 2.352(2) Å, Ca(2)–O(2) = 2.375(2) Å and Ca(2)–O(3) = 2.308(2) Å). Ca(2) is in a highly distorted square-based pyramidal environment, also bound to the silylamide N(4) and thf O(4). Despite the asymmetry in the solid-state, the ^1^H NMR spectrum of the reaction mixture indicated the presence of a single calcium-bound phenoxyimine ligand environment, bound thf and silylamide ligand indicated by a resonance at 0.31 ppm (Figures S33–S37).

For strontium complex **9**, the ^1^H NMR spectrum of the reaction mixture showed a single ligand environment, HN″ by-product, and absence of strontium-bound silylamide ligand consistent with two ligands per Sr metal center. A singlet representing the imine *H*C=N proton was observed at 7.67 ppm. Two distinct multiplets at 7.28 and 6.51 ppm represent the 3-C_6_*H*_4_ and 6-C_6_*H*_4_ protons, respectively. The remaining aromatic protons (N-3,4,5-C_6_*H*_3_ and 4,5-C_6_*H*_4_) feature as part of an overlapping multiplet in the region 6.82–7.11 ppm which integrates to 5H. In solution, restricted rotation of the Dipp groups is observed by the splitting out of the signals associated with the methine and methyl protons. Two septets with ^3^*J*_H–H_ = 6.7 Hz are observed at 3.14 and 2.05 ppm which represent the methine C*H*Me_2_ protons. Four doublets in the range 0.91–0.44 ppm, each integrating to 3H with ^3^*J*_H–H_ = 6.8 Hz signify the four methyl CH*Me*_2_ groups.

**Figure 3:**
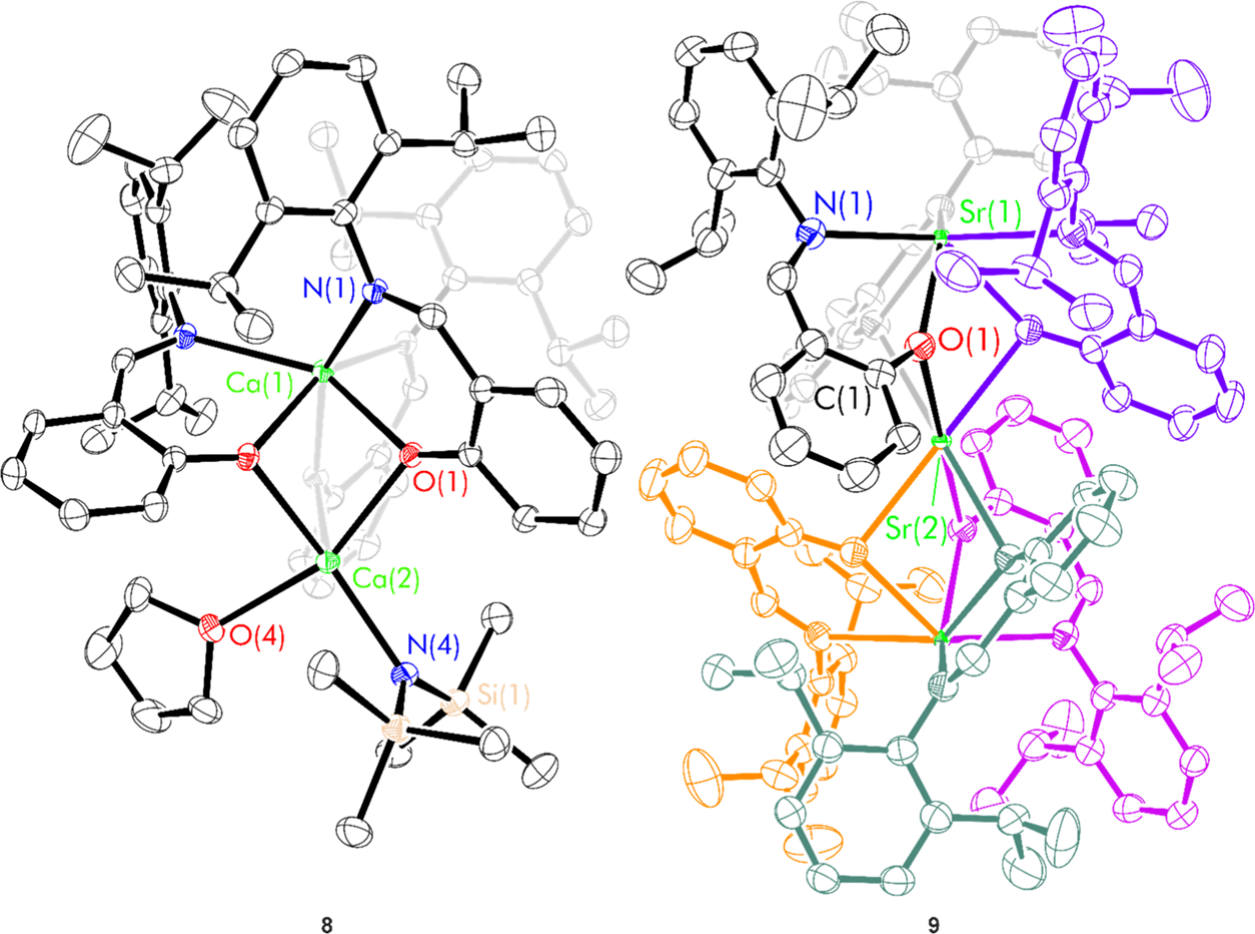
Thermal displacement ellipsoid drawings (30% probability) of (^H_2_,Dipp^L)_3_Ca_2_N″(thf) (**8**) and (^H_2_,Dipp^L)_6_Sr_3_ (**9**). All hydrogen atoms have been omitted and each ligand in complex **10** has been color-coded for additional clarity.

**Scheme 3:**
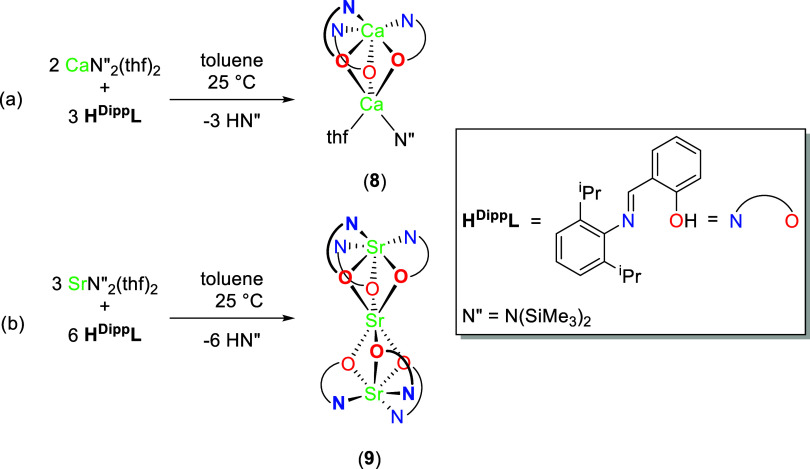
Protonolysis Reactions between H^Dipp^L and MN″_2_(thf)_2_ (M = Ca and Sr) to Afford Clusters (a) (^H_2_,Dipp^L)_3_Ca_2_(N″)(thf) (**8**) and (b) Sr_3_(^H_2_,Dipp^L)_6_ (**9**)

Single crystals suitable for an X-ray diffraction study were obtained by the slow evaporation of a saturated thf solution; this allowed the assignment of the trimetallic structure Sr_3_(^H_2_,Dipp^L)_6_ (**9**) in the solid state (Scheme 3b and Figure 3). Rational synthesis of complex **9** was achieved using a ^H_2_,Dipp^L/Sr ratio of 2:1 and was isolated as a colorless powder in a 65% yield. The solid-state structure of complex **9** is the same regardless of the solvent of crystallization, featuring a trimeric motif in which the three Sr centers are bridged through ligand phenoxide oxygen atoms in a μ_2_-fashion to create a paddlewheel structure. Two different Sr^2+^ environments are present; a central Sr^2+^ which part of a SrO_6_ unit and terminal Sr^2+^ which is part of a SrO_3_N_3_ unit. There is only one ligand environment (relatable via a *C*_3_ rotation axis which runs through both Sr environments and an inversion center at the central Sr) which is consistent with the solution ^1^H NMR spectrum.

The central Sr(2) atom is in a trigonal prismatic coordination environment, bound to six oxygen atoms of distinct ligands (O(1)–Sr(2)–O(1) = 68.25(12)°). The six coordinate terminal Sr(1) environment is ligated by both the N and O donors from only three ligands (MN_3_O_3_). The three Sr(1)-N bonds lie in a trigonal planar arrangement, with N–Sr(1)-N angles of 119.24(3)°. The O–Sr(1)-O bond angle is much smaller (73.70°) rendering the overall geometry around this metal center to be a highly distorted octahedron.

This relatively unusual trimetallic motif has previously been observed in Mg_3_(^H_2_,Ph^L)_6_, a magnesium complex with a smaller phenoxyimine ligand.^11^ Here, the structure is of lower symmetry with respect to **9** with a single molecule in the asymmetric unit; the central Mg is again in a trigonal prismatic coordination environment but the terminal Mg are much closer to a regular octahedral geometry. There are limited examples of a Sr_3_(μ_2_-E)_6_ core and the bridging atom was O in each case.^26^ More generally, this motif has also been reported for divalent metal complexes of Mg,^27^ Ca,^4a,28^ Ba,^26d,29^ Yb^30^ and Eu,^31^ with further examples of large clusters and extended structures highlighting the vast potential of structural diversity available.

## Conclusions and Outlook

Variation of both the steric bulk of the *N*-imine substituents and the Ae metal ion were found to have no effect on protonolysis reactions between [MgN″_2_]_2_ or CaN″_2_(thf)_2_ (N″ = N(SiMe_3_)_2_) and H^*^t^*Bu_2_,Ar^L (1-OH-2-CH = NAr-4,6-*^t^*Bu-C_6_H_2_; Ar = 2,6-^i^Pr–C_6_H_3_ = Dipp or 2,6-CHPh_2_-4-Me-C_6_H_2_ = Ar*) regardless of reaction stoichiometry, with homoleptic *bis*(ligand) complexes (^*^t^*Bu_2_,Dipp^L)_2_Mg (**1**), (^*^t^*Bu_2_,Ar*^L)_2_Mg (**2**), (^*^t^*Bu_2_,Dipp^L)_2_Ca(thf) (**3**) and (^*^t^*Bu_2_,Ar*^L)_2_Ca(thf) (**4**) isolated.

Straightforward isolation of heteroleptic complex (^*^t^*Bu_2_,Ar*^L)MgI(OEt_2_) (**5**) was achieved from the reaction of equimolar amounts of H^*^t^*Bu_2_,Ar*^L and MeMgI. Futhermore, no subsequent ligand redistribution was observed when complex **5** readily reacted with KN″ or KODipp to form (^*^t^*Bu_2_,Ar*^L)MgN″(OEt_2_) (**6**) and (^*^t^*Bu_2_,Ar*^L)Mg(ODipp)(thf) (**7**). These results highlight that, though well thought out ligand design is fundamental to the isolation of many reactive complexes, the importance of reaction protocol must not be overlooked and should be considered carefully. Finally, when considering ligands with the smallest phenoxide substituents, (H^H_2_,Dipp^L), multimetallic clusters were afforded; (^H_2_,Dipp^L)_3_Ca_2_(N″)(thf) (**8**) and (^H_2_,Dipp^L)_6_Sr_3_ (**9**).

These bidentate ligands have enabled the relatively facile isolation of well-defined Ae complexes and therefore offer some advantages to the NOON and NON ligands. They can be used to highlight the difficulties of isolating often desirable heteroleptic species in such a simple system with homoleptic, heteroleptic and multimetallic identified. While these simple phenoxyimine ligands lack the provision of a kinetically stabilized binding pocket for the Ae metal that ubiquitous ligands such as β-diketiminates, (hetero)scorpionates or *bis*(imino)carbazolate offer, we find that it is not always necessary when appropriate synthetic conditions are utilized.

In light of our previous studies of phenoxyimine-supported alkaline earth complexes for the ring-opening polymerization of cyclic esters, we continue to explore this area with the diverse selection of complexes to understand the reactivity of complexes **1**–**8** further.

## Experimental Section

### Safety Statement

Benzene is an irritant, highly flammable liquid, may cause genetic defects, may cause cancer or cause damage to organs by repeated exposure. As such, it should be handled with care and appropriate safety precautions.

#### (^*^t^*Bu_2_,Dipp^L)_2_Mg (**1**)

A solution of H^*^t^*Bu_2_,Dipp^L (1.00 g, 2.55 mmol) in benzene (5 mL) was added to a solution of [MgN″_2_]_2_ (0.448 g, 0.650 mmol) in benzene (5 mL). The resultant yellow solution was stirred for 3 h at room temperature and the volatiles were removed *in vacuo*. The crude product was triturated with pentane (∼5 mL) to afford (^*^t^*Bu_2_,Dipp^L)_2_Mg (**1**) as a yellow powder (1.03 g, 69%). Single crystals of **1**, suitable for an X-ray diffraction study, were obtained by the slow evaporation of a saturated room temperature benzene solution. ^**1**^**H NMR** (benzene-*d*_6_, 400 MHz, 298 K): δ 7.91 (s, 2H, ***H***C=N), 7.71 (d, 2H, ^4^*J*_H–H_ = 2.7 Hz, 5-C_6_***H***_2_), 7.01 (m, 4H, N-3,5-C_6_***H***_3_), 6.92 (dd, 2H, *J*_H–H_ = 6.1, 3.2 Hz, N-4-C_6_***H***_3_), 6.85 (d, 2H, ^4^*J*_H–H_ = 2.7 Hz, 3-C_6_***H***_2_), 3.69 (sept, ^3^*J*_H–H_ = 6.8 Hz, 2H, C***H***Me_2_), 2.62 (sept, ^3^*J*_H–H_ = 6.8 Hz, 2H, C***H***Me_2_), 1.65 (s, 18H, 6-C***Me***_3_-C_6_H_2_), 1.24 (s, 18H, 4-C***Me***_3_-C_6_H_2_), 1.22–0.39 (d, ^3^*J*_H–H_ = 6.8 Hz, 24H, CH***Me***_2_) ppm. ^**13**^**C{^1^H} NMR** (benzene-*d*_6_, 101 MHz, 298 K): δ 177.21 (H***C***=N), 169.37 (1-***C***_6_H_2_), 147.66 (N-1-***C***_6_H_3_), 141.28 (6-***C***_6_H_2_), 135.55 (4-***C***_6_H_2_), 131.24 (5-***C***_6_H_2_), 130.19 (3-***C***_6_H_2_), 126.38 (N-3,5-***C***_6_H_3_), 124.56 (N-4-***C***_6_H_3_), 123.60 (N-2,6-***C***_6_H_3_), 119.00 (2-***C***_6_H_2_), 35.50 (4-***C***Me_3_-C_6_H_2_), 33.64 (6-***C***Me_3_-C_6_H_2_), 31.17 (4-C***Me***_3_-C_6_H_2_), 29.70 (6-C***Me***_3_-C_6_H_2_), 28.42 (***C***HMe_2_), 24.67–22.65 (CH***Me***_2_) ppm. Anal. Calcd. for C_54_H_76_MgN_2_O_2_: C, 80.12; H, 9.46; N, 3.46. Found: C, 80.67; H, 9.82; N, 3.40.

#### (^*^t^*Bu_2_,Ar*^L)_2_Mg (**2**)

A solution of pro-ligand H^*^t^*Bu_2_,Ar*^L (0.750 g, 1.14 mmol) in benzene (5 mL) was added to a solution of [MgN″_2_]_2_ (0.197 g, 0.286 mmol) in benzene (5 mL). The resultant yellow solution was stirred for 3 h at room temperature and volatiles were removed *in vacuo*. The crude product was triturated with pentane (∼5 mL) to afford (^*^t^*Bu_2_,Ar*^L)_2_Mg (**2**) as a yellow powder (0.539 g, 71%). Single crystals of **2** suitable for an X-ray diffraction study, were obtained by the slow evaporation of a saturated room temperature benzene solution. ^**1**^**H NMR** (benzene-*d*_6_, 400 MHz, 298 K): δ 7.46–6.52 (overlapping m, 44H, N-3,5-C_6_***H***_2_, CH(3,4,5-C_6_***H***_5_)_2_), 7.38 (d, ^4^*J*_H–H_ = 2.7 Hz, 2H, 5-C_6_***H***_2_), 6.26 (s, 2H, ***H***C=N), 5.61 (s, 4H, C***H***(C_6_H_5_)_2_), 5.42 (d, ^4^*J*_H–H_ = 2.6 Hz, 2H, 3-C_6_***H***_2_), 1.87 (s, 6H, 4-***Me***-C_6_H_2_), l.35 (s, 18H, 6-C***Me***_3_-C_6_H_2_), 1.16 (s, 18H, 4-C***Me***_3_-C_6_H_2_) ppm. ^**13**^**C{**^**1**^**H} NMR** (benzene-*d*_6_, 101 MHz, 298 K): δ 180.42 (H***C***=N), 168.50 (1-***C***_6_H_2_), 147.74 (N-1-***C***_6_H_2_), 143.73–126.05 (N-2,3,4,5,6-***C***_6_H_2_ and CH(2,3,4,5,6-***C***_6_H_5_)_2_), 142.35 (CH(1-***C***_6_H_5_)_2_), 138.22 (2-***C***_6_H_2_), 134.01 (4-***C***_6_H_2_), 130.73 (5-***C***_6_H_2_), 130.11 (3-***C***_6_H_2_), 119.14 (2-***C***_6_H_2_), 53.66 (***C***H(C_6_H_5_)_2_), 35.30 (6-***C***Me_3_-C_6_H_2_), 33.40 (4-C_6_H_2_-***C***Me_3_), 31.26 (4-C***Me***_3_-C_6_H_2_), 29.52 (6-C***Me***_3_-C_6_H_2_), 21.12 (4-***Me***-C_6_H_2_) ppm. Anal. Calcd. for C_96_H_96_MgN_2_O_2_: C, 86.43; H, 7.25; N, 2.10. Found: C, 86.07; H, 7.29; N, 2.10.

#### (^*^t^*Bu_2_,Dipp^L)_2_Ca(thf) (**3**)

A solution of pro-ligand H^*^t^*Bu_2_,Dipp^L (0.800 g, 2.03 mmol) in benzene (5 mL) was added dropwise to a solution of CaN″_2_(thf)_2_ (0.508 g, 1.02 mmol) in benzene (5 mL). The resultant yellow solution was then stirred for 3 h at room temperature before volatiles were removed *in vacuo*. The crude product was then triturated with pentane (∼5 mL) to afford (^*^t^*Bu_2_,Dipp^L)_2_Ca(thf) (**3**) as a yellow powder (0.91 g, 46%). Single crystals of **3**, suitable for an X-ray diffraction study, were obtained by slow evaporation of a saturated room temperature benzene solution. ^**1**^**H NMR** (benzene-*d*_6_, 400 MHz, 298 K): δ 7.98 (s, 2H, ***H***C=N), 7.60 (d, 2H, ^4^*J*_H–H_ = 2.7 Hz, 3-C_6_***H***_2_), 7.14 (overlapping m, 6H, N-3,4,5-C_6_***H***_3_), 6.97 (d, 2H, ^4^*J*_H–H_ = 2.7 Hz, 5-C_6_***H***_2_), 3.59 (m, 4H, OC***H***_2_), 3.12 (sept, ^3^*J*_H–H_ = 6.9 Hz, 4H, C***H***Me_2_), 1.31 (s, 18H, 6-C_6_H_2_–C***Me***_3_), 1.25 (s, 18H, 4-C_6_H_2_–C***Me***_3_), 1.23 (m, 4H, OC***H***_2_), 1.16 (d, ^3^*J*_H–H_ = 6.8 Hz, 24 H, CH***Me***_2_) ppm. ^**13**^**C{**^**1**^**H} NMR** (benzene-*d*_6_, 101 MHz, 298 K): δ 173.80 (1-***C***_6_H_2_), 168.96 (H***C***=N), 150.32 (N-1-***C***_6_H_3_), 140.10 (6-***C***_6_H_2_), 140.03 (N-2,6-***C***_6_H_3_), 133.49 (4-***C***_6_H_2_), 130.80 (5-***C***_6_H_2_), 129.61 (3-***C***_6_H_2_), 125.58 (N-4-***C***_6_H_3_), 123.98 (N-3,5-***C***_6_H_3_), 121.29 (2-***C***_6_H_2_), 69.02 (OO***C***H_2_), 35.30 (4-C_6_H_2_-***C***Me_3_), 33.70 (6-C_6_H_2_-***C***Me_3_), 31.51 (4-C_6_H_2_–C***Me***_3_), 29.76 (6-C_6_H_2_–C***Me***_3_), 28.73 (***C***HMe_2_), 24.89 (CH***Me***_2_), 24.78 (O***C***H_2_) ppm. Anal. Calcd. for C_58_H_84_CaN_2_O_3_: C, 77.63; H, 9.44; N, 3.12. Found: C, 77.24; H, 9.45; N, 3.00.

#### (^*^t^*Bu_2_,Ar*^L)_2_Ca(thf) (**4**)

A solution of pro-ligand H^*^t^*Bu_2_,Ar*^L (0.300 g, 0.457 mmol) in benzene (5 mL) was added to a solution of CaN″_2_(thf)_2_ (0.115 g, 0.229 mmol) in benzene (5 mL). The resultant yellow solution was stirred for 3 h at room temperature and volatiles were removed *in vacuo*. The crude product was triturated with pentane (∼5 mL) to afford (^*^t^*Bu_2_,Ar*^L)_2_Ca(thf) (**4**) as a yellow powder (0.240 g, 74%). Single crystals of **4**, suitable for an X-ray diffraction study, were obtained by the slow evaporation of a saturated room temperature benzene solution. ^**1**^**H NMR** (benzene-*d*_6_, 400 MHz, 298 K): δ 7.56 (d, ^4^*J*_H–H_ = 2.8 Hz, 2H, 5-C_6_***H***_2_), 6.93–6.62 (overlapping m, 44H, CH(3,4,5-C_6_***H***_5_)_2,_ N-3,5-C_6_***H***_2_), 6.26 (s, 2H, ***H***C=N), 5.70 (s, 4H, C***H***(C_6_H_5_)_2_), 5.60 (d, ^4^*J*_H–H_ = 2.8 Hz, 2H, 3-C_6_***H***_2_), 3.95 (broad s, 4H, OC***H***_2_), 1.81 (s, 6H, 4-***Me***-C_6_H_2_), 1.39 (s, 18H, 6-C***Me***_3_-C_6_H_2_), 1.33 (s, 18H, 4-C***Me***_3_-C_6_H_2_) 1.06 (broad s, 4H, OC***H***_2_) ppm. ^**13**^**C{**^**1**^**H} NMR** (benzene-*d*_6_, 101 MHz, 298 K): δ 174.73 (H***C***=N), 166.68 (1-***C***_6_H_2_), 147.71 (N-1-***C***_6_H_2_), 139.47 (CH(1-***C***_6_H_5_)_2_), 136.65 (6-***C***_6_H_2_), 135.17–123.93 (N-2,3,4,5,6-***C***_6_H_2,_ CH(2,3,4,5,6-***C***_6_H_5_)_2_), 131.65 (4-***C***_6_H_2_), 130.24 (3-***C***_6_H_2_), 119.61 (2-***C***_6_H_2_), 67.59 (O***C***H_2_), 51.56 (***C***H(C_6_H_5_)_2_), 33.43 (6-***C***Me_3_-C_6_H_2_), 31.47 (4-***C***Me_3_-C_6_H_2_), 29.58 (4-C***Me***_3_-C_6_H_2_), 27.63 (4-C***Me***_3_-C_6_H_2_), 22.43 (O***C***H_2_), 18.78 (4-***Me***-C_6_H_2_) ppm. Anal. Calcd. for C_100_H_104_CaN_2_O_3_: C, 84.46; H, 7.37; N, 1.97. Found: C, 83.78; H, 7.53; N, 2.00.

#### (^*^t^*Bu_2_,Ar*^L)MgI(OEt_2_) (**5**)

To a solution of pro-ligand H^*^t^*Bu_2_,Ar*^L (3.00 g, 4.57 mmol) was added MeMgI (1.68 mL, 5.03 mmol; 3.0 M in Et_2_O) at −78 °C. The resulting suspension was allowed to warm to room temperature and stirred for 16 h. The volatiles were removed *in vacuo* to afford a yellow powder which was washed with pentane and dried once more to yield (^*^t^*Bu_2_,Ar*^L)MgI(OEt_2_) (**5**) as a yellow powder (3.92 g, 98%). ^**1**^**H NMR** (thf-*d*_8_, 101 MHz, 298 K): δ 7.38 (overlapping m, 4H, CH(2,3,4,5,6-C_6_***H***_5_)_2_), 7.23 (d, ^4^*J*_H–H_ = 2.7 Hz, 1H, 3- or 5-C_6_***H***_2_), 7.14 (overlapping m, 6H, CH(2,3,4,5,6-C_6_***H***_5_)_2_), 6.89 (overlapping m, 10H, CH(2,3,4,5,6-C_6_***H***_5_)_2_), 6.63 (s, 2H, N-3,5-C_6_***H***_2_), 6.12 (s, 1H, ***H***C=N), 5.54 (s, 2H, C***H***(C_6_H_5_)_2_), 5.28 (d, ^4^*J*_H–H_ = 2.7 Hz, 1H, 3- or 5-C_6_***H***_2_), 3.38 (q, 4H, ^3^*J*_H–H_ = 7.0 Hz, OC***H***_**2**_CH_3_), 2.12 (s, 3H, 4-C***H***_3_-C_6_H_2_), 1.47 1.12 (s, 9H each, 4,6-C***Me***_3_-C_6_H_2_), 1.12 (m, 6H, OCH_2_C***H***_**3**_) ppm. Complex **5** was then directly utilized for subsequent salt-metathesis reactions.

#### (^*^t^*Bu_2_,Ar*^L)MgN″(OEt_2_) (**6**)

A solution of KN″ (0.0113 g, 0.0568 mmol) in thf-*d*_8_ (0.25 mL) was added to a solution of (^*^t^*Bu_2_,Ar*^L)MgI(OEt_2_) (**5**) (0.0500 g, 0.0568 mmol) in thf-*d*_8_ (0.25 mL) in an NMR tube. The reaction mixture was filtered and volatiles were removed *in vacuo* from the filtrate to yield (^^t^Bu_2_,Ar*^L)MgN″(OEt_2_) (**6**) as a yellow-green powder (0.020 g, 39%). Single crystals of (^^t^Bu_2_,Ar*^L)MgN″(thf), suitable for an X-ray diffraction study, were obtained by the slow evaporation of a saturated room temperature diethyl ether solution of **6** where trace thf was still present. ^**1**^**H NMR** (thf-*d*_8_, 400 MHz, 298 K): δ 7.26 (d, ^4^*J*_H–H_ = 2.7 Hz, 1H, 3-C_6_***H***_2_), 7.22 (m, 8H, CH(2,6-C_6_***H***_5_)_2_), 6.98 (m, 8H, CH(3,5-C_6_***H***_5_)_2_), 6.80 (m, 4H, CH(4-C_6_***H***_5_)_2_), 6.71 (s, 2H, N-3,5-C_6_***H***_2_), 5.87 (s, 1H, ***H***C=N), 5.57 (s, 2H, C***H***(C_6_H_5_)_2_), 5.05 (d, ^4^*J*_H–H_ = 2.7 Hz, 1H, 5-C_6_***H***_2_), 3.38 (q, 4H, ^3^*J*_H–H_ = 7.0 Hz, OC***H***_2_CH_3_), 2.14 (s, 3H, 4-C***H***_3_-C_6_H_2_), 1.45, (s, 9H, 4-C***Me***_3_-C_6_H_2_), 1.11 (overlapping m, 15H, OCH_2_C***H***_3_, 6-C***Me***_3_-C_6_H_2_), 0.07 (broad s, 18H, N(Si***Me***_3_)_2_) ppm. ^**13**^**C{**^**1**^**H} NMR** (thf-*d*_8_, 101 MHz, 298 K): δ 178.16 (1-***C***_6_H_2_), 167.36 (H***C***=N), 145.33 (N-1-***C***_6_H_2_), 144.03 (CH(1-***C***_6_H_5_)_2_), 138.90 (4-***C***_6_H_2_), 134.05 (N-4-***C***_6_H_2_), 133.10 (6-***C***_6_H_2_), 131.06 (3-***C***_6_H_2_), 130.29 (5-***C***_6_H_2_), 129.60 (N-2,6-***C***_6_H_2_), 128.90 (CH(3,5-***C***_6_H_5_)_2_), 127.90 (CH(4-***C***_6_H_5_)_2_), 127.81 (N-3,5-***C***_6_H_2_), 126.16 (CH(2,6-***C***_6_H_5_)_2_), 118.09 (2-***C***_6_H_2_), 65.34 (O***C***H_2_CH_3_), 52.42 (N-2,6-***C***H(C_6_H_5_)_2_), 34.98 (4-C_6_H_2_-***C***Me_3_), 33.17 (6-C_6_H_2_-***C***Me_3_), 30.82 (6-C***Me***_3_-C_6_H_2_), 29.37 (4-C***Me***_3_-C_6_H_2_), 20.47 (4-***Me***-C_6_H_2_), 14.71 (OCH_2_***C***H_3_), 5.35 (N(Si***Me***_3_)_2_) ppm. Anal. Calcd. for C_58_H_76_MgN_2_O_2_Si_2_: C, 76.16; H, 8.49; N, 3.06. Found: C, 76.04; H, 7.65; N, 2.40.

#### (^*^t^*Bu_2_,Ar*^L)Mg(ODipp)(thf) (**7**)

A solution of KO-2,6-^*i*^Pr–C_6_H_3_ (0.123 g, 0.568 mmol) in thf (7.5 mL) was added to a solution of (^*^t^*Bu_2_,Ar*^L)MgI(OEt_2_) (**5**) (0.500 g, 0.568 mmol) in thf-*d*_8_ (0.25 mL). The solution was stirred for 18 h before filtration and extraction using pentane (3 × 10 mL). Volatiles were removed *in vacuo* to afford (^*^t^*Bu_2_,Ar*^L)Mg(ODipp)(thf) (**7**) as a pale brown powder (0.250 g, 47%). Single crystals of **7**, suitable for an X-ray diffraction study, were obtained by the slow evaporation of a saturated room temperature diethyl ether solution. ^**1**^**H NMR** (thf-*d*_8_, 400 MHz, 298 K): δ 7.33 (d, ^4^*J*_H–H_ = 2.7 Hz, 1H, 5-C_6_***H***_2_), 7.06–6.85 (overlapping m, 20H, CH(2,3,4,5,6-C_6_***H***_5_)_2_), 6.86 (m, 2H, 3,5-C_6_***H***_3_), 6.68 (s, 1H, N-3,5-C_6_***H***_2_), 6.49 (t, 1H, ^3^*J*_H–H_ = 7.5 Hz), 6.12 (s, 1H, ***H***C=N), 5.55 (s, 2H, C***H***(C_6_H_5_)_2_), 5.43 (d, ^4^*J*_H–H_ = 2.7 Hz, 1H, 3-C_6_***H***_2_), 3.62 (m, 2H, OC***H***_2_), 3.31 (hept, ^3^*J*_H–H_ = 6.9 Hz, C***H***Me_2_), 2.21 (s, 3H, N-4-***Me***-C_6_H_2_) 1.77 (m, 2H, OC***H***_2_), 1.54 1.15 (s, 9H each, 4,6-C***Me***_3_-C_6_H_2_), 1.03 (d, 12H, ^3^*J*_H–H_ = 6.9 Hz, CH***Me***_2_) ppm. ^**13**^**C{**^**1**^**H} NMR** (thf-*d*_8_, 101 MHz, 298 K): δ 177.51 (H***C***=N), 167.38 (1-***C***_6_H_2_), 158.74 (1-***C***_6_H_3_), 146.10 (N-1-***C***_6_H_2_), 142.79 (CH(1-***C***_6_H_5_)_2_), 136.80 (2,6-***C***_6_H_3_), 134.81 (N-2,6-***C***_6_H_2_), 128.83–125.78 (N-3,4,5-***C***_6_H_2_, CH(2,3,4,5,6-***C***_6_H_5_)_2_, 3,4,5,6-***C***_6_H_2_), 122.85 (3,5-***C***_6_H_3_), 118.86 (2-***C***_6_H_2_), 113.81 (4-***C***_6_H_3_), 65.99 (O***C***H_2_), 52.98 (***C***H(C_6_H_5_)_2_), 35.27 and 33.35 (***C***Me_3_), 30.61 and 28.95 (C***Me***_3_), 26.78 (***C***HMe_2_) 23.86 (O***C***H_2_), 22.63 (CH***Me***_2_) ppm.

#### (^H_2_,Dipp^L)_3_Ca_2_(N″)(thf) (**8**)

CaN″_2_(thf)_2_ (1.79 g, 3.55 mmol) and proligand H^Dipp^L (1.00 g, 3.55 mmol), were each dissolved in toluene (15 mL) and cooled to −78 °C. The solution of H^Dipp^L was added dropwise to the solution of Ca{N(SiMe_3_)_2_}_2_(thf)_2_ over a period of 10 min. The reaction mixture was warmed to room temperature and stirred for 18 h. The resultant pale-yellow solid was dried *in vacuo* and washed with cold hexane (4 × 5 mL) yielding (^H_2_,Dipp^L)_3_Ca_2_(N″)(thf) (**8**) as a colorless powder (1.03 g, 50%). Single crystals of (^H_2_,Dipp^L)_3_Ca_2_(N″)(thf) (**8**), suitable for an X-ray diffraction study, were obtained by the slow evaporation of both a saturated diethyl ether solution and a saturated toluene solution at room temperature. ^**1**^**H NMR** (toluene-*d*_8_, 600 MHz, 298 K): δ 7.67 (s, 3H, ***H***C=N), 7.26–6.98 (overlapping m, 7H, *N*-3,5-C_6_***H***_3_ and C_6_H_4_). 6.75 (d, 3H, ^3^*J*_H–H_ = 7.4 Hz, *N*-3,5-C_6_***H***_3_), 6.50 (3H, t ^3^*J*_H–H_ = 7.4 Hz, *N*-4-C_6_***H***_3_), 3.71 (broad s, 6H, OC***H***_2_), 3.16 and 1.98 (hept, ^3^*J*_H–H_ = 6.7 Hz, 3H, C***H***Me_2_), 1.31 (broad s, 6H, OC***H***_2_), 0.85 0.83 (d, ^3^*J*_H–H_ = 6.7 Hz, 9H each, CH***Me***_2_), 0.50 (overlapping d, 18H, CH***Me***_2_), 0.34, (s, 18H, SiMe_3_) ppm. ^**13**^**C{^1^H} NMR** (toluene-*d*_8_, 151 MHz, 298 K): δ 175.55 (H***C***=N), 168.04 (N-1-***C***_6_H_3_), 152.44 (1-***C***_6_H_2_), 141.31 (2-***C***_6_H_4_), 140.48 (N-2,6-***C***_6_H_3_), 138.31 135.2 (*N*-3,5-***C***_6_H_3_), 129.19 126.29 124.86 123.54 121.93 (N-2,6-***C***_6_H_3_ or 3,4,5,6-***C***_6_H_4_), 115.65 (N-4-***C***_6_H_3_), 69.57 (O***C***H_2_), 28.51, 28.35 (***C***HMe_2_), 26.05, 25.50 (CH***Me***_2_), 24.87 (O***C***H_2_), 23.37 21.44 (CH***Me***_2_), 24.48 (O***C***H_2_) 6.02 (Si***Me***_3_) ppm. Anal. Calcd. for C_67_H_92_Ca_2_N_4_O_4_Si_2_: C, 69.75; H, 8.04; N, 4.86. Found: C, 68.24; H, 8.32; N, 4.12 (Best result, crystals grown from Et_2_O).

#### (^H_2_,Dipp^L)_6_Sr_3_ (**9**)

SrN″_2_(thf)_2_ (0.0196 g, 0.0355 mmol) and proligand H^Dipp^L (0.0200 g, 0.0710 mmol) were each dissolved in toluene-*d*_8_ (∼0.25 mL) and combined in a Youngs tap NMR tube. The solution was dried *in vacuo*, yielding (^H_2_,Dipp^L)_6_Sr_3_ (**9**) as a colorless powder (0.015 g, 65%). Single crystals of **9** suitable for an X-ray diffraction study could either obtained by the slow evaporation of either a saturated thf solution or a saturated toluene solution at room temperature. ^**1**^**H NMR** (toluene-*d*_8_, 400 MHz, 298 K): δ 7.67 (s, 1H, ***H***C=N), 7.28 and 6.51 (m, 2H, 3,6-C_6_***H***_4_), 7.11–6.82 (overlapping m, 5H, N-3,4,5-C_6_*H*_3_ and 4,5-C_6_***H***_4_), 3.14 and 2.05 (hept, ^3^*J*_H–H_ = 6.7 Hz, 2H, C***H***Me_2_), 0.91–0.44 (d, ^3^*J*_H–H_ = 6.7 Hz, 12H, CH***Me***_2_) ppm.

For complexes **6** and **8** elemental analysis indicates a greater difference than ±0.4% between the calculated and measured carbon values. All analyses were carried out on spectroscopically pure, recrystallized samples at least twice. This is a phenomenon in alkaline earth chemistry for which there is precedent.^32^

## Supporting Information Available

General experimental considerations, additional synthetic details, representative NMR spectra, crystallographic data tables (PDF)
