# Supplementary material for: Exploring the Nuclearity and Structural Motifs of Phenoxyimine Alkaline Earth Complexes
Source: Organometallics. 2025 Mar 3;44(6):737–48. doi: 10.1021/acs.organomet.4c00504 (PMC11938394; doi:10.1021/acs.organomet.4c00504)
Supplement: Supplementary file 1 — om4c00504_si_001.pdf [file om4c00504_si_001.pdf]

Exploring the nuclearity and structural motifs of phenoxyimine alkaline earth complexes.

Amy V. Rizzo, Rebecca L. Jones, Matthew D. Haynes, Clement G. Collins Rice, Jean-Charles Buffet, Zoë R. Turner* and Dermot O'Hare*

Chemistry Research Laboratory, Department of Chemistry, University of Oxford, Mansfield Road, Oxford, OX1 3TA, United Kingdom. Tel: +44(0) 1865 285157; E-mail: zoe.turner@earth.ox.ac.uk and dermot.ohare@chem.ox.ac.uk.

Table of Contents

I.	General considerations	S3
II.	Representative NMR spectra	S4
III.	Additional synthetic details and characterising data	S24
IV.	Crystallographic data	S26
V.	Density functional theory	S33
VI.	References	S34

I. General considerations

All manipulations were carried out using standard Schlenk line or drybox techniques under an atmosphere of dinitrogen or argon. Protio solvents were degassed by sparging with dinitrogen, dried by passing through a column of activated sieves using an MBraun SPS-800 solvent purification system (pentane, hexane, toluene, benzene) and stored over potassium mirrors, or distilled from sodium metal (thf) and stored over activated 4 Å molecular sieves, or distilled from sodium-potassium alloy (diethyl ether) and stored over a potassium mirror. Deuterated solvents were dried over potassium (C_6D_6, C_7D_8) or CaH_2 (C_4D_8O, C_5D_5N, $CDCl_3$), distilled under reduced pressure and freeze-pump-thaw degassed three times prior to use.

Solution ^1H NMR spectra were recorded at 298 K, unless otherwise stated, on Bruker AVIII 400 nanobay or Bruker AVIII 500 or Bruker NEO 600spectrometers and $^{13}C\{^1H\}$ or ^{13}C spectra on the same spectrometers at operating frequencies of 100 and 125 MHz respectively. Two dimensional ^1H-^1H and ^{13}C-^1H correlation experiments were used, when necessary, to confirm ^1H and ^{13}C assignments. All NMR spectra were referenced internally to residual protio solvent (^1H) or solvent (^{13}C) resonances and are reported relative to tetramethylsilane (δ = 0 ppm). Chemical shifts are quoted in δ (ppm) and coupling constants in Hertz. Air sensitive samples were prepared in a glovebox under an inert atmosphere, using dried deuterated solvents in J. Young's NMR tubes.

Fourier transform infrared (FTIR) spectroscopy samples were prepared in a glove box as pellets using anhydrous potassium bromide (KBr). IR spectra were recorded on a Nicolet iS5 ThermoScientific spectrometer (range = 4000–400 cm^{-1} with resolution = 1 cm^{-1}) in transmission mode. A background spectrum was run prior to the sample and was subtracted from the sample spectra.

Samples were prepared in a glove box under a nitrogen atmosphere and sealed in glass tubes. Samples were run as electron impact (EI) mass spectra on an Agilent GC-TOF-MS by Dr James Wickens or Dr Victor Mikhailov (University of Oxford).

Elemental analyses (CHN) were carried out in duplicate at London Metropolitan University by Miss Orla McCullough.

p-Toluenesulfonic acid, di-*n*-butyl magnesium (1.0 M in heptane), 2,6-diisopropylphenol, methylmagnesium iodide (3.0 M in diethyl ether), zinc chloride, formic acid, calcium iodide, 4-methyl aniline, diphenyl methanol and 2-hydroxybenzaldehyde were all purchased from Sigma Aldrich and used as received. 3,5-Di-*tert*-butylsalicyladehyde (Fluorochem), 4-di-methylaminopyridine (Fluorochem), and 2,6-diisopropylaniline (Apollo Scientific) were all used as received. Potassium hydride (Sigma Aldrich) was purchased as a 30 wt% dispersion in mineral oil. Any excess oil was decanted away before the solid was washed with portions of diethyl ether until a free-flowing powder was obtained; this was dried under reduced pressure before use.

Pro-ligand $H^{tBu_2,Dipp}L$ (2-{(*E*)-2,6-diisopropyl phenylimino-methylene}-4,6-di-*tert*-butyl-phenol; 1-OH-2-{C(H)=N-2,6-^{i}Pr-C_6H_3}-4,6-^{t}Bu-C_6H_2; Dipp = 2,6-^{i}Pr-C_6H_3) was synthesised using a method adapted from Garcia-Valle *et al.* (Scheme S1a).[1] $Ca\{N(SiMe_3)_2\}_2(thf)_2$[2] and 2,6-diisopropyl potassium phenoxide[3] were adapted from literature procedures. Pro-ligand $H^{Dipp}L$,[4] potassium bis(trisilylmethyl)amide (KN''),[5] $Sr\{N(SiMe_3)_2\}_2(thf)_2$,[6] and $Ba\{N(SiMe_3)_2\}_2(thf)_3$[6] were all synthesised according to reported procedures.

II. **Representative NMR spectra**

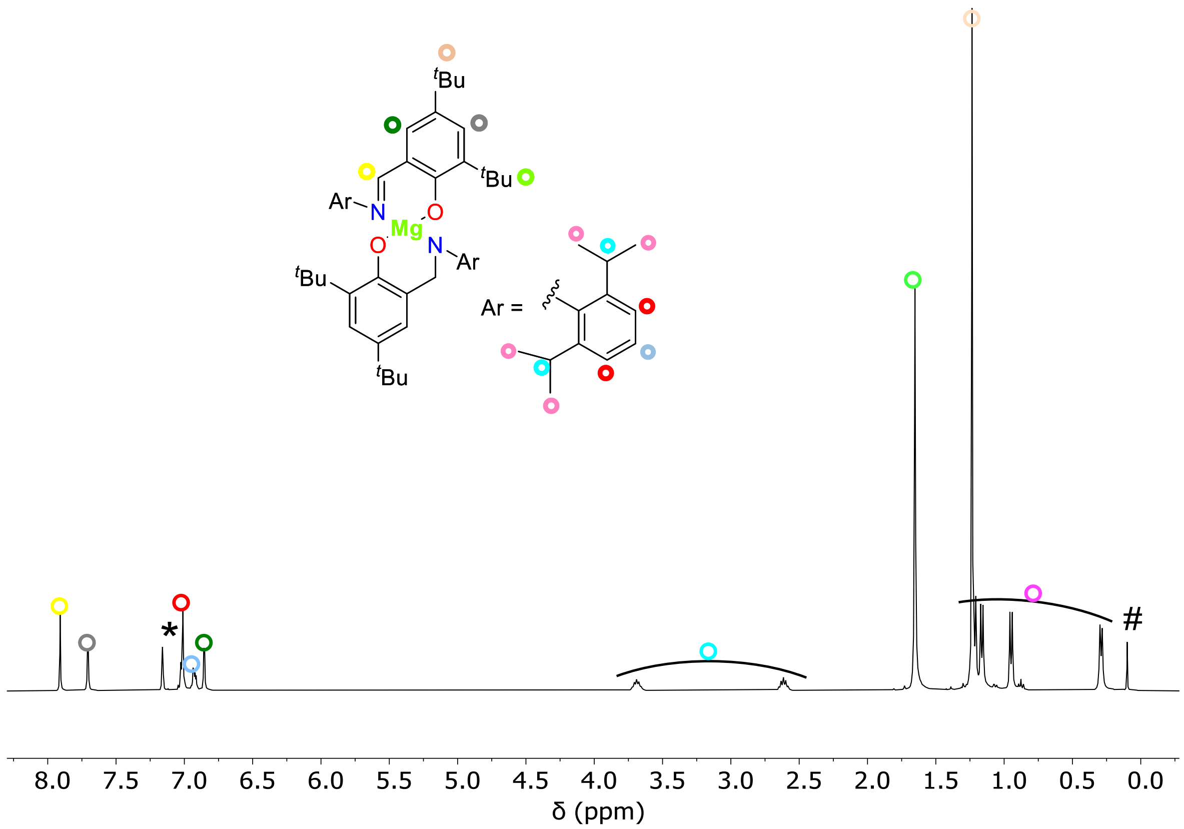

Figure S1. ^{1}H NMR spectrum (*C_6D_6, 400 MHz, 298 K) of (^{tBu_2,Dipp}L)$_2$Mg (**1**). # represents residual HN".

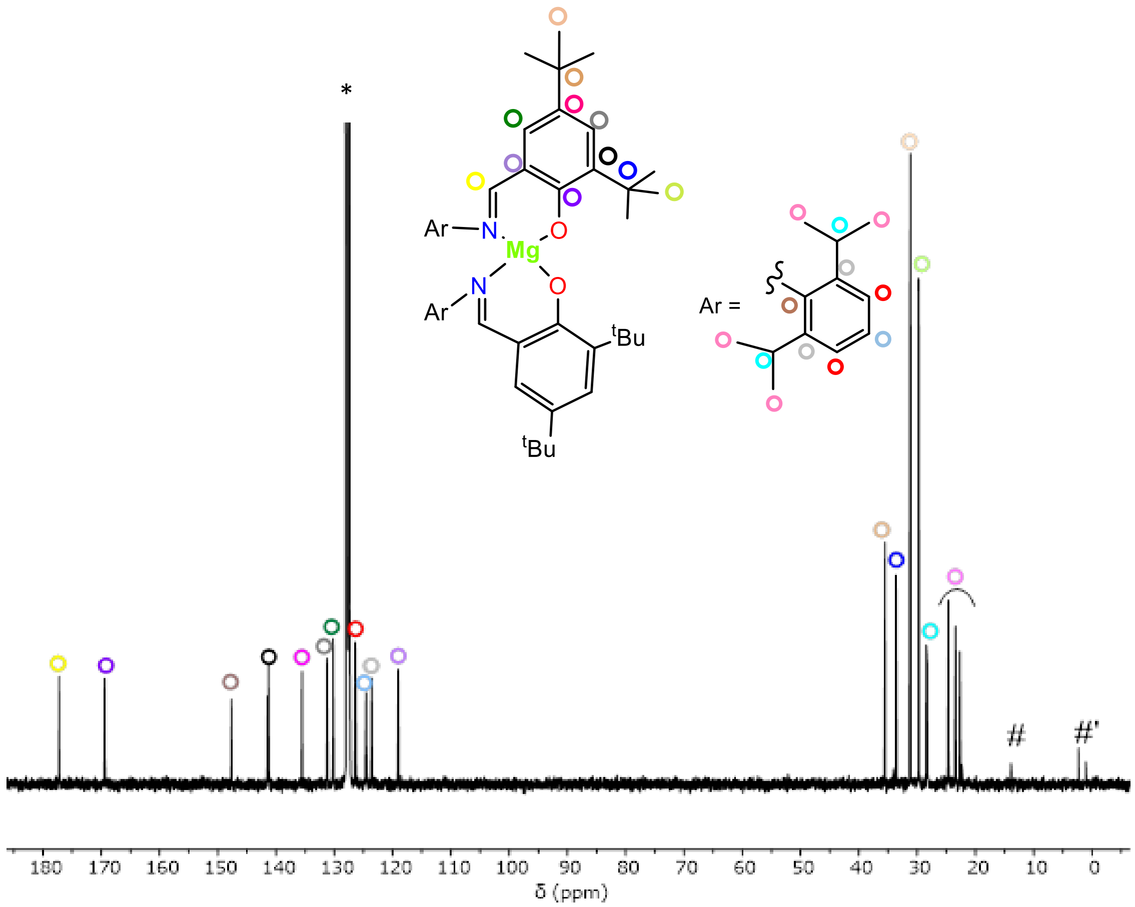

Figure S2. $^{13}C\{^{1}H\}$ NMR spectrum (*C_6D_6, 101 MHz, 298 K) of $(^{tBu_2,Dipp}L)_2Mg$ (**1**). # represents residual protio solvent (pentane), and #′ HN''.

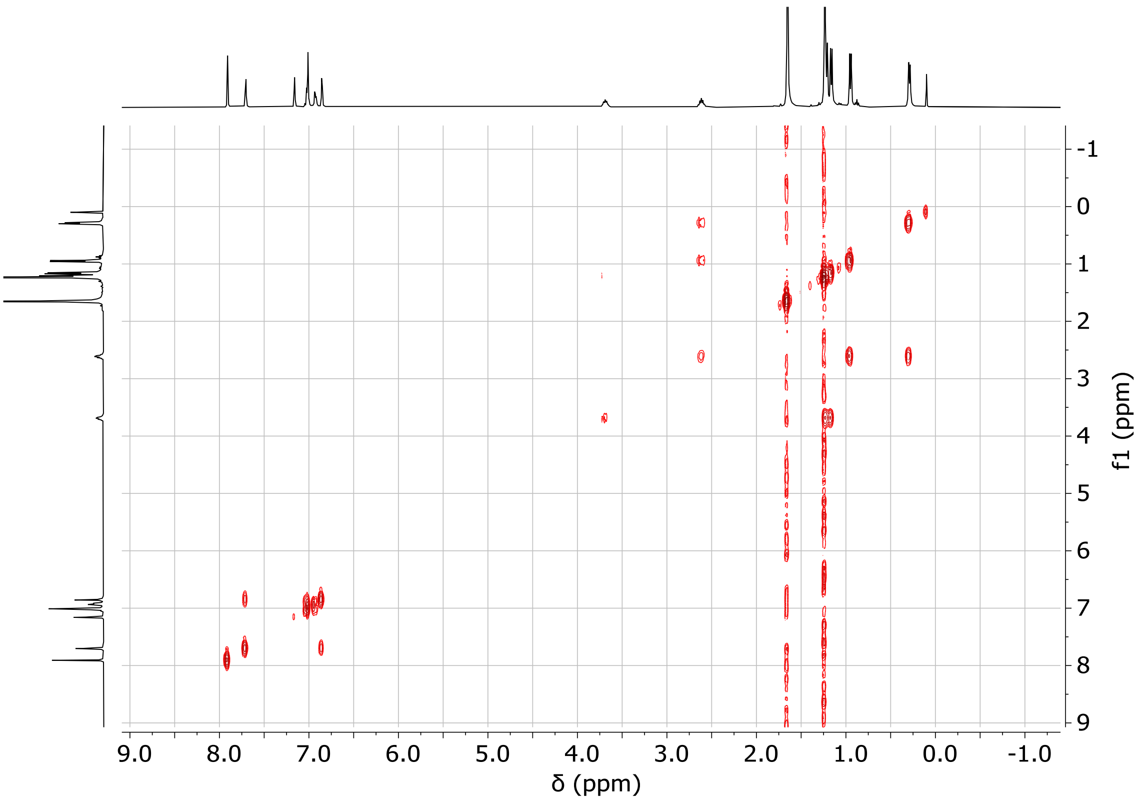

Figure S3. ^{1}H-^{1}H COSY NMR spectrum (C_6D_6, 400 MHz, 298 K) of $(^{tBu_2,Dipp}L)_2Mg$ (**1**).

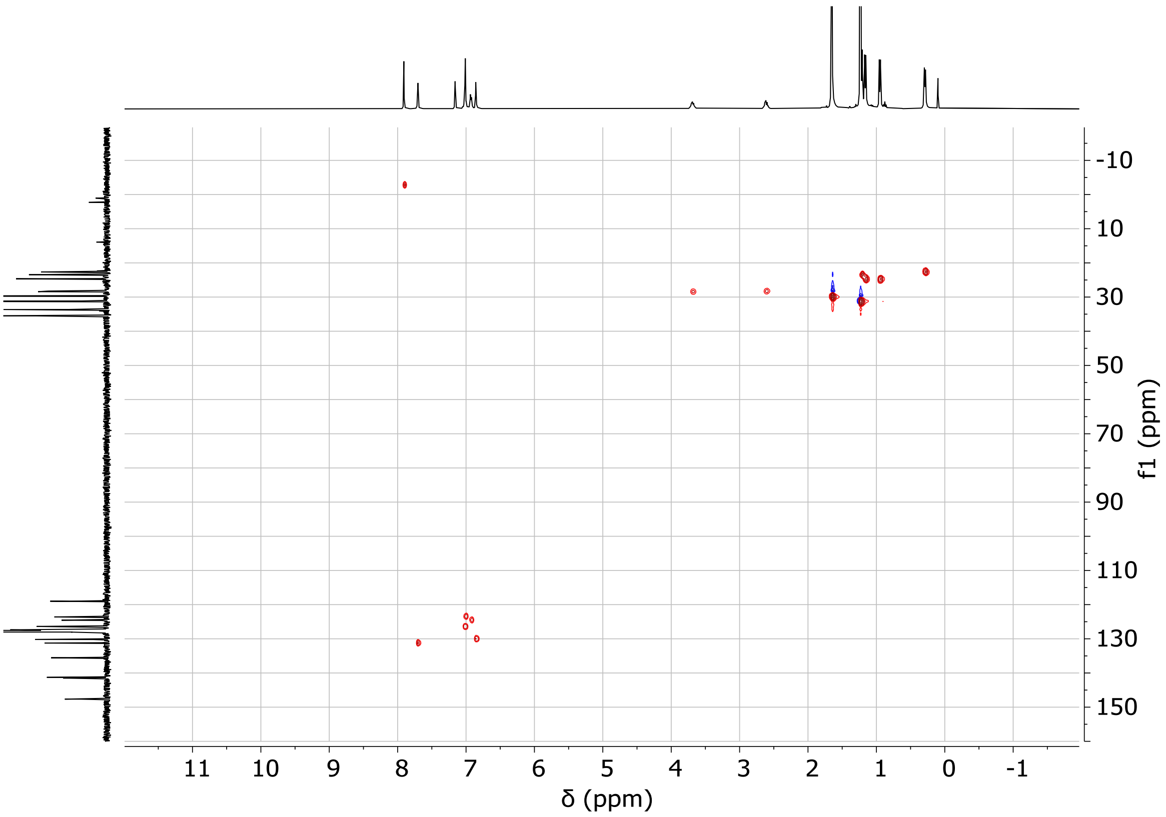

Figure S4. ^{1}H-^{13}C HSQC NMR spectrum (C_6D_6, 298 K) of $(^{tBu_2,Dipp}L)_2Mg$ (**1**).

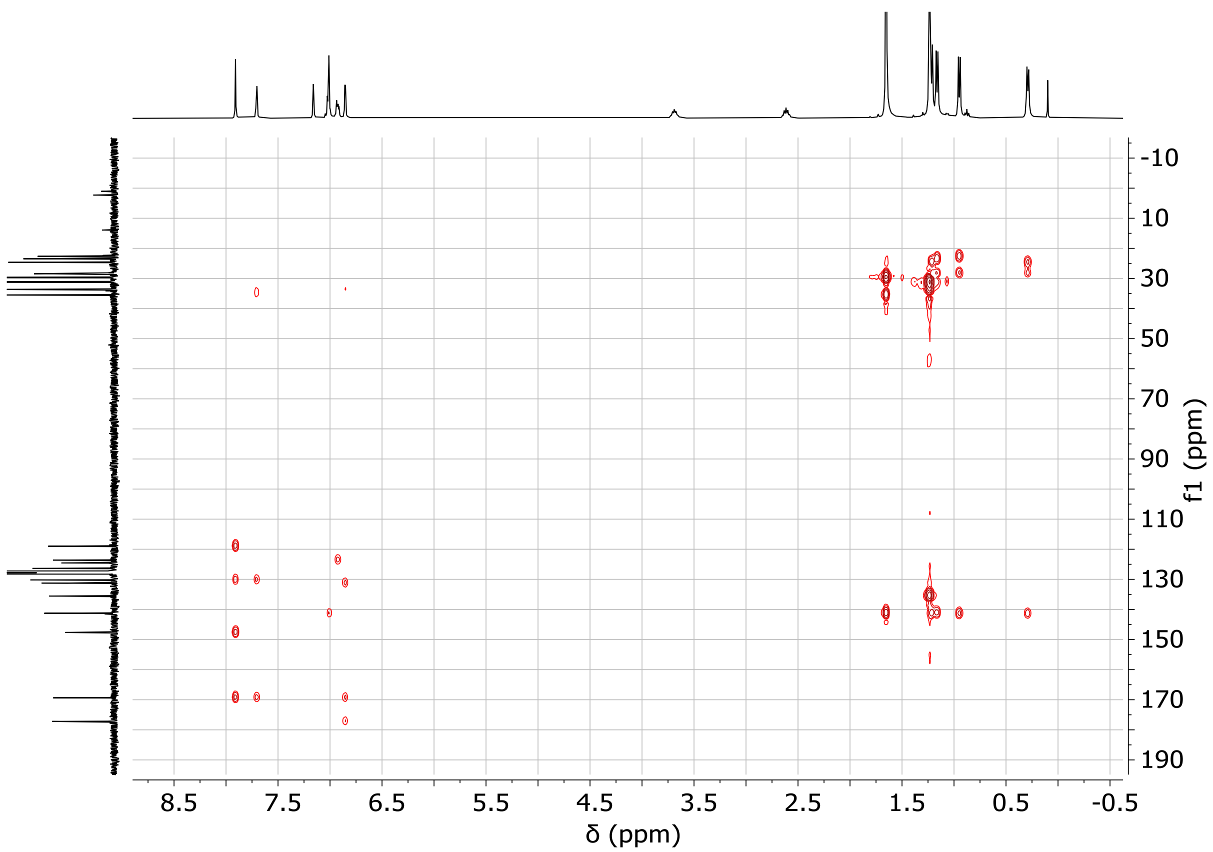

Figure S5. ^{1}H-^{13}C HMBC NMR spectrum (C_6D_6, 298 K) of $(^{tBu2,Dipp}L)_2Mg$ (**1**).

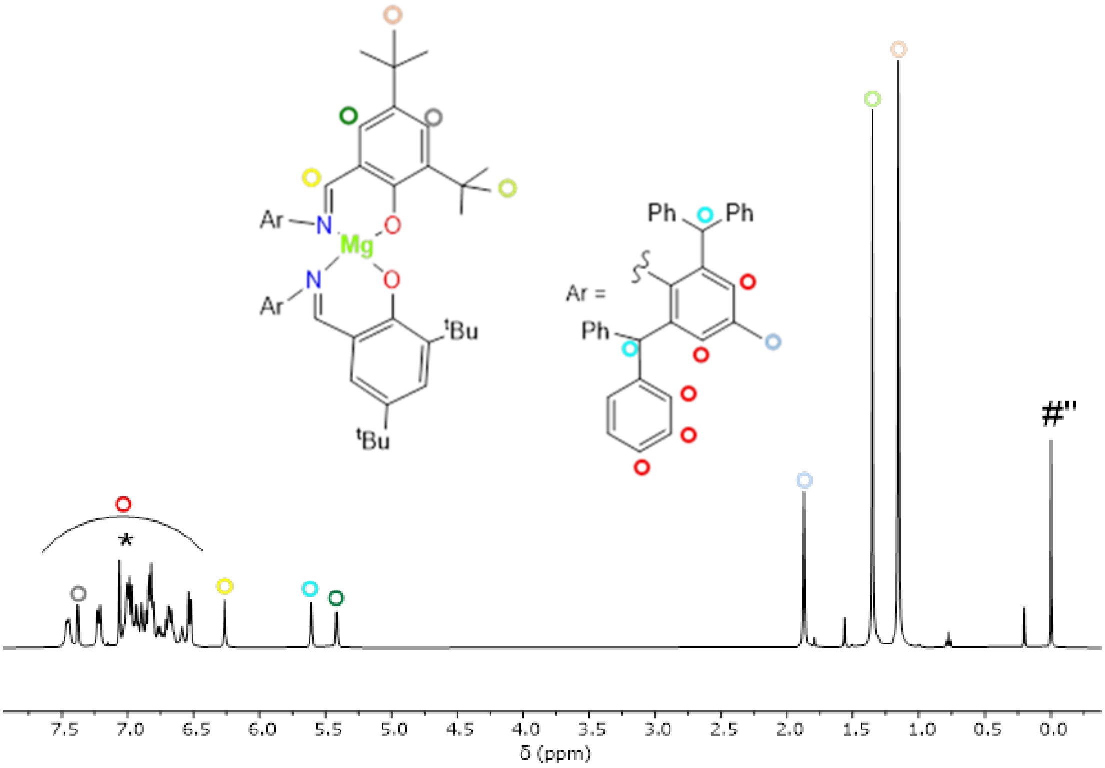

Figure S6. ^{1}H NMR spectrum (*C_6D_6, 400 MHz, 298 K) of ($^{tBu_2,Ar^*}$L)$_2$Mg (**2**). #" represents residual HN".

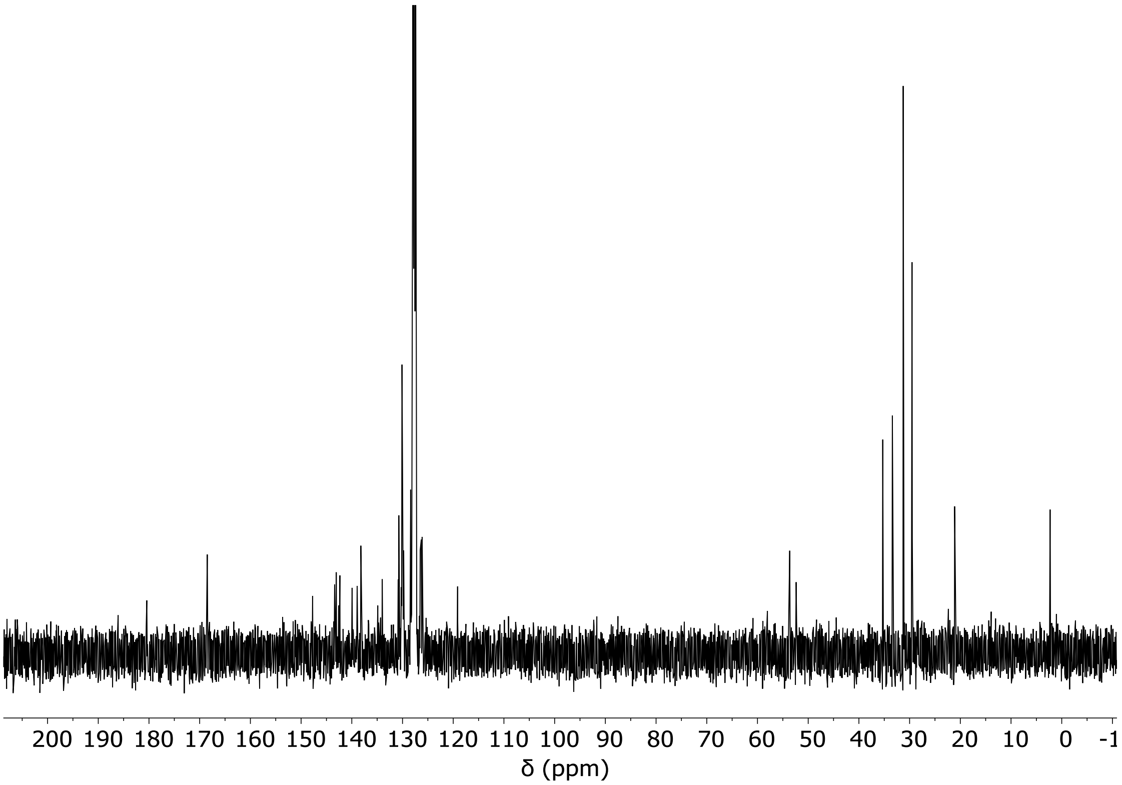

Figure S7. ^{13}C{^{1}H} NMR spectrum (*C_6D_6, 101 MHz, 298 K) of ($^{tBu_2,Ar^*}$L)$_2$Mg (**2**).

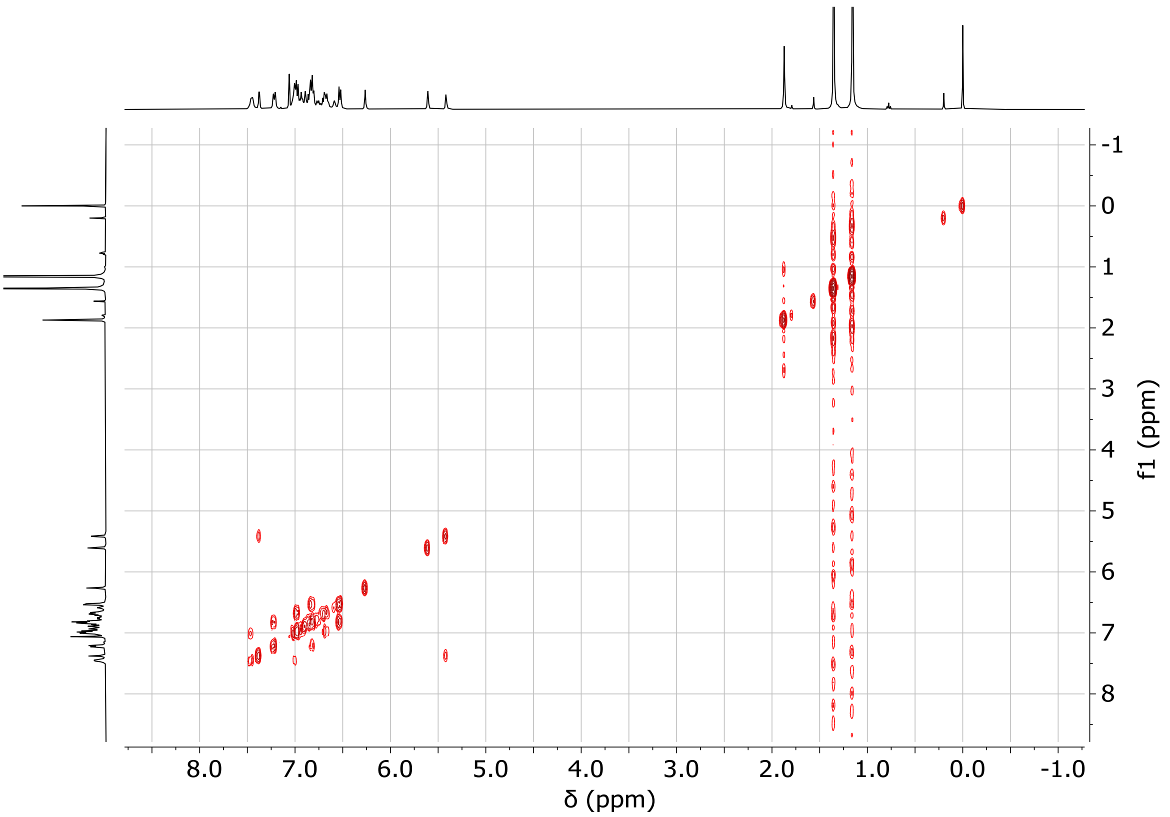

Figure S8. ^{1}H-^{1}H COSY NMR spectrum (C_6D_6, 400 MHz, 298 K) of ($^{tBu_2,Ar^*}L)_2Mg$ (**2**).

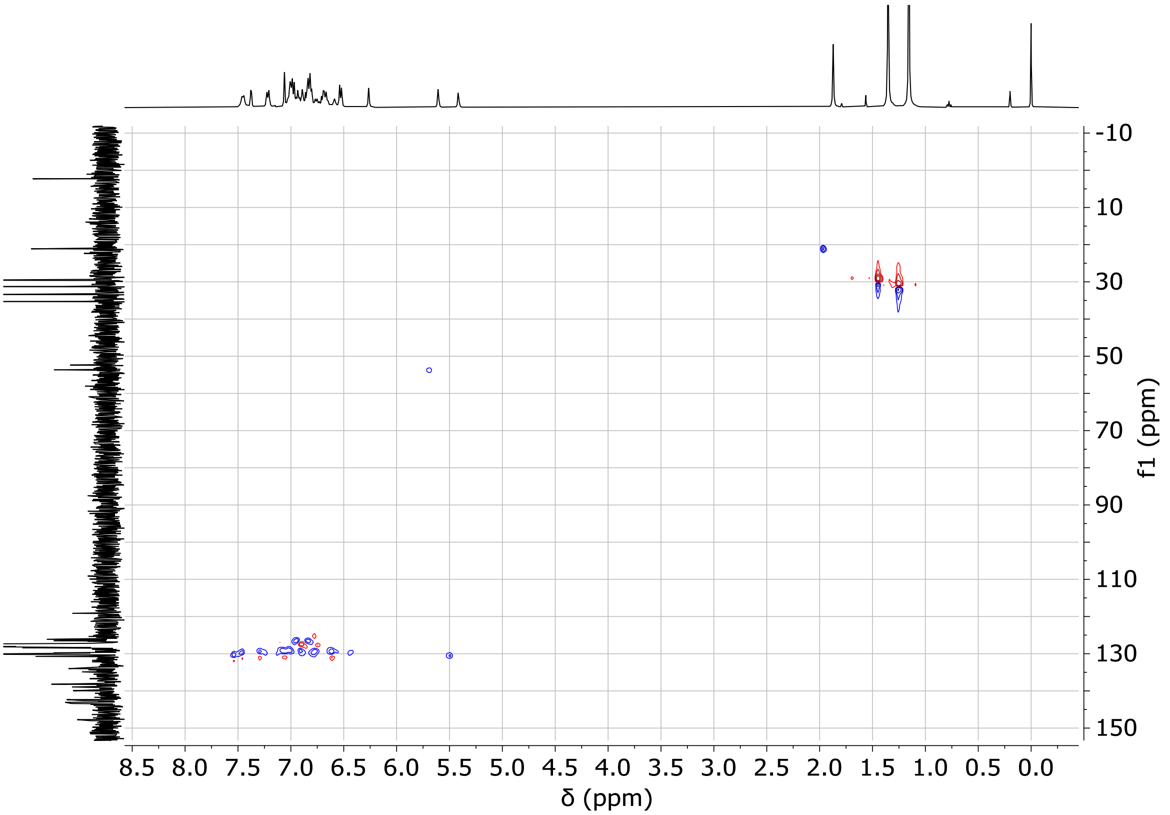

Figure S9. ^{1}H-^{13}C HSQC NMR spectrum (C_6D_6, 298 K) of ($^{tBu_2,Ar^*}L)_2Mg$ (**2**).

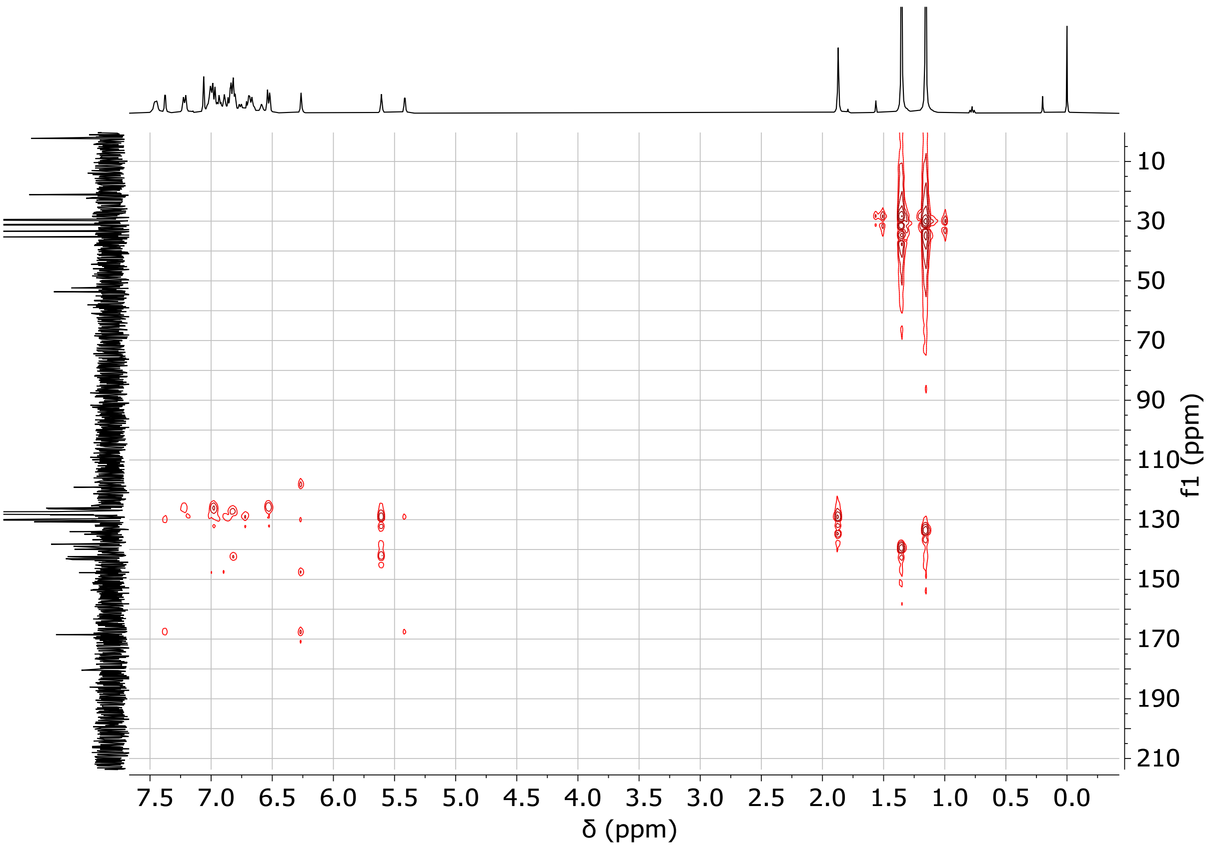

Figure S10. ^{1}H-^{13}C HMBC NMR spectrum (C_6D_6, 298 K) of ($^{tBu_2,Ar^*}L)_2Mg$ (**2**).

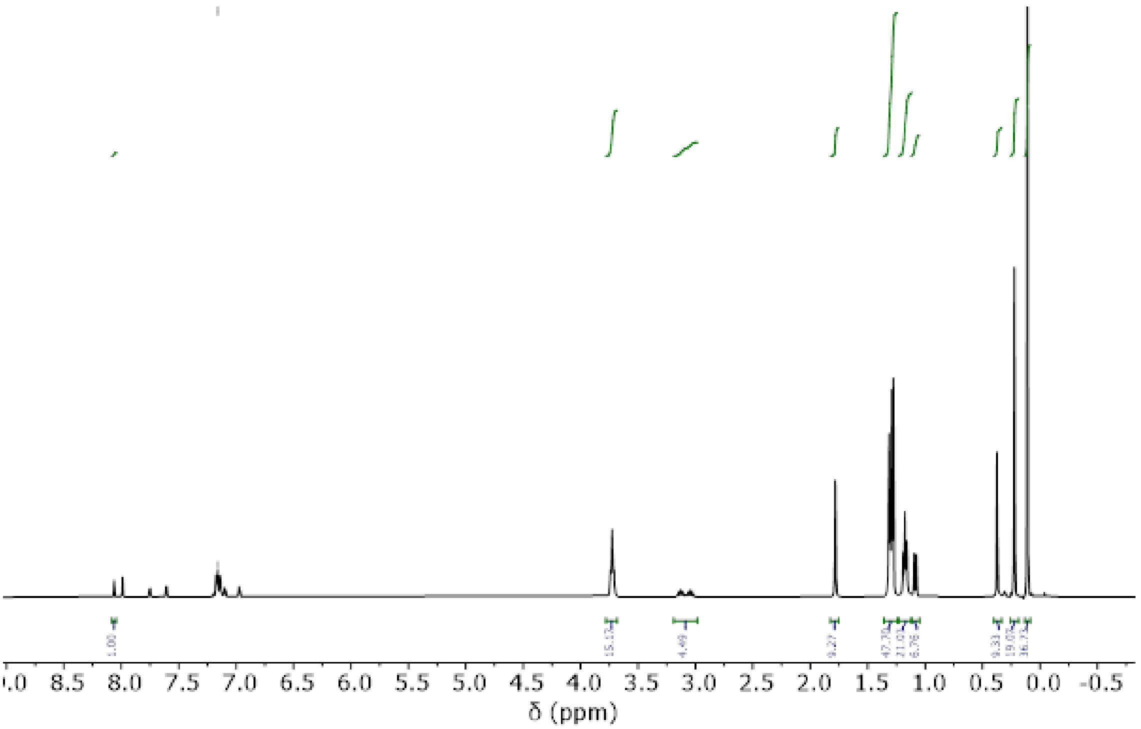

Figure S11. ^{1}H NMR spectrum (*C_6D_6, 400 MHz, 298 K) of the 1:1 reaction between H^{tBu_2,Dipp}L and CaN"$_2$(thf)$_2$.

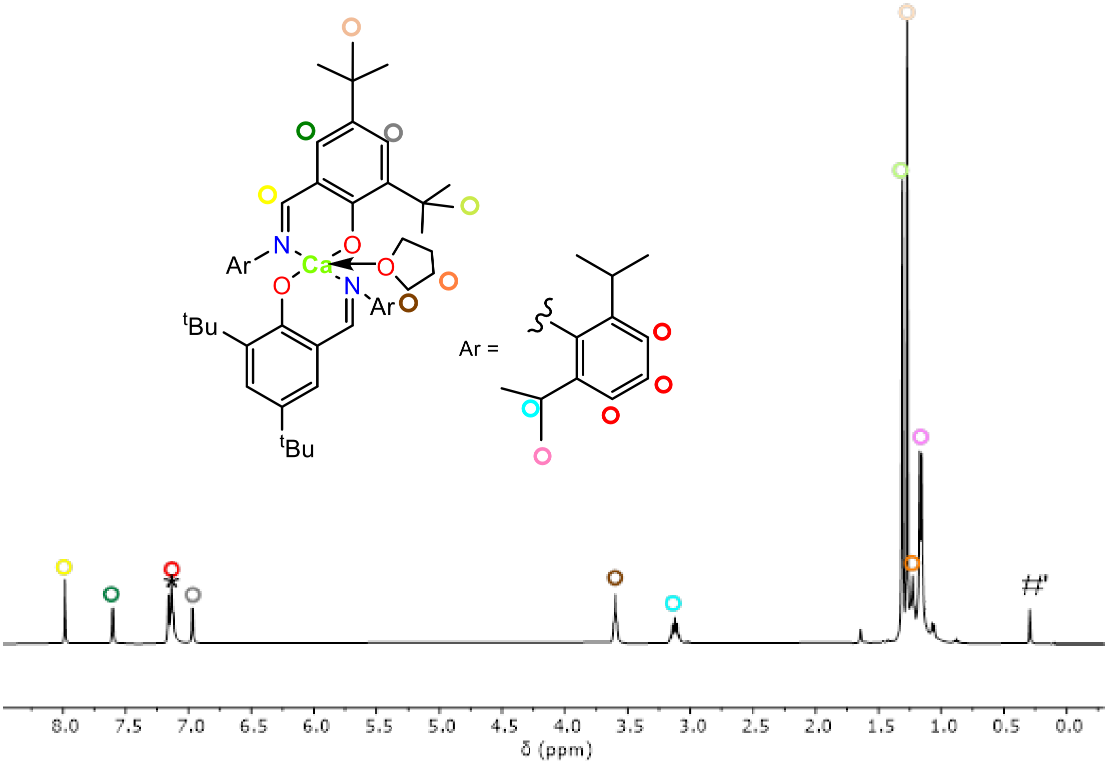

Figure S12. ^{1}H NMR spectrum (*C_6D_6, 400 MHz, 298 K) of (^{tBu_2,Dipp}L)$_2$Ca(thf) (**3**). #" represents residual HN".

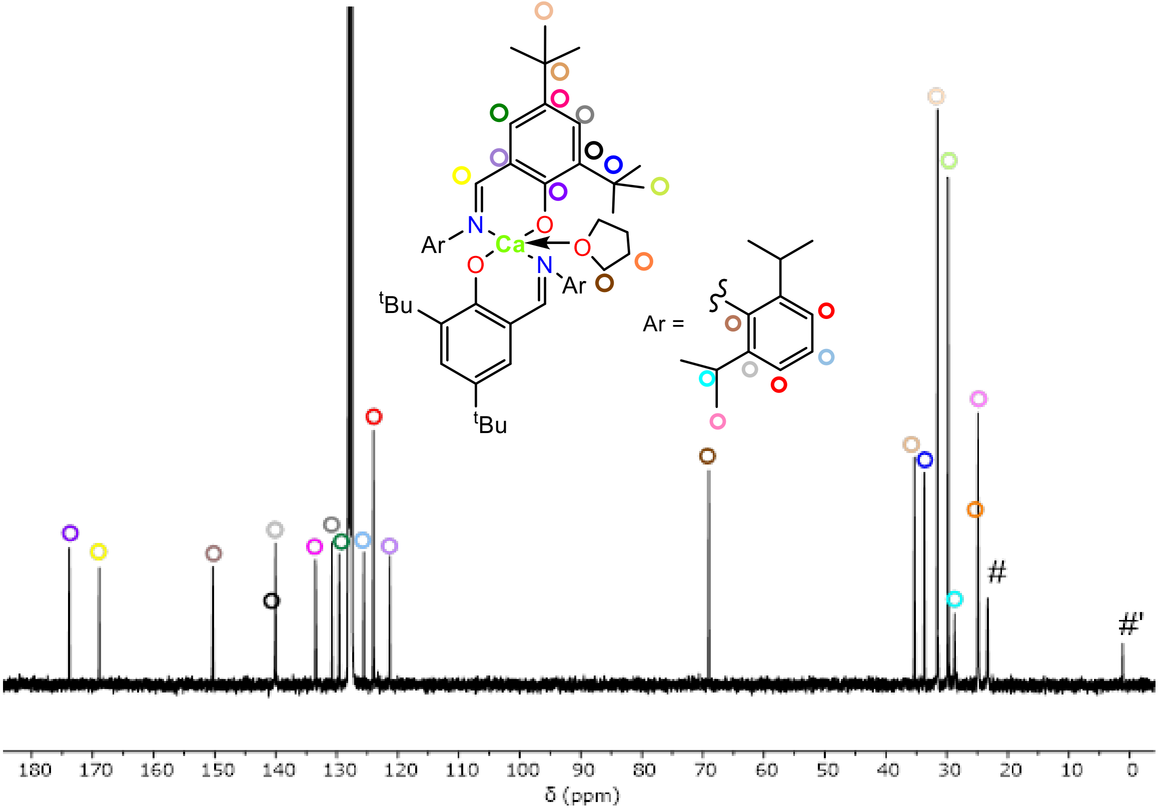

Figure S13. $^{13}C\{^1H\}$ NMR spectrum (C_6D_6, 101 MHz, 298 K) of ($^{tBu_2,Dipp}L$)$_2$Ca(thf) (**3**). # represents residual protio solvent (pentane), and #′ represents HN''.

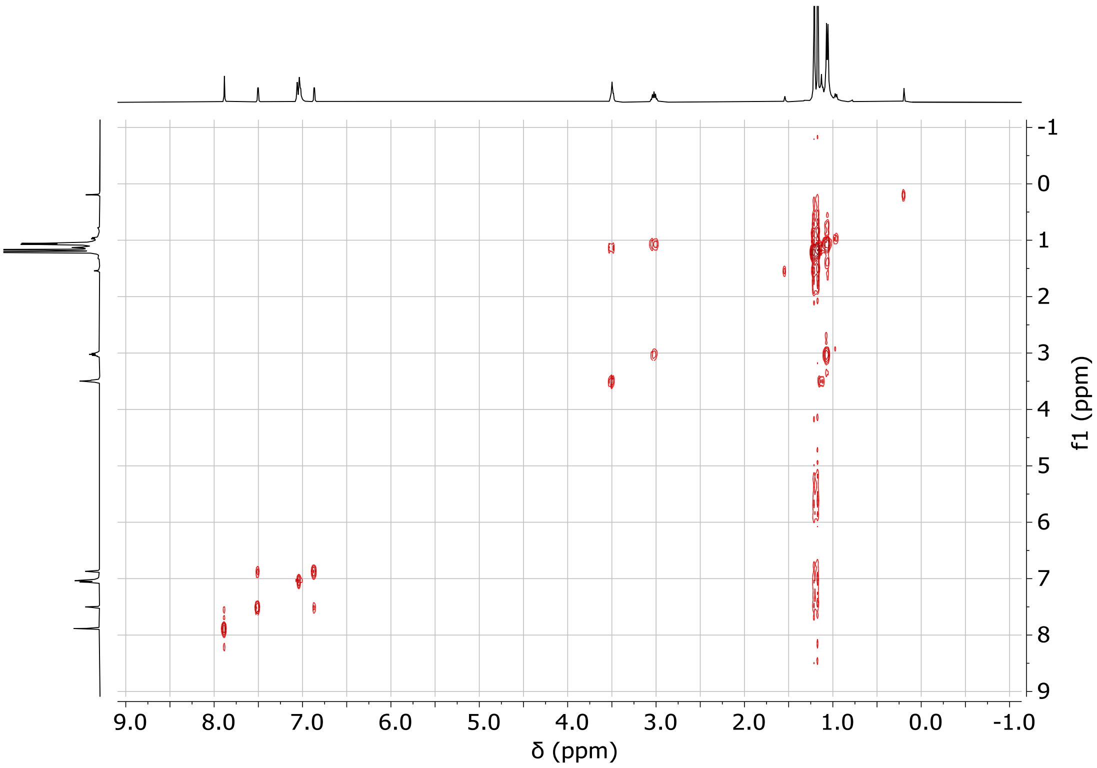

Figure S14. ^1H-^1H COSY NMR spectrum (C_6D_6, 400 MHz, 298 K) of ($^{tBu_2,Dipp}L$)$_2$Ca(thf) (**3**).

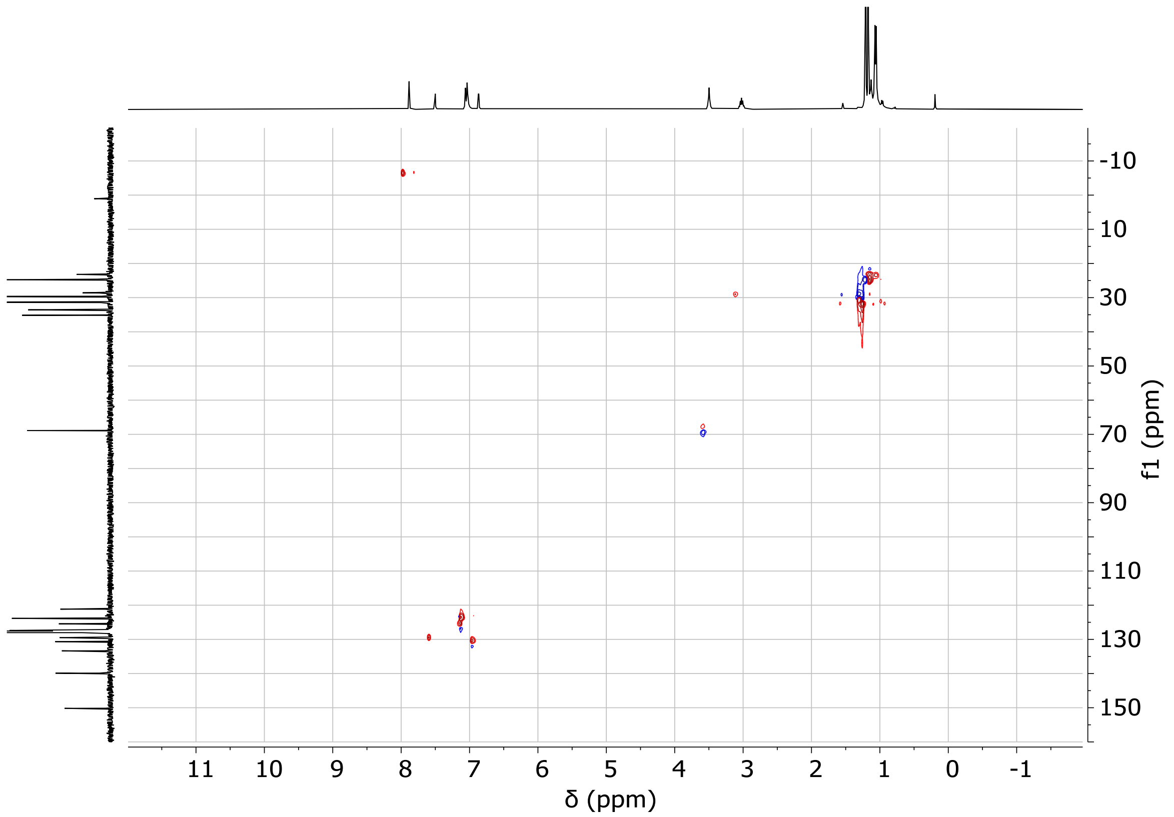

Figure S15. ^{1}H-^{13}C HSQC NMR spectrum (C_6D_6, 400 MHz, 298 K) of ($^{tBu2,Dipp}L)_2Ca(thf)$ (**3**).

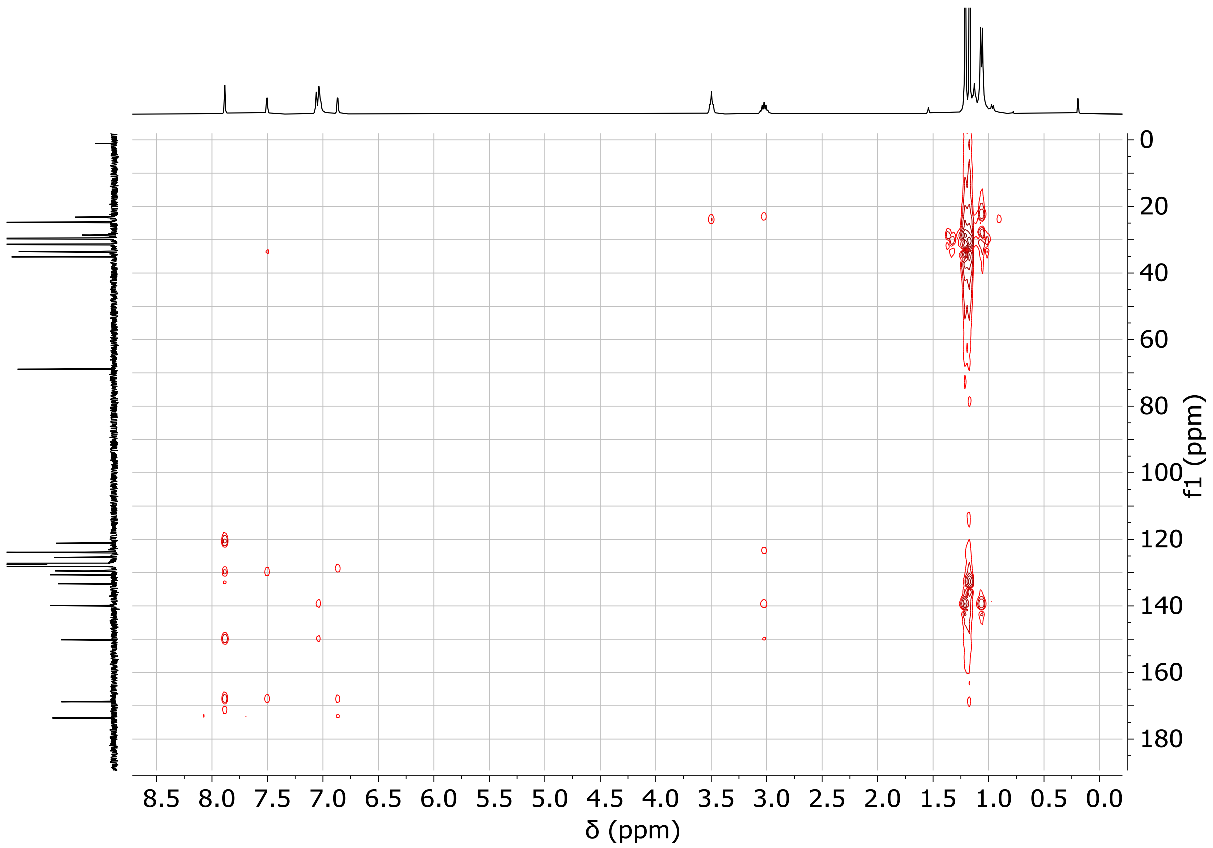

Figure S16. ^{1}H-^{13}H HMBC NMR spectrum (C_6D_6, 400 MHz, 298 K) of ($^{tBu2,Dipp}L)_2Ca(thf)$ (**3**).

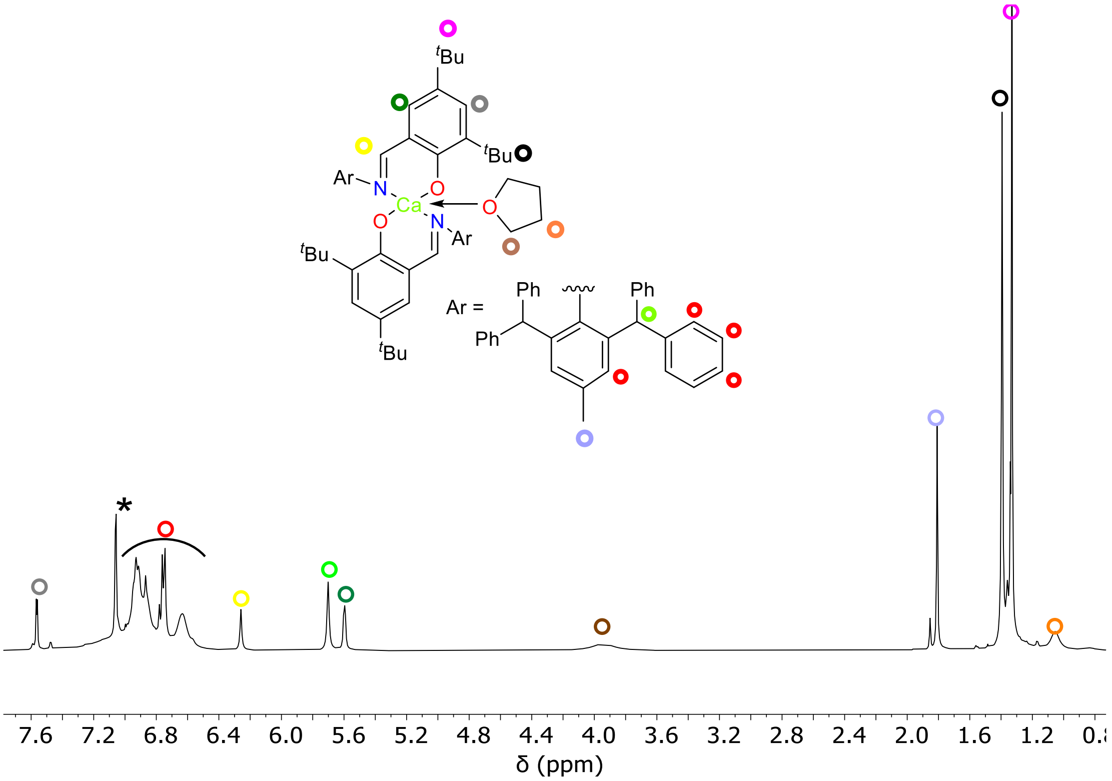

Figure S17. ^{1}H NMR spectrum ($*C_6D_6$, 400 MHz, 298 K) of $(^{tBu_2,Ar^*}L)_2Ca(thf)$ (**4**).

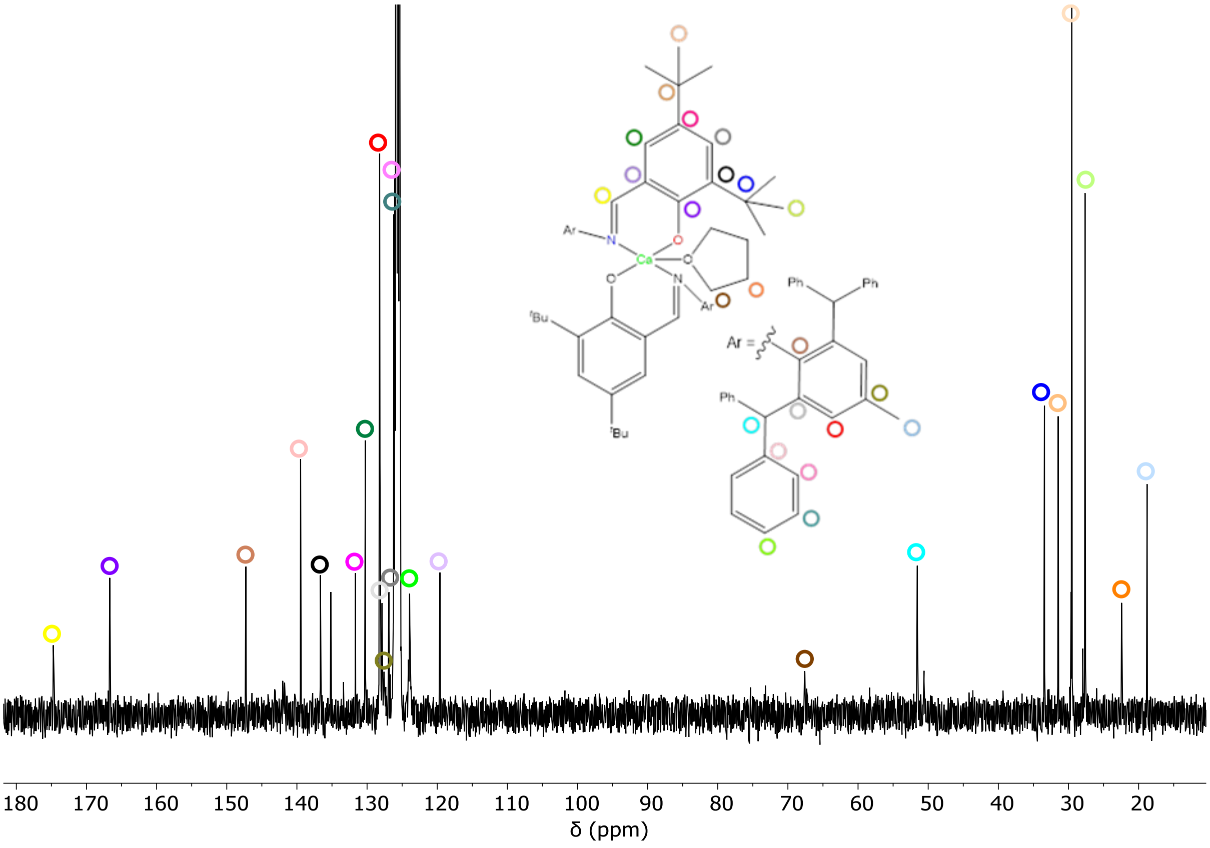

Figure S18. $^{13}C\{^{1}H\}$ NMR spectrum ($*C_6D_6$, 101 MHz, 298 K) of $(^{tBu_2,Ar^*}L)_2Ca(thf)$ (**4**).

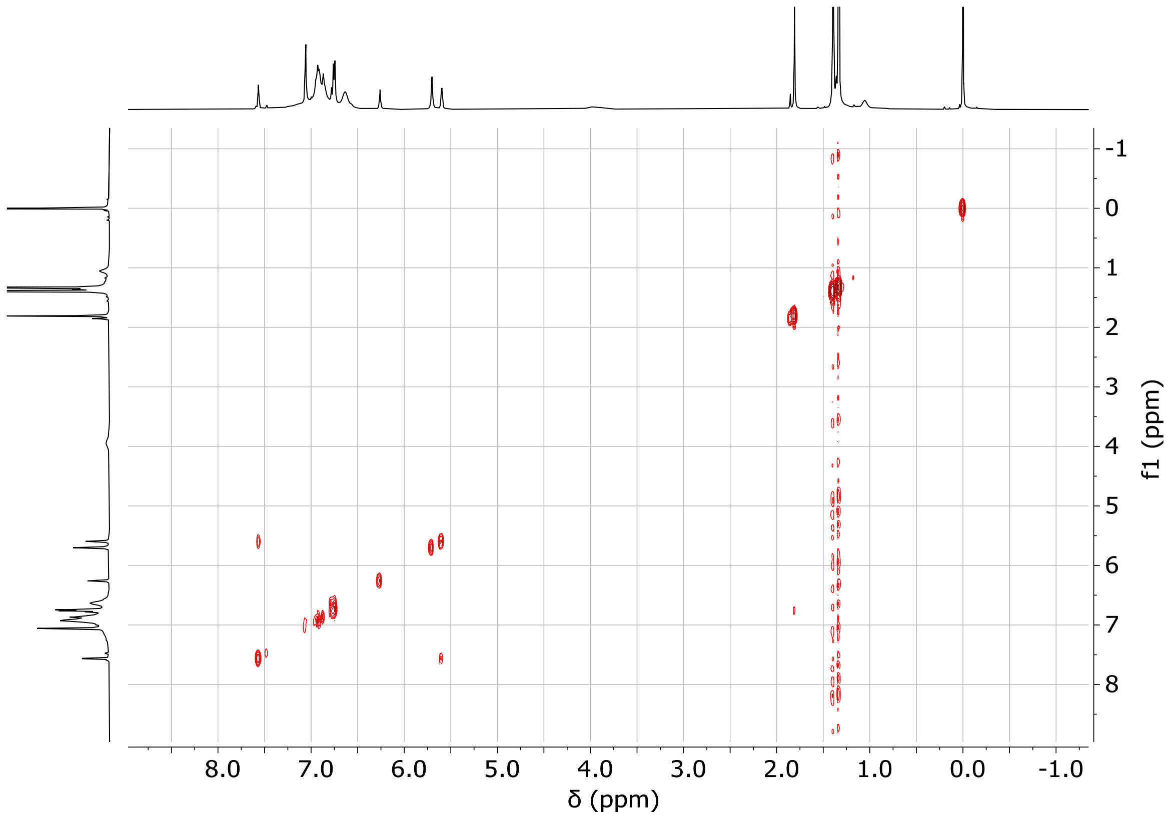

Figure S19. ^{1}H-^{1}H COSY NMR spectrum (C_6D_6, 400 MHz, 298 K) of ($^{tBu2,Ar^*}L$)$_2$Ca(thf) (**4**).

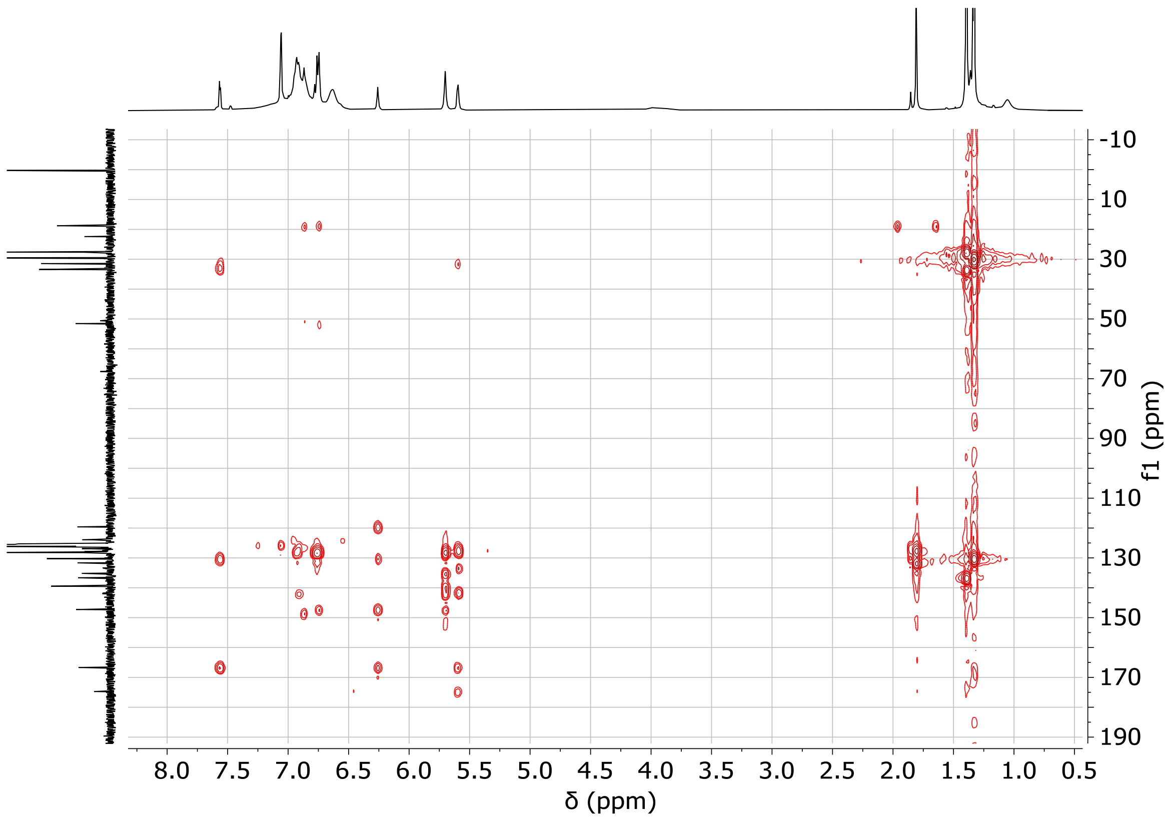

Figure S20. ^{1}H-^{13}C HSQC NMR spectrum (C_6D_6, 298 K) of ($^{tBu2,Ar^*}L$)$_2$Ca(thf) (**4**).

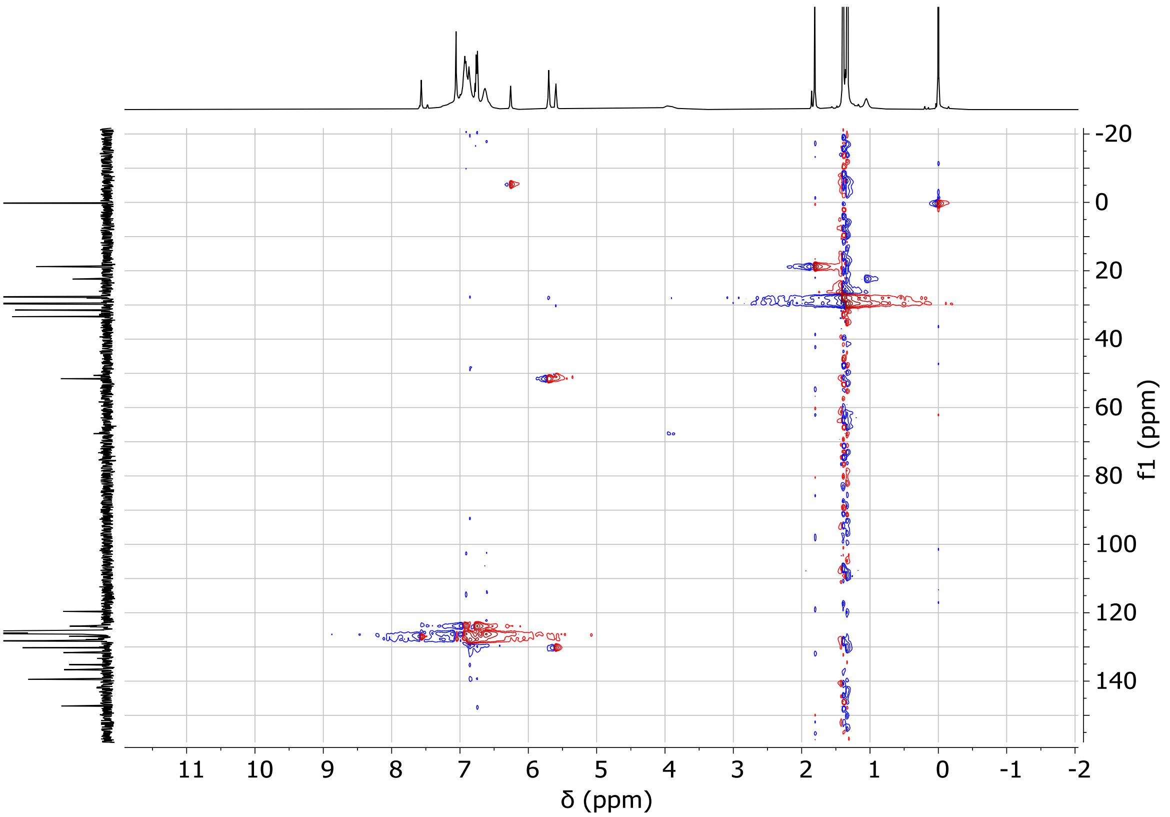

Figure S21. ^{1}H-^{13}C HMBC NMR spectrum (C_6D_6, 298 K) of ($^{tBu_2,Ar^*}$L)$_2$Ca(thf) (**4**).

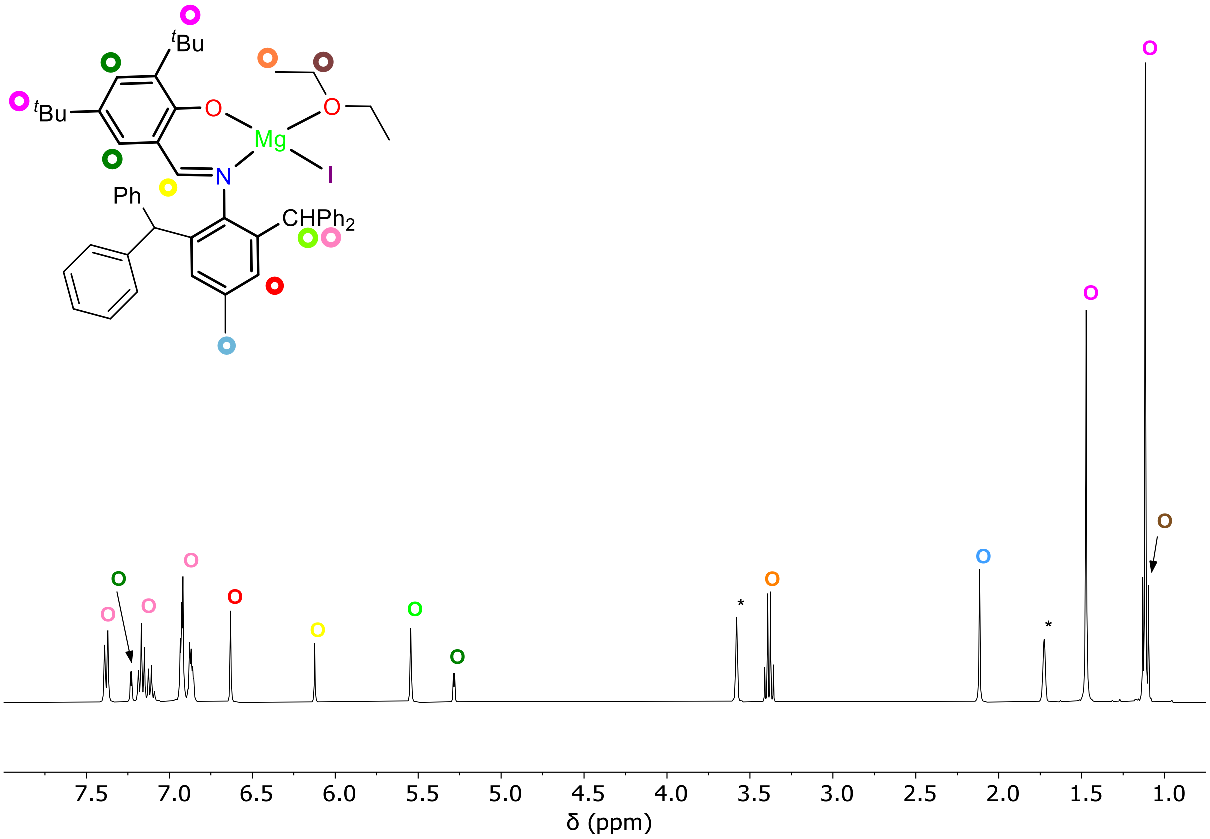

Figure S22. ^{1}H NMR spectrum (*C_4D_8O, 400 MHz, 298 K) of ($^{tBu_2,Ar^*}$L)MgI(OEt_2) (**5**).

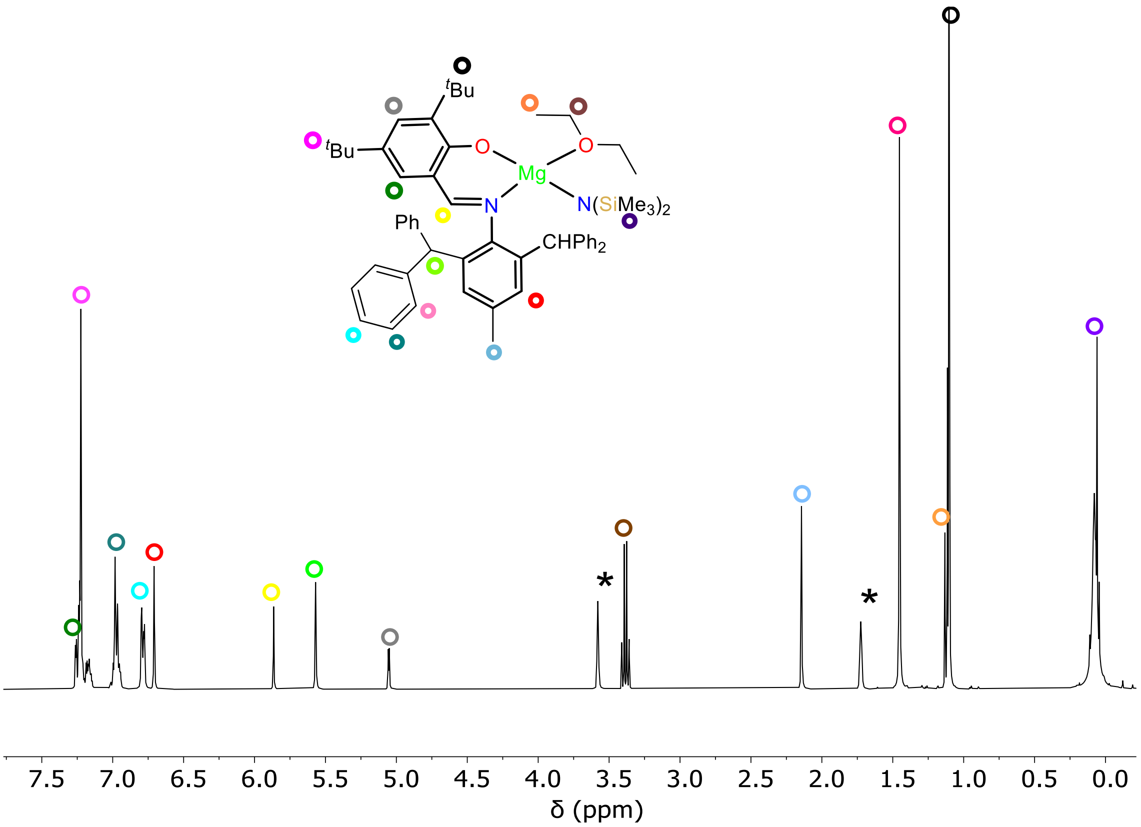

Figure S23. ^{1}H NMR spectrum (*C_4D_8O, 400 MHz, 298 K) of ($^{tBu_2,Ar^*}$L)MgN"(OEt$_2$) (**6**).

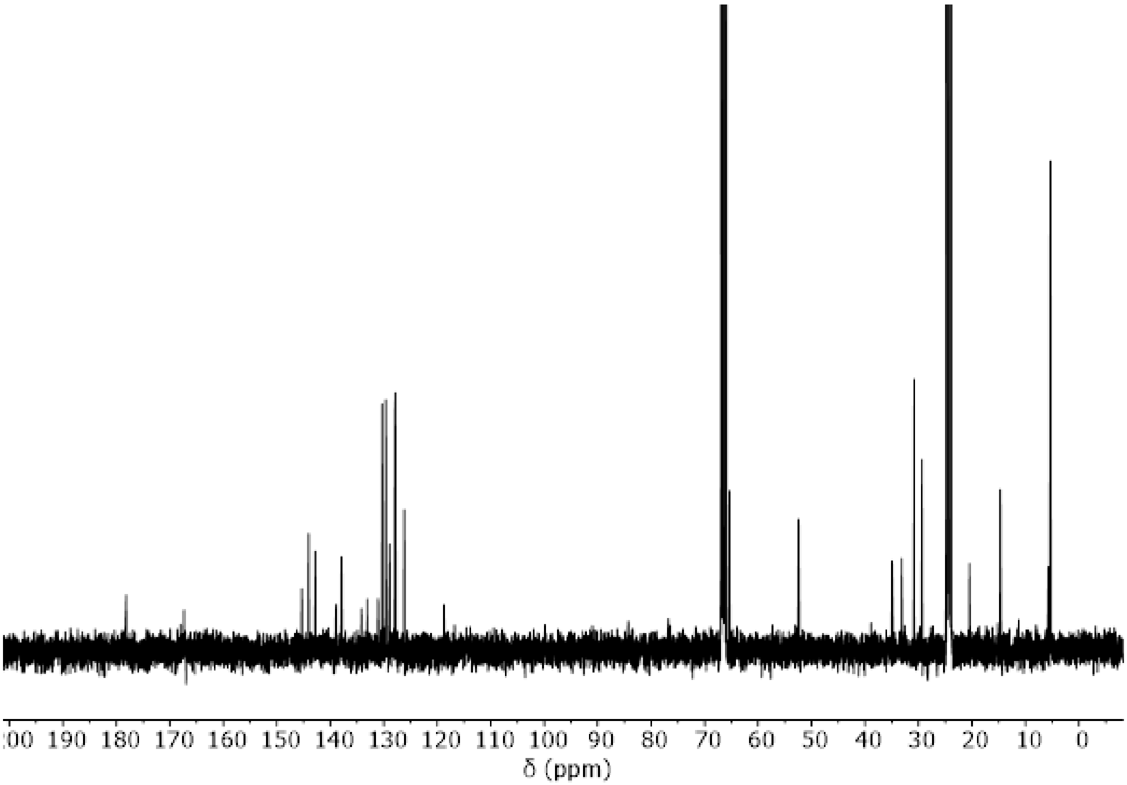

Figure S24. ^{13}C{^{1}H} NMR spectrum (C_4D_8O, 400 MHz, 298 K) of ($^{tBu_2,Ar^*}$L)MgN"(OEt$_2$) (**6**).

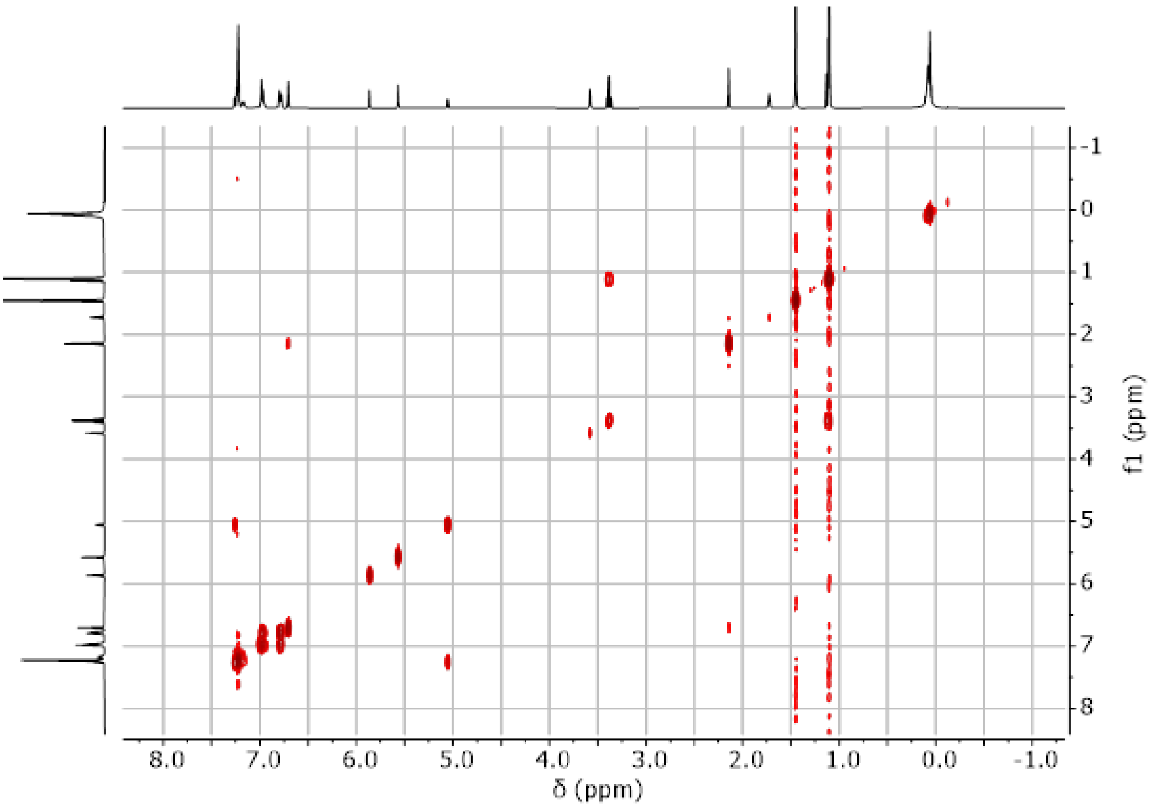

Figure S25. ^1H-^1H COSY NMR spectrum (C_4D_8O, 400 MHz, 298 K) of ($^{tBu_2,Ar^*}L$)MgN''(OEt_2) (**6**).

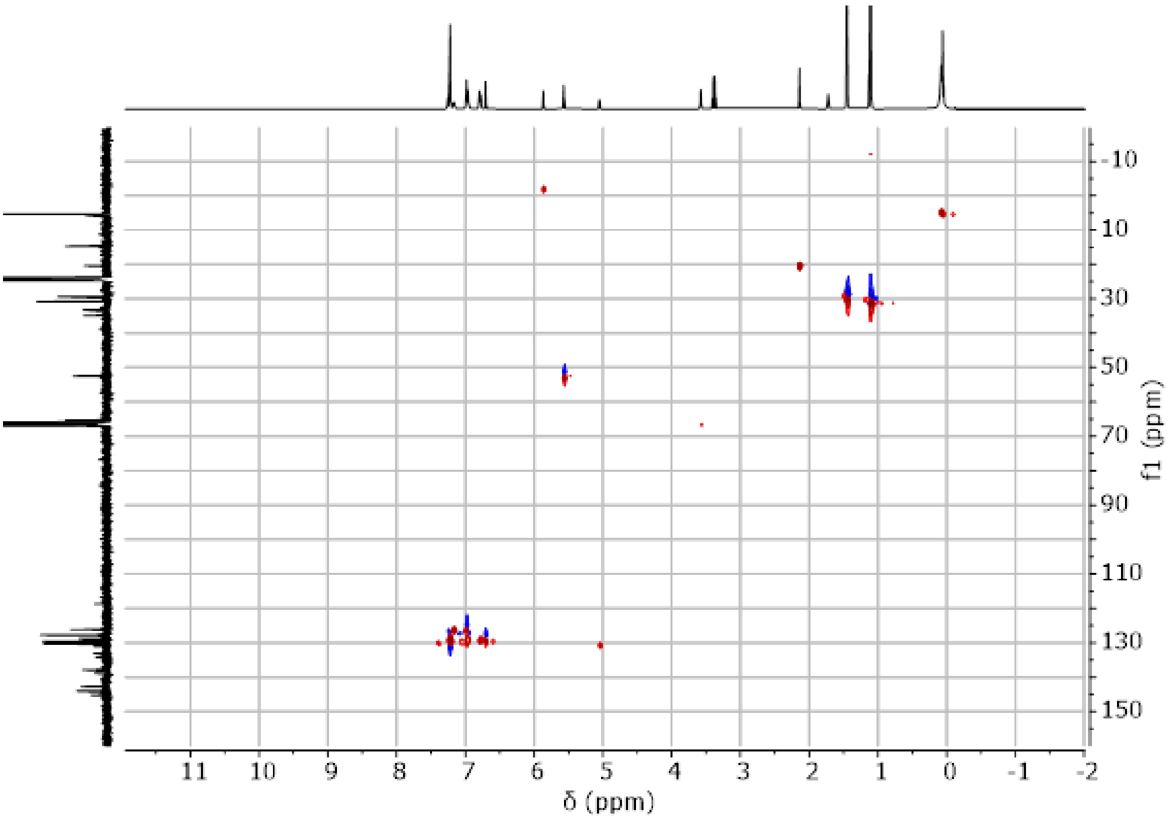

Figure S26. ^1H-^{13}C HSQC NMR spectrum (C_4D_8O, 298 K) of ($^{tBu_2,Ar^*}L$)MgN''(OEt_2) (**6**).

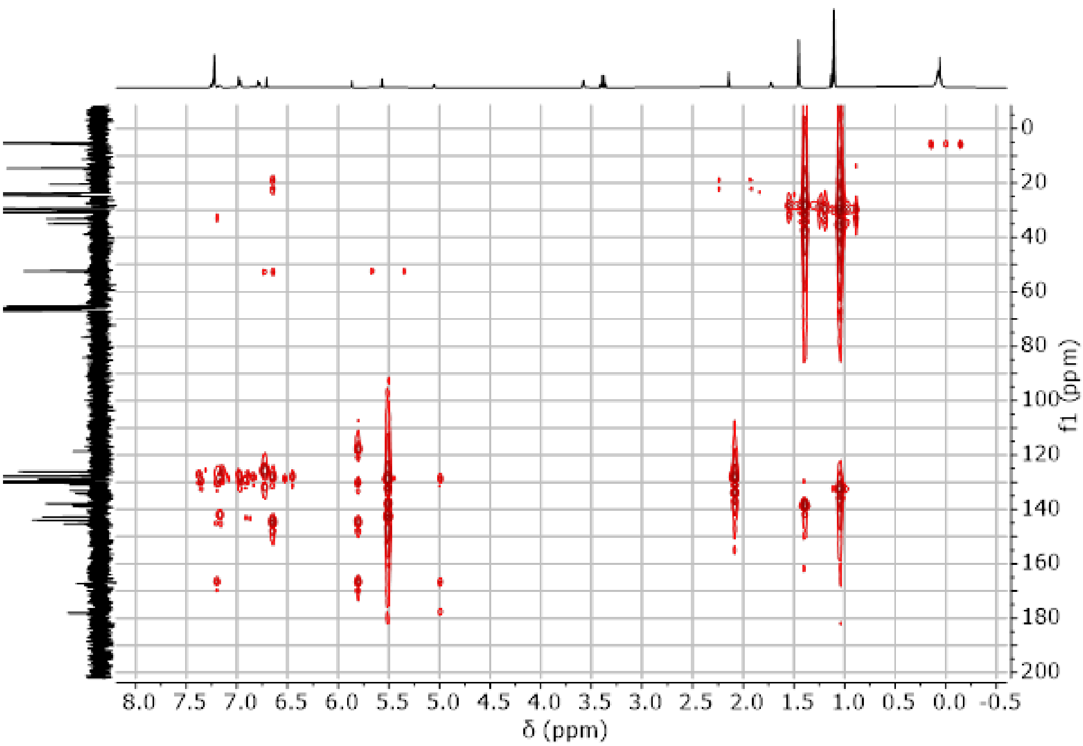

Figure S27. ^{1}H-^{13}C HMBC NMR spectrum (C_4D_8O, 298 K) of $(^{tBu2,Ar^{*}}L)MgN''(OEt_2)$ (**6**).

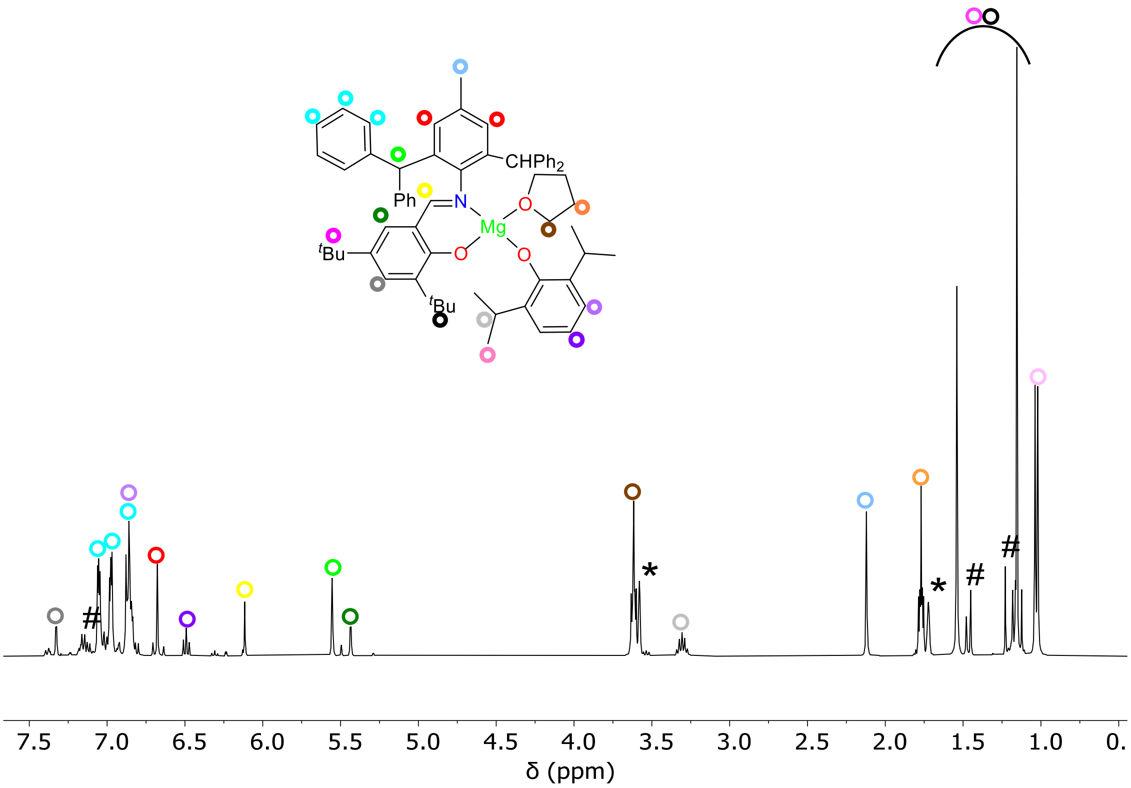

Figure S28. ^{1}H NMR spectrum (*C_4D_8O, 400 MHz, 298 K) of $(^{tBu2,Ar^{*}}L)Mg(ODipp)(thf)$ (**7**). # indicates residual $(^{tBu2,Ar^{*}}L)MgI(OEt_2)$ starting material.

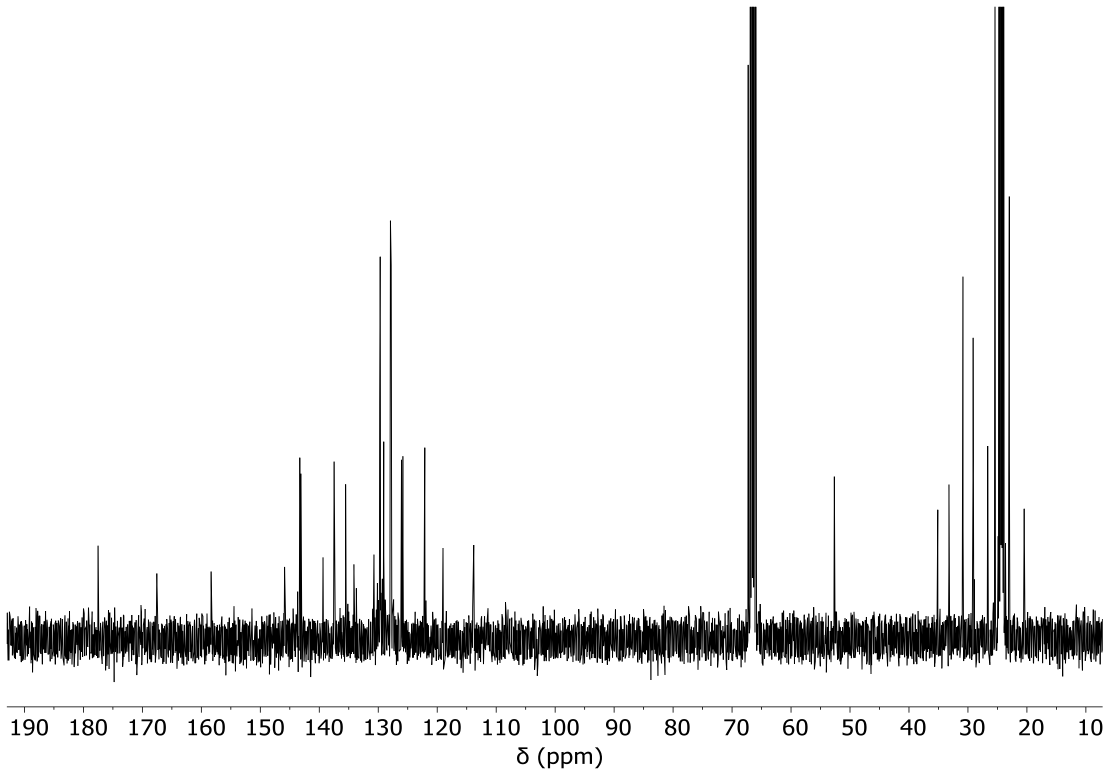

Figure S29. $^{13}C\{^{1}H\}$ NMR spectrum (C_4D_8O, 400 MHz, 298 K) of ($^{tBu2,Ar^*}$L)Mg(ODipp)(thf) (**7**).

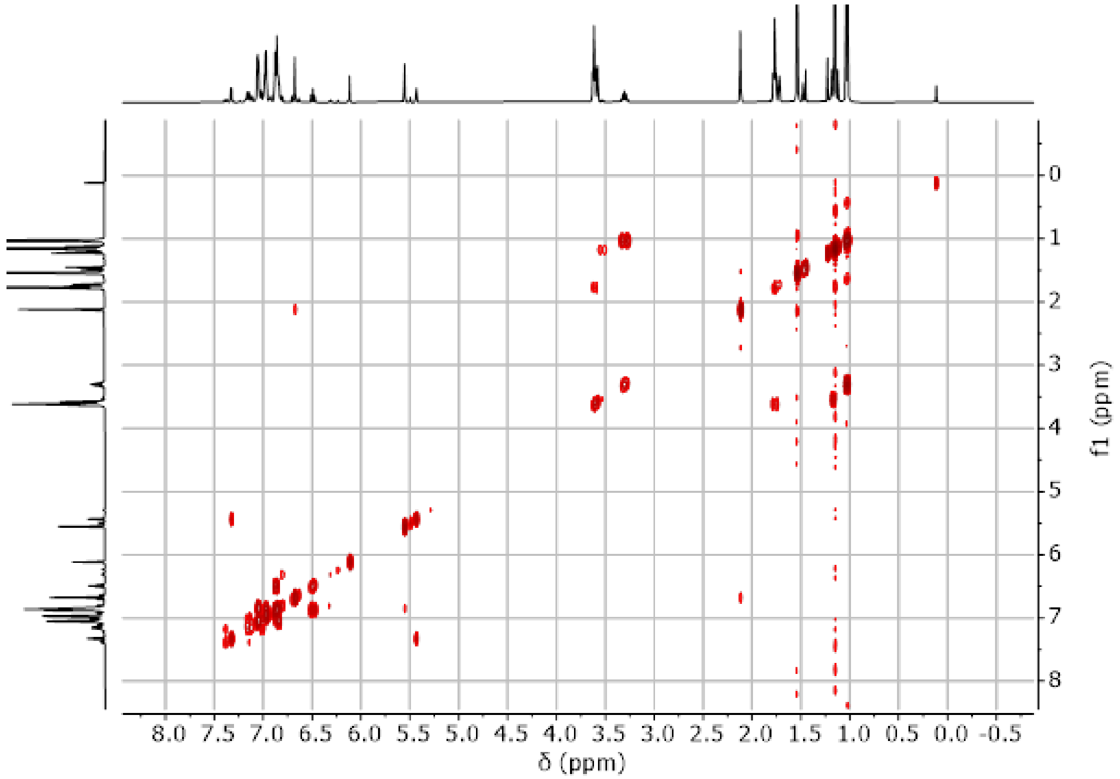

Figure S30. ^{1}H-^{1}H COSY NMR spectrum (C_4D_8O, 400 MHz, 298 K) of ($^{tBu2,Ar^*}$L)Mg(ODipp)(thf) (**7**).

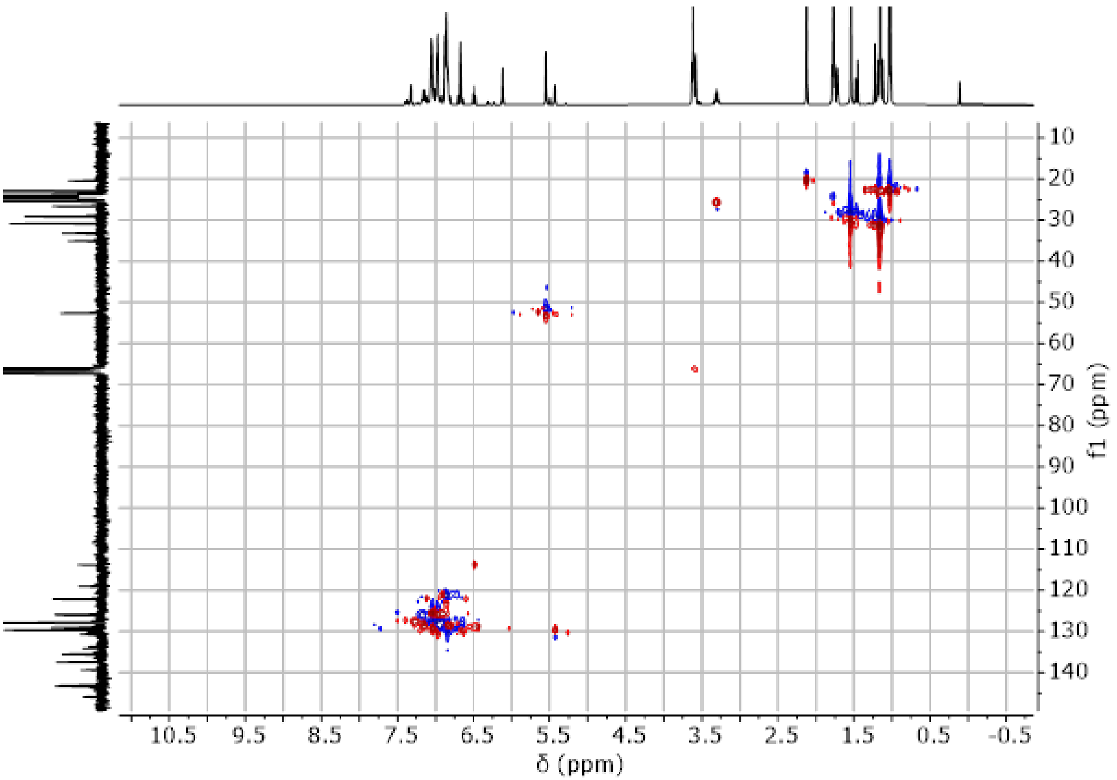

Figure S31. ^{1}H-^{13}C HSQC NMR spectrum (C_4D_8O, 298 K) of ($^{tBu_2,Ar^*}L$)Mg(ODipp)(thf) (**7**).

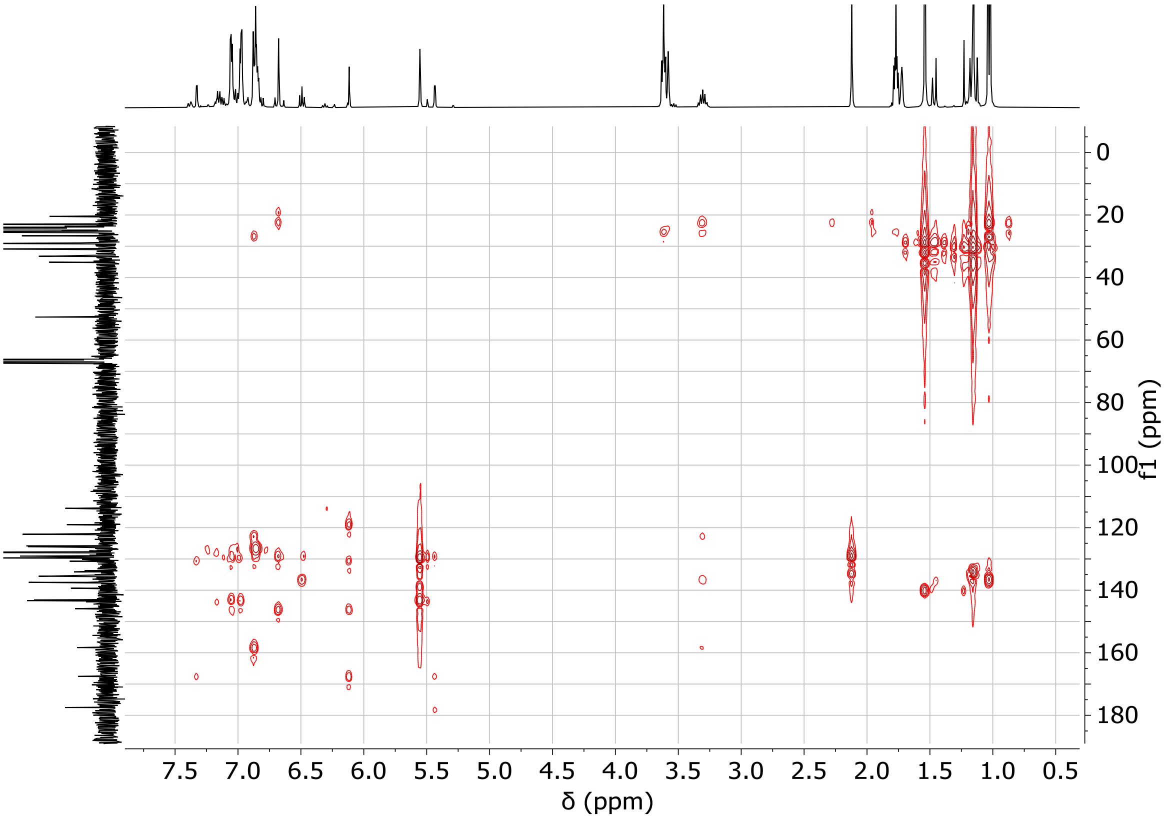

Figure S32. ^{1}H-^{13}C HMBC NMR spectrum (C_4D_8O, 298 K) of ($^{tBu_2,Ar^*}L$)Mg(ODipp)(thf) (**7**).

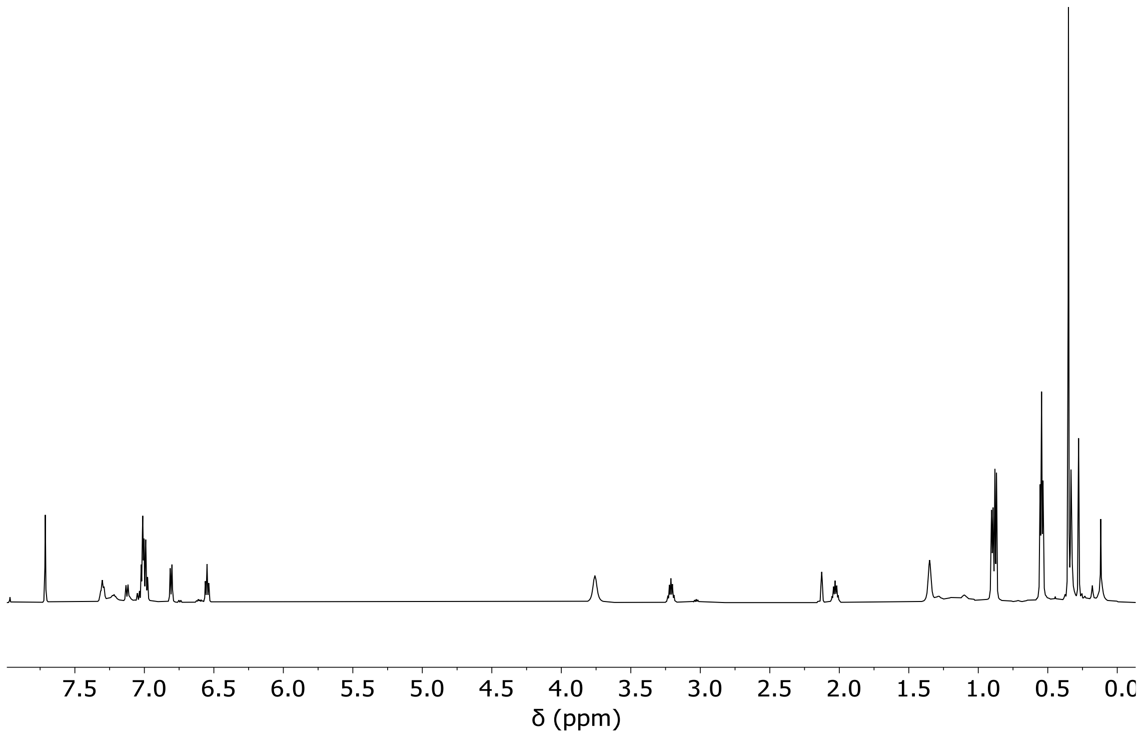

Figure S33. ^{1}H NMR spectrum (C_7D_8, 600 MHz, 298 K) of ($^{H2,Dipp}L)_3Ca_2(N'')$(thf) (**8**).

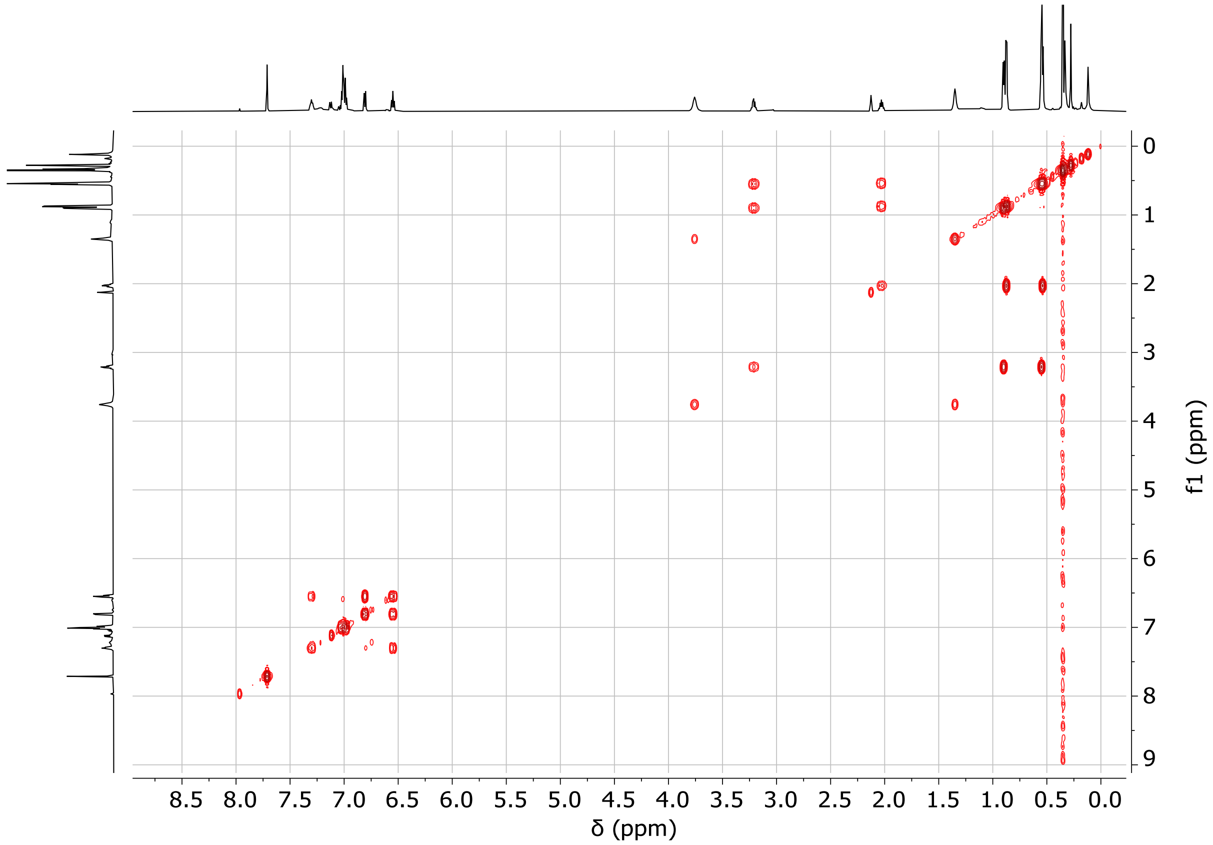

Figure S34. ^{1}H-^{1}H COSY NMR spectrum (C_7D_8, 600 MHz, 298 K) of ($^{H2,Dipp}L)_3Ca_2(N'')$(thf) (**8**).

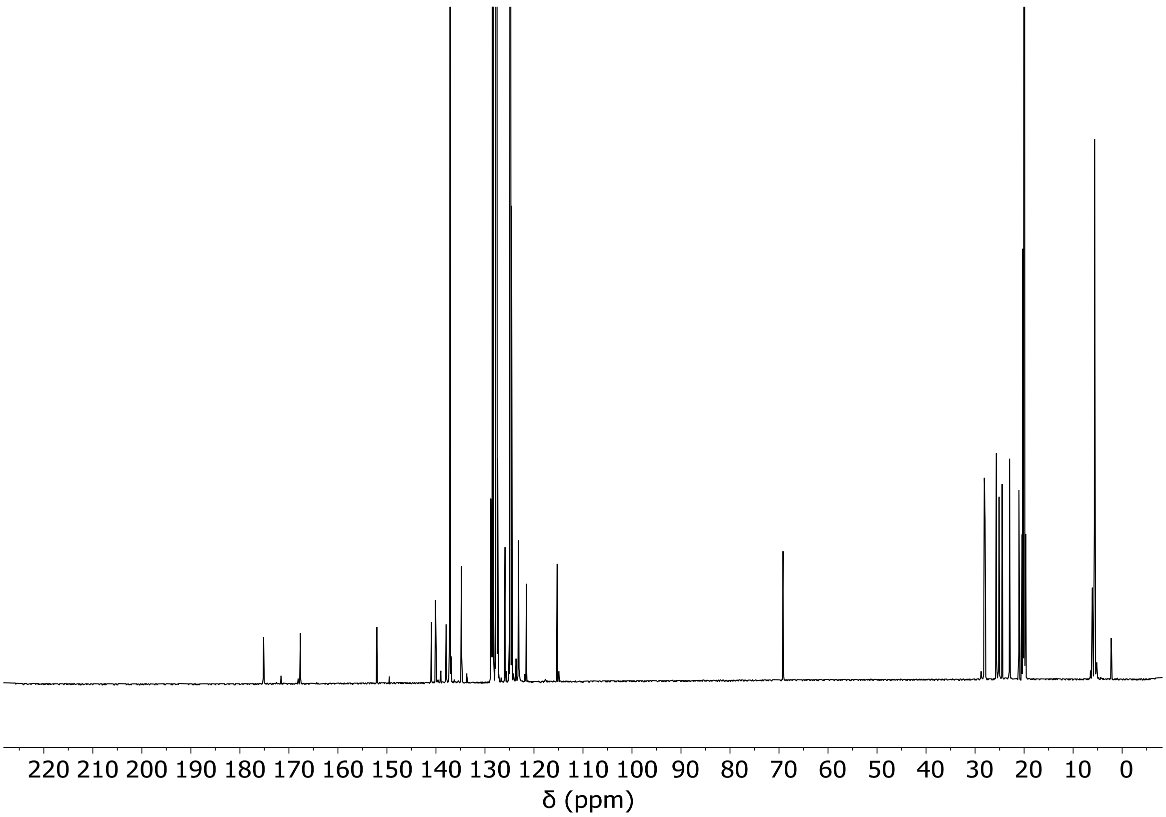

Figure S35. $^{13}C\{^{1}H\}$ NMR spectrum (C_7D_8, 151 MHz, 298 K) of ($^{H_2,Dipp}L)_3Ca_2(N'')(thf)$ (**8**).

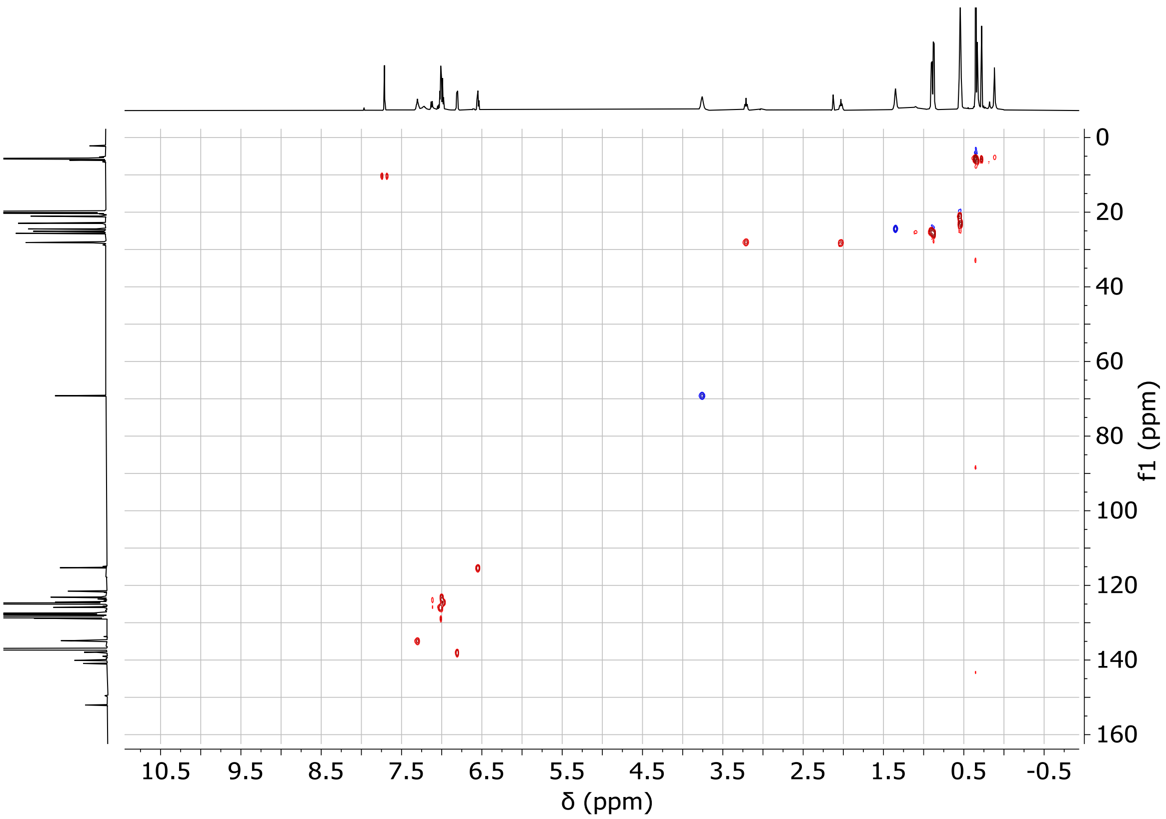

Figure S36. ^{1}H-^{13}C HSQC NMR spectrum (C_7D_8, 600 MHz, 298 K) of ($^{H_2,Dipp}L)_3Ca_2(N'')(thf)$ (**8**).

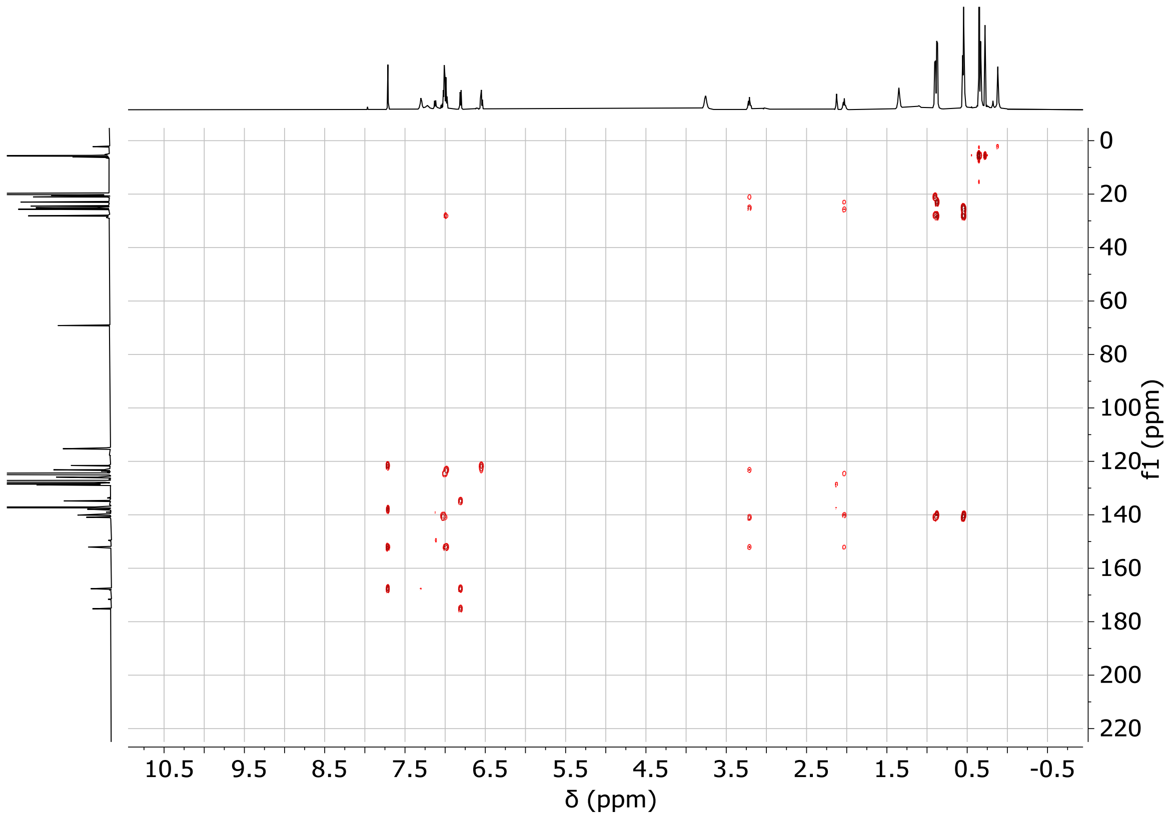

Figure S37. ^{1}H-^{13}C HMBC NMR spectrum (C_7D_8, 600 MHz, 298 K) of $(^{H_2,Dipp}L)_3Ca_2(N'')(thf)$ (**8**).

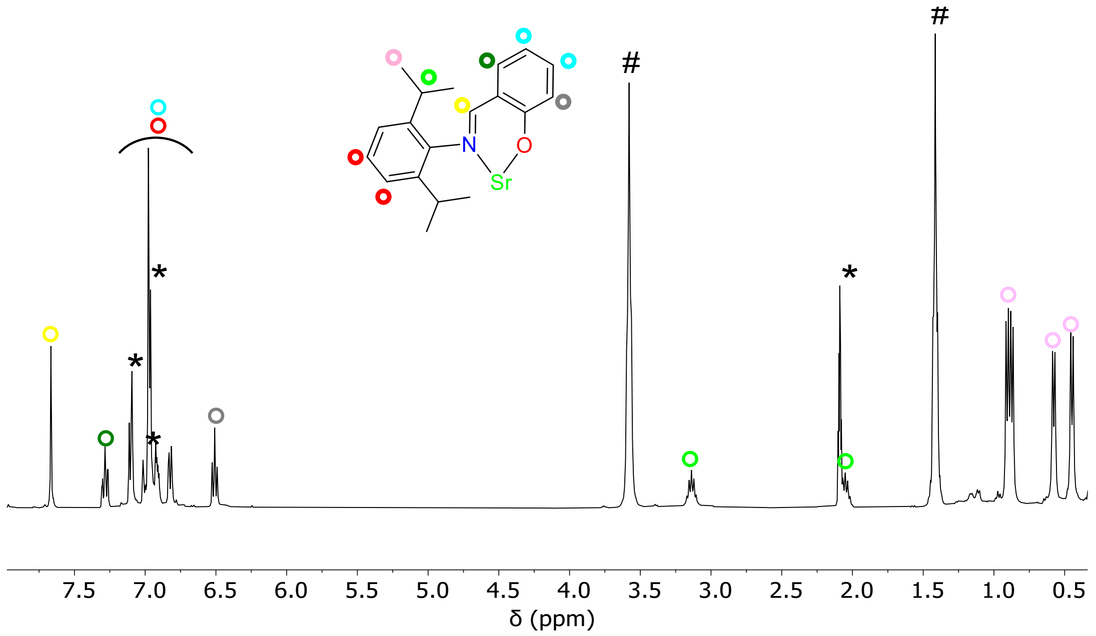

Figure S38. ^{1}H NMR spectrum (C_7D_8, 600 MHz, 298 K) of $(^{Dipp}L)_6Sr_3$ (**9**).

III. Additional synthetic and characterising details

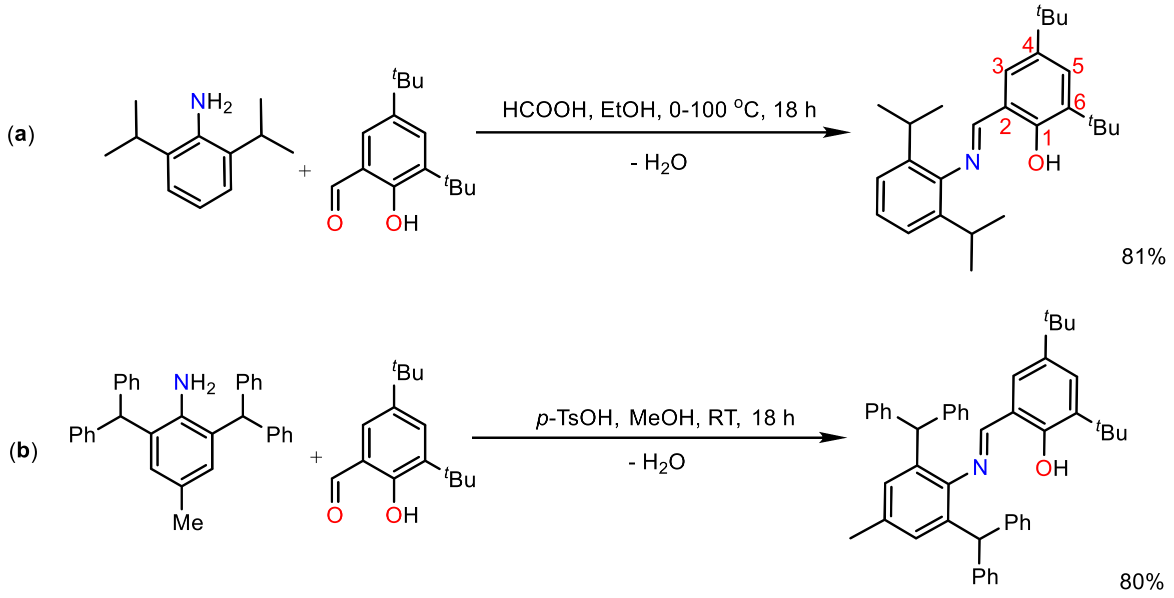

Scheme S1. Overview of the synthesis of proligand $H^{tBu_2,Dipp}L$ and $H^{tBu_2,Ar^*}L$.

$H^{tBu_2,Ar^*}L$ **(2-{(*E*)-2,6-diphenylmethyl-4-methyl-phenylimino-methylene}-4,6-di-*tert*-butyl-phenol; 1-OH-2-{C(H)=N-2,6-CH(C_6H_5)$_2$-4-Me-C_6H_3}-4,6-^{t}Bu-C_6H_2)**]

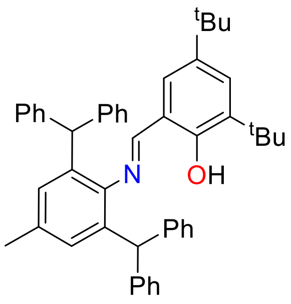

modified from reference 7. 3,5-di-*tert*-butylsalicylaldehyde (2.65 g, 11.4 mmol) was added to a solution of 2,6-bis(diphenylmethyl)-4-methylaniline (5.00 g, 11.4 mmol) in methanol (150 mL). *para*-Toluenesulfonic acid (100 mg) was added and the reaction mixture was heated under reflux, at 75 °C, for 18 h. The resultant precipitate was filtered and dried *in vacuo*, affording pro-ligand $H^{tBu_2,Ar^*}L$ as a pale-yellow powder (5.97 g, 80%). Single crystals of $H^{tBu_2,Ar^*}L$, suitable for an X-ray diffraction study, were obtained by the slow evaporation of a saturated ethanol solution at room temperature. **^{1}H NMR** (chloroform-*d*, 600 MHz, 298 K): δ 12.93 (s, 1H, O***H***), 7.38 (d, $^4J_{H\text{-}H}$ = 2.5 Hz, 1H, 5-C_6***H***$_2$), 7.22 (m, 8H, CH(3,5-C_6***H***$_5$)$_2$), 7.18 (m, 4H, CH(4-C_6***H***$_5$)$_2$), 7.06 (m, 8H, CH(2,6-C_6***H***$_5$)$_2$), 6.93 (s, 1H, ***H***C=N), 6.71 (s, 2H, C_6***H***$_2$), 6.17 (d, $^4J_{H\text{-}H}$ = 2.5 Hz, 1H, 3-C_6***H***$_2$), 5.51 (s, 2H, C***H***(C_6H_5)$_2$), 2.20 (s, 3H, 4-***Me***-C_6H_2), 1.50 (s, 9H, 4- C***Me***$_3$-C_6H_2), 1.26 (s, 9H, 6-C***Me***$_3$-C_6H_2) ppm. **^{13}C{^{1}H} NMR** (chloroform-*d*$_6$, 151 MHz, 298 K): δ 169.95 (H***C***=N), 157.79 (1-

C$_6$H$_2$), 146.06 (N-1-***C***$_6$H$_2$), 143.55 (CH(1-***C***$_6$H$_5$)$_2$), 139.63 (6-***C***$_6$H$_2$), 136.23 (4-***C***$_6$H$_2$), 134.90 (N-2,6-***C***$_6$H$_2$), 133.24 (N-4-***C***$_6$H$_2$), 129.68 (CH(2,6-***C***$_6$H$_5$)$_2$), 128.87 (N-3,5-***C***$_6$H$_2$), 128.13 (CH(3,5-***C***$_6$H$_5$)$_2$), 127.34 (5-***C***$_6$H$_2$), 127.04 (3-***C***$_6$H$_2$), 126.06 (CH(4-***C***$_6$H$_5$)$_2$), 117.40 (2-***C***$_6$H$_2$), 52.24 (***C***H(C$_6$H$_5$)$_2$), 34.97 (4-***C***Me$_3$-C$_6$H$_2$), 33.94 (6-***C***Me$_3$-C$_6$H$_2$), 31.37 (6-C***Me***$_3$-C$_6$H$_2$), 29.41 (4-C***Me***$_3$-C$_6$H$_2$), 21.37 (4-***Me***-C$_6$H$_2$) ppm. Anal. Calcd for $C_{48}H_{49}NO$: C, 87.90; H, 7.53; N, 2.14. Found: C, 88.40; H, 7.44; N, 2.07.

IV. Crystallographic data

Crystals were mounted on MiTeGen MicroMounts using perfluoropolyether oil and rapidly transferred to a goniometer head on a diffractometer fitted with an Oxford Cryostream open-flow nitrogen cooling device.[8] Data collections were carried out at 150 K using an Oxford Diffraction Supernova using mirror-monochromated Cu Kα radiation (λ = 1.54178 Å) and the data processed using CrysAlisPro.[9] The structures were solved using direct methods (SIR-92),[10] a flipping algorithm (SUPERFLIP),[11] or intrinsic phasing (SHELXT)[12] and refined on F^2 by full-matrix least squares procedures using the WinGX software suite,[13] CRYSTALS,[14] or the SHELXL[15] refinement package implemented in Olex2.[18] Molecular structures were generated using ORTEP (with ellipsoids shown at 30% probability).[16] Geometric calculations were performed using PLATON.[17] Solid state structural data have been deposited in the Cambridge Crystallographic Data Centre (2409489–2409499).

Table S1. Selected experimental crystallographic data.

Complex	$H^{H_2,Ar^*}L$ **(reported in reference 18)**	$H^{^tBu_2,Ar^*}L$	$(^{tBu_2,Dipp}L)_2Mg$ **(1)**
**Crystal data**			
Chemical formula	$C_{40}H_{33}NO$	$C_{48}H_{49}NO$	$C_{54}H_{76}MgN_2O$, $2(C_6H_6)$
M_r	543.67	655.88	965.69
Crystal system, space group	Monoclinic, $P2_1/c$	Monoclinic, $C2/c$	Triclinic, P¯1
Temperature (K)	150	150	150
a, b, c (Å)	10.8164 (2), 17.7819 (2), 16.1528 (2)	27.5265 (6), 15.0897 (3), 18.3223 (4)	14.1132 (6), 15.4327 (8), 15.5328 (7)
α, β, γ (°)	90, 108.096 (1), 90	90, 91.439 (2), 90	88.252 (4), 84.231 (4), 77.142 (4)
V (Å^3)	2953.10 (8)	7608.1 (3)	3281.5 (3)
Z	4	8	2
Radiation type	Cu *K*a	Cu *K*a	Cu *K*a
μ (mm^{-1})	0.56	0.51	1.11
Crystal size (mm)	0.32 × 0.19 × 0.04	0.26 × 0.16 × 0.12	0.28 × 0.18 × 0.14
**Data Collection**			
Diffractometer	SuperNova, Dual, Cu at zero, Atlas diffractometer	SuperNova, Dual, Cu at zero, Atlas diffractometer	SuperNova, Dual, Cu at zero, Atlas
Absorption correction	Gaussian *CrysAlis PRO* 1.171.41.93a (Rigaku Oxford Diffraction, 2020) Numerical absorption correction based on gaussian integration over a multifaceted crystal model Empirical absorption correction using spherical harmonics, implemented in SCALE3 ABSPACK scaling algorithm.	Gaussian *CrysAlis PRO* 1.171.41.93a (Rigaku Oxford Diffraction, 2020) Numerical absorption correction based on gaussian integration over a multifaceted crystal model Empirical absorption correction using spherical harmonics, implemented in SCALE3 ABSPACK scaling algorithm.	Gaussian *CrysAlis PRO* 1.171.41.93a (Rigaku Oxford Diffraction, 2020) Numerical absorption correction based on gaussian integration over a multifaceted crystal model Empirical absorption correction using spherical harmonics, implemented in SCALE3 ABSPACK scaling algorithm.
T_{min}, T_{max}	0.586, 1.000	0.682, 1.000	0.520, 1.000
No. of measured, independent and observed [$I > 2\sigma(I)$] reflections	29275, 6038, 5172	26431, 7758, 6144	26105, 12434, 8832
R_{int}	0.029	0.034	0.038
**Refinement**			
$R[F^2 > 2\sigma(F^2)]$, $wR(F^2)$, S	0.044, 0.123, 1.06	0.053, 0.149, 1.01	0.057, 0.176, 1.03
No. of reflections	6038	7758	12434
No. of parameters	463	498	666
No. of restraints	0	42	0
$(\Delta/\sigma)_{max}$	< 0.001	< 0.001	0.001
$\Delta\rho_{max}$, $\Delta\rho_{min}$ (e Å^{-3})	0.41, -0.34	0.67, -0.25	0.35, -0.27

Complex	$(^{tBu_2,Ar*}L)_2Mg$ **(2)**	$(^{tBu_2,Dipp}L)_2Ca(thf)$ **(3)**	$(^{tBu_2,Ar*}L)_2Ca(thf)$ **(4)**
**Crystal data**			
Chemical formula	$C_{96}H_{96}MgN_2O_2{\cdot}5(C_6H_6)$	$C_{58}H_{84}CaN_2O_3$, $3(C_6H_6)$	$2(C_{100}H_{104}CaN_2O_3){\cdot}4.5(C_6H_6)$
M_r	1724.59	1131.67	3195.36
Crystal system, space group	Monoclinic, $P2_1/c$	Triclinic, $P\bar{1}$	Monoclinic, $P2_1/n$
Temperature (K)	150	150	150
a, b, c (Å)	18.7585 (3), 20.2567 (3), 28.0614 (5)	13.1733 (5), 13.8009 (4), 20.4209 (8)	15.9271 (1), 20.8110 (1), 29.4066 (1)
α, β, γ (°)	90, 109.460 (2), 90	79.473 (3), 74.304 (3), 88.383 (3)	90, 102.5203 (5), 90
V (Å^3)	10053.8 (3	3513.1 (2)	9515.29 (8)
Z	4	2	2
Radiation type	Cu *K*a	Cu *K*a	Cu *K*a
μ (mm^{-1})	0.56	1.11	0.96
Crystal size (mm)	0.38 × 0.28 × 0.19	0.46 × 0.23 × 0.14	0.38 × 0.29 × 0.15
**Data Collection**			
Diffractometer	SuperNova, Dual, Cu at zero, Atlas	SuperNova, Dual, Cu at zero, Atlas	SuperNova, Dual, Cu at zero, Atlas
Absorption correction	Gaussian	Gaussian	Multi-scan
	CrysAlis PRO 1.171.41.93a (Rigaku Oxford Diffraction, 2020) Numerical absorption correction based on gaussian integration over a multifaceted crystal model Empirical absorption correction using spherical harmonics, implemented in SCALE3 ABSPACK scaling algorithm.	*CrysAlis PRO* 1.171.41.93a (Rigaku Oxford Diffraction, 2020) Numerical absorption correction based on gaussian integration over a multifaceted crystal model Empirical absorption correction using spherical harmonics, implemented in SCALE3 ABSPACK scaling algorithm.	*CrysAlis PRO* (Rigaku Oxford Diffraction, 2021)
T_{min}, T_{max}	0.442, 1.000	0.412, 1.000	0.66, 0.87
No. of measured, independent and observed [$I > 2\sigma(I)$] reflections	116593, 20551, 15093	116593, 20551, 15093	234030, 19813, 17365
R_{int}	0.047	0.047	0.041
**Refinement**			
$R[F^2 > 2\sigma(F^2)]$, $wR(F^2)$, S	0.046, 0.134, 1.03	0.057, 0.169, 1.05	0.047, 0.141, 0.98
No. of reflections	20551	14350	19797
No. of parameters	1194	847	1090
No. of restraints	0	58	0
$(\Delta/\sigma)_{max}$	0.001	0.001	0.002
$\Delta\rho_{max}$, $\Delta\rho_{min}$ (e Å^{-3})	0.26, -0.28	0.94, -0.66	0.66, -0.40

Complex	$({}^{tBu_2,Dipp}L)_2Ca(DMAP)_2$	$({}^{tBu_2,Ar*}L)Mg(N'')(thf)$	$({}^{tBu_2,Ar*}L)Mg(ODipp)(thf)$ **(7)**
**Crystal data**			
Chemical formula	$C_{68}H_{96}CaN_6O_2{\cdot}C_4H_8O$	$C_{58}H_{74}MgN_2O_2Si_2$	$C_{64}H_{73}MgNO_3$
M_r	1141.69	911.68	928.54
Crystal system, space group	Triclinic, $P\bar{1}$	Monoclinic, *Pn*	Orthorhombic, *Pbca*
Temperature (K)	150	150	150
a, *b*, *c* (Å)	13.0832 (4), 14.2947 (4), 20.3554 (6)	12.6887 (7), 18.1602 (7), 23.5272 (7)	12.1803 (4), 19.9874 (8), 44.7192 (14)
α, *β*, *γ* (°)	71.761 (3), 75.921 (3), 73.267 (3)	90, 91.519 (4), 90	90, 90, 90
V (Å^3)	3413.08 (19)	5419.5 (4)	10887.0 (7)
Z	2	4	8
Radiation type	Cu *K*a	Cu *K*a	Cu *K*a
μ (mm^{-1})	1.16	1.02	0.62
Crystal size (mm)	0.31 × 0.21 × 0.12	0.20 × 0.12 × 0.07	0.17 × 0.11 × 0.02
**Data Collection**			
Diffractometer	SuperNova, Dual, Cu at zero, Atlas	SuperNova, Dual, Cu at zero, Atlas	SuperNova, Dual, Cu at zero, Atlas
Absorption correction	Gaussian *CrysAlis PRO* 1.171.41.93a (Rigaku Oxford Diffraction, 2020) Numerical absorption correction based on gaussian integration over a multifaceted crystal model Empirical absorption correction using spherical harmonics, implemented in SCALE3 ABSPACK scaling algorithm.	Gaussian *CrysAlis PRO* 1.171.41.93a (Rigaku Oxford Diffraction, 2020) Numerical absorption correction based on gaussian integration over a multifaceted crystal model Empirical absorption correction using spherical harmonics, implemented in SCALE3 ABSPACK scaling algorithm.	Gaussian *CrysAlis PRO* 1.171.41.93a (Rigaku Oxford Diffraction, 2020) Numerical absorption correction based on gaussian integration over a multifaceted crystal model Empirical absorption correction using spherical harmonics, implemented in SCALE3 ABSPACK scaling algorithm.
T_{min}, T_{max}	0.409, 1.000	0.789, 1.000	0.774, 1.000
No. of measured, independent and observed [$I > 2\sigma(I)$] reflections	36786, 13918, 11816	21038, 11722, 8639	43502, 15677, 11633
R_{int}	0.037	0.066	0.078
**Refinement**			
$R[F^2 > 2\sigma(F^2)]$, $wR(F^2)$, S	0.088, 0.279, 1.08	0.065, 0.187, 1.03	0.087, 0.246, 1.05
No. of reflections	13918	11722	11074
No. of parameters	785	1197	648
No. of restraints	29	2	0
$(\Delta/\sigma)_{max}$	< 0.001	<0.001	< 0.001
$\Delta\rho_{max}$, $\Delta\rho_{min}$ (e Å^{-3})	1.92, -0.65	0.63, -0.30	0.85, -0.50
Absolute structure	-	Classical Flack method.	-
Absolute structure parameter	-	-0.02 (4)	-

Complex	$(^{Dipp}L)_3Ca_2N''(thf)$ **(8)**	$(^{Dipp}L)_6Sr_3$ **(9)**
**Crystal data**		
Chemical formula	$C_{74}H_{100}Ca_2N_4O_4Si_2$	$C_{114}H_{132}N_6O_6Sr_3$
M_r	1245.91	1945.11
Crystal system, space group	1245.91	Trigonal, $R\bar{3}:H$
Temperature (K)	150	150
a, *b*, *c* (Å)	13.8480 (2), 20.0997 (4), 27.0356 (6)	21.5580 (2), 21.5580 (2), 19.1101 (2)
α, *β*, *γ* (°)	97.719 (2), 90.232 (1), 90.040 (1)	90, 90, 90
V (Å^3)	7456.9 (2)	7691.49 (16)
Z	4	3
Radiation type	Cu *K*a	Cu *K*a
μ (mm^{-1})	1.99	2.44
Crystal size (mm)	0.18 × 0.14 × 0.02	0.18 × 0.16 × 0.02
**Data Collection**		
Diffractometer	SuperNova, Dual, Cu at home/near, Atlas	SuperNova, Dual, Cu at zero, Atlas
Absorption correction	Gaussian<br>CrysAlis PRO 1.171.41.93a (Rigaku Oxford Diffraction, 2020) Numerical absorption correction based on gaussian integration over a multifaceted crystal model Empirical absorption correction using spherical harmonics, implemented in SCALE3 ABSPACK scaling algorithm.	Gaussian<br>*CrysAlis PRO* 1.171.41.93a (Rigaku Oxford Diffraction, 2020) Numerical absorption correction based on gaussian integration over a multifaceted crystal model Empirical absorption correction using spherical harmonics, implemented in SCALE3 ABSPACK scaling algorithm.
T_{min}, T_{max}	0683, 1.000	0.658, 1.000
No. of measured, independent and observed [$I > 2σ(I)$] reflections	48271, 22397, 17758	31991, 16003, 10838
R_{int}	0.042	0.040
**Refinement**		
$R[F^2 > 2σ(F^2)]$, $wR(F^2)$, S	0.061, 0.174, 1.04	0.058, 0.184, 1.14
No. of reflections	22397	3488
No. of parameters	1615	198
No. of restraints	47	0
$(Δ/σ)_{max}$	0.001	< 0.001
$Δρ_{max}$, $Δρ_{min}$ (e Å^{-3})	0.96, -0.38	2.93, -0.61

Computer programs: *CrysAlis PRO* 1.171.39.46 (Rigaku Oxford Diffraction, 2018) *CrysAlis PRO*, Agilent Technologies, Version 1.171.35.21 (release 20-01-2012 CrysAlis171 .NET) (compiled Jan 23 2012,18:06:46); *CrysAlis PRO*, Agilent Technologies, Version 1.171.35.21 (release 20-01-2012 CrysAlis171 .NET) (compiled Jan 23 2012, 18:06:46);

SUPERFLIP. Palatinus, L.; Chapuis, G. J. Appl. Cryst. 2007, 40, 786-790; Sir-92. Altomare, A.; Cascarano, G.; Giacovazzo, C.; Guagliardi, A. J. Appl. Cryst. 1994, 27, 435; *SIR92* Altomare, A.; Cascarano, G.; Giacovazzo, C.; Guagliardi, A. J. Appl. Cryst. 1994, 27, 435, *SHELXL2018*/3 (Sheldrick, 2018); *SHELXL2014* (Sheldrick, 2014); *SHELXS97* (Sheldrick, 1990); *SHELXL2013* (Sheldrick, 2013); XCIF (Sheldrick, 2001); *ORTEP-3 for Windows*. Farrugia, L. J. J. Appl. Cryst. 1997, 30, 565, *CRYSTALS* (Betteridge *et al.*, 2003)..

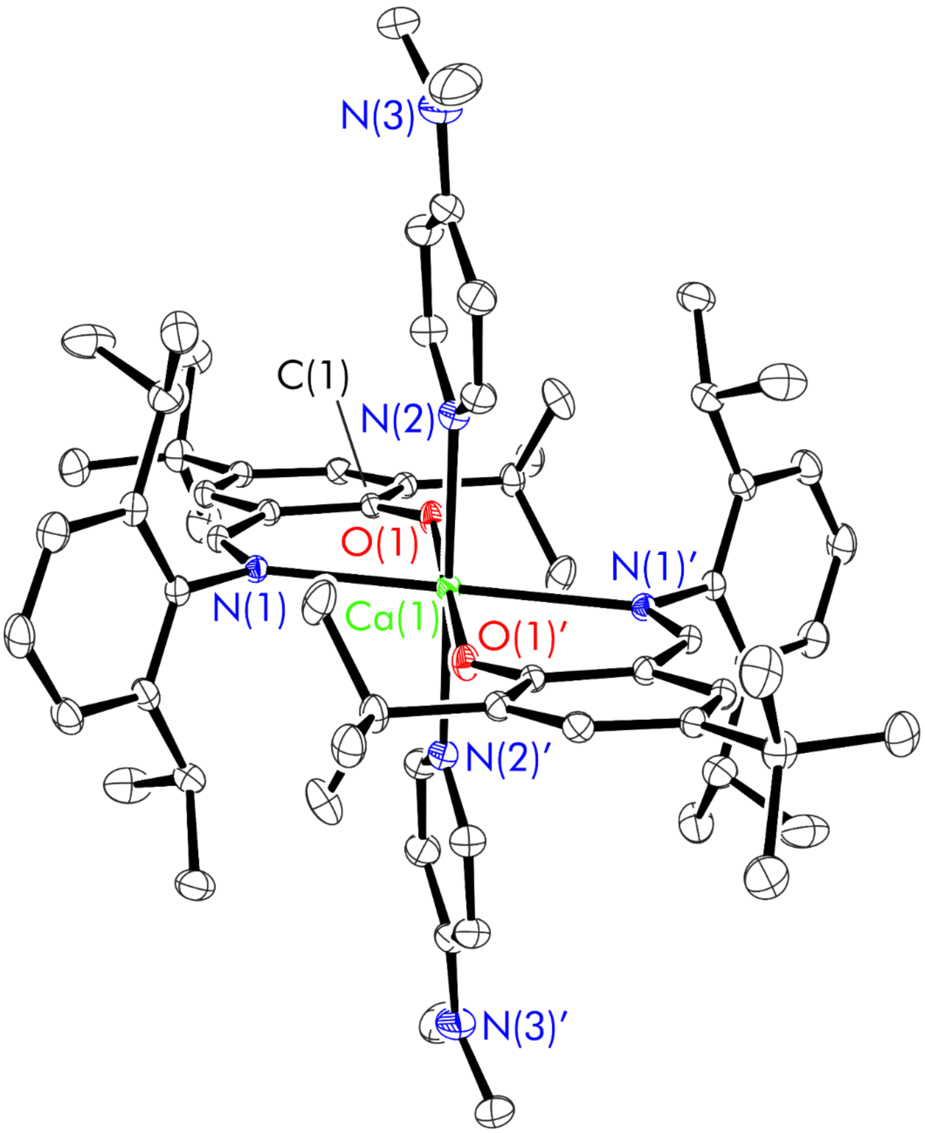

Figure S39. Thermal displacement ellipsoid drawing (30%) of $(^{tBu2,Dipp}L)_2Ca(DMAP)_2$. Symmetry equivalent atoms are denoted with a prime and have been generated by applying the operations -x+1, -y+1, -z or -x+2, -y+2, -z+1. Only one independent molecule in the asymmetric unit is depicted for clarity.

Table S2. Selected metrical data for $(^{tBu2,Dipp}L)_2Ca(DMAP)_2$. Bond lengths in Å and bond angles in °. Values for only one molecule in the asymmetric unit are shown. ' denotes symmetry equivalent atom.

Ca(1)-N(1)	2.577(2)
Ca(1)-N(2)	2.497(3)
Ca(1)-O(1)	2.212(2)
O(1)-Ca(1)-N(1)	74.34(8)
N(1)-Ca(1)-N(2)	89.49(9)
O(1)-Ca(1)-O(1)'	180.0
N(1)-Ca(1)-N(1)'	180.0

V. Density functional theory

DFT calculations were performed with the ORCA program package.[19] The geometry optimizations of the complexes and single-point calculations on the optimized geometries were carried out at the B3LYP[20-22] level of DFT using the def2-SZVP(-f) basis set.[23, 24] The RI[25-27] approximation was used to accelerate the calculations.

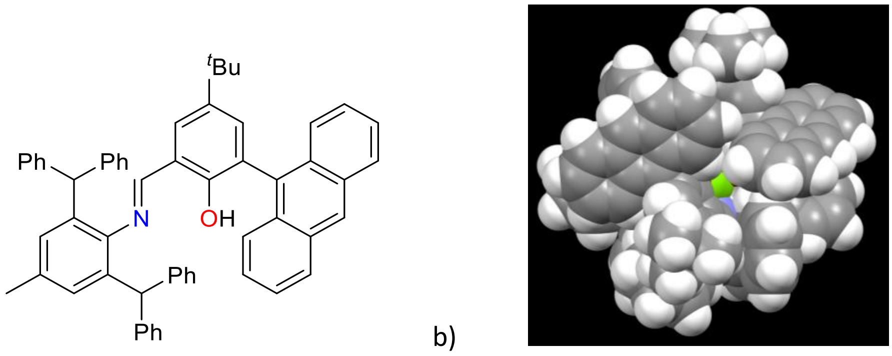

a) b)

Figure S40. a) Structure of $H^{tBu,Ant,Ar^{‡}}L$ and b) the space filling model of $(^{tBu,Ant,Ar^{‡}}L)_2Mg$ (L = $[O\text{-}2\text{-}\{C(H)=N\text{-}2,6\text{-}CHPh_2\text{-}C_6H_3\}\text{-}4\text{-}^{t}Bu\text{-}6\text{-}Ant\text{-}C_6H_2]^{-}$, Ant = $C_{14}H_9$).

<u>Input file</u>

#geometry optimization

!RKS B3LYP RIJCOSX SlowConv TightSCF def2-SVP def2-SVP/J Normalprint Opt

*xyz 0 1

#Coordinates

*

VI. References

(1) García-Valle, F. M.; Estivill, R.; Gallegos, C.; Cuenca, T.; Mosquera, M. E. G.; Tabernero, V.; Cano, J. Metal and Ligand-Substituent Effects in the Immortal Polymerization of rac-Lactide with Li, Na, and K Phenoxo-imine Complexes. *Organometallics* **2015**, *34* (2), 477-487. DOI: 10.1021/om501000b.

(2) Sarazin, Y.; Howard, R. H.; Hughes, D. L.; Humphrey, S. M.; Bochmann, M. Titanium, zinc and alkaline-earth metal complexes supported by bulky O,N,N,O-multidentate ligands: syntheses, characterisation and activity in cyclic ester polymerisation. *Dalton Trans.* **2006**, (2), 340-350, 10.1039/B511851D. DOI: 10.1039/B511851D.

(3) Clark, D. L.; Watkin, J. G.; Huffman, J. C. Preparation and x-ray crystal structure of potassium tetrakis(2,6-diisopropoxyphenolato)neodymate(1-): a one-dimensional chain of lanthanide aryloxide anions joined by bis(.eta.6-arene)-potassium interactions. *Inorg. Chem.* **1992**, *31* (9), 1554-1556. DOI: 10.1021/ic00035a006.

(4) Chang, S.; Jones, L.; Wang, C.; Henling, L. M.; Grubbs, R. H. Synthesis and Characterization of New Ruthenium-Based Olefin Metathesis Catalysts Coordinated with Bidentate Schiff-Base Ligands. *Organometallics* **1998**, *17* (16), 3460-3465. DOI: 10.1021/om970910y.

(5) Åhman, J.; Somfai, P. An Efficient Preparation of Potassium Bis (Trimethylsilyl) Amide (KHMDS). *Synth. Commun.* **1995**, *25* (15), 2301-2303. DOI: 10.1080/00397919508011786.

(6) Cameron, T. M.; Xu, C.; Dipasquale, A. G.; Rheingold, A. L. Synthesis and Structure of Strontium and Barium Guanidinates and Mixed-Ligand Guanidinate Pentamethylcyclopentadienyl Complexes. *Organometallics* **2008**, *27* (7), 1596-1604. DOI: 10.1021/om701118j.

(7) Hu, X.; Dai, S.; Chen, C. Ethylene polymerization by salicylaldimine nickel(ii) complexes containing a dibenzhydryl moiety. *Dalton Trans.* **2016**, *45* (4), 1496-1503, 10.1039/C5DT04408A. DOI: 10.1039/C5DT04408A.

(8) Cosier, J.; Glazer, A. M. A nitrogen-gas-stream cryostat for general X-ray diffraction studies. *J. Appl. Crystallogr.* **1986**, *19* (2), 105-107. DOI: doi:10.1107/S0021889886089835.

(9) CrysAlisPRO, Oxford Diffraction /Agilent Technologies UK Ltd, Yarnton, England. (accessed.

(10) Altomare, A.; Cascarano, G.; Giacovazzo, C.; Guagliardi, A. Completion and refinement of crystal structures with SIR92. *J. Appl. Crystallogr.* **1993**, *26* (3), 343-350. DOI: doi:10.1107/S0021889892010331.

(11) Palatinus, L.; Chapuis, G. SUPERFLIP - a computer program for the solution of crystal structures by charge flipping in arbitrary dimensions. *J. Appl. Crystallogr.* **2007**, *40* (4), 786-790. DOI: doi:10.1107/S0021889807029238.

(12) Sheldrick, G. SHELXT - Integrated space-group and crystal-structure determination. *Acta Crystallogr., Sect. A: Found. Crystallogr.* **2015**, *71* (1), 3-8. DOI: doi:10.1107/S2053273314026370.

(13) Farrugia, L. WinGX suite for small-molecule single-crystal crystallography. *J. Appl. Crystallogr.* **1999**, *32* (4), 837-838. DOI: doi:10.1107/S0021889899006020.

(14) Betteridge, P. W.; Carruthers, J. R.; Cooper, R. I.; Prout, K.; Watkin, D. J. CRYSTALS version 12: software for guided crystal structure analysis. *J. Appl. Crystallogr.* **2003**, *36* (6), 1487. DOI: doi:10.1107/S0021889803021800.

(15) Sheldrick, G. Crystal structure refinement with SHELXL. *Acta Crystallogr. Sect. C: Cryst. Struct. Commun.* **2015**, *71* (1), 3-8. DOI: doi:10.1107/S2053229614024218.

(16) Farrugia, L. ORTEP-3 for Windows - a version of ORTEP-III with a Graphical User Interface (GUI). *J. Appl. Crystallogr.* **1997**, *30* (5 Part 1), 565. DOI: doi:10.1107/S0021889897003117.

(17) Spek, A. Single-crystal structure validation with the program PLATON. *J. Appl. Crystallogr.* **2003**, *36* (1), 7-13. DOI: doi:10.1107/S0021889802022112.

(18) Saxena, P.; Murugavel, R. Bulky 2,6-Dibenzhydryl-4-methylaniline Derived Schiff Base Complexes of Pd(II), Cu(II) and Co(II) as Efficient Catalysts for Suzuki Coupling and Alcohol Oxidation Reactions. *ChemistrySelect* **2017**, *2* (13), 3812-3822. DOI: https://doi.org/10.1002/slct.201700657.

(19) Neese, F. *Orca − an ab initio, DFT and Semiempirical Electronic Structure Package, Version2.8, Revision 2360; Institut für Physikalische und Theoretische Chemie, Universität Bonn, Bonn (Germany)*. 2011. (accessed.
(20) Becke, A. D. Density functional calculations of molecular bond energies. *J. Chem. Phys.* **1986**, *84* (8), 4524-4529. DOI: doi:http://dx.doi.org/10.1063/1.450025.
(21) Lee, C.; Yang, W.; Parr, R. G. Development of the Colle-Salvetti correlation-energy formula into a functional of the electron density. *Physical Review B* **1988**, *37* (2), 785-789.
(22) Becke, A. D. Density-functional thermochemistry. III. The role of exact exchange. *J. Chem. Phys.* **1993**, *98* (7), 5648-5652. DOI: doi:http://dx.doi.org/10.1063/1.464913.
(23) Schäfer, A.; Horn, H.; Ahlrichs, R. Fully optimized contracted Gaussian basis sets for atoms Li to Kr. *J. Chem. Phys.* **1992**, *97* (4), 2571-2577. DOI: 10.1063/1.463096 (acccessed 11/29/2024).
(24) Weigend, F.; Ahlrichs, R. Balanced basis sets of split valence, triple zeta valence and quadruple zeta valence quality for H to Rn: Design and assessment of accuracy. *Physical Chemistry Chemical Physics* **2005**, *7* (18), 3297-3305, 10.1039/B508541A. DOI: 10.1039/B508541A.
(25) Eichkorn, K.; Weigend, F.; Treutler, O.; Ahlrichs, R. Auxiliary basis sets for main row atoms and transition metals and their use to approximate Coulomb potentials. *Theor. Chem. Acc.* **1997**, *97* (1-4), 119-124. DOI: 10.1007/s002140050244.
(26) Eichkorn, K.; Treutler, O.; Öhm, H.; Häser, M.; Ahlrichs, R. Auxiliary basis sets to approximate Coulomb potentials (Chem. Phys. Letters 240 (1995) 283-290). *Chem. Phys. Lett.* **1995**, *242* (6), 652-660. DOI: http://dx.doi.org/10.1016/0009-2614(95)00838-U.
(27) Eichkorn, K.; Treutler, O.; Öhm, H.; Häser, M.; Ahlrichs, R. Auxiliary basis sets to approximate Coulomb potentials. *Chem. Phys. Lett.* **1995**, *240* (4), 283-290. DOI: http://dx.doi.org/10.1016/0009-2614(95)00621-A.
